# Inflammatory responses in primary muscle cell cultures in Atlantic salmon (*Salmo salar*)

**DOI:** 10.1186/1471-2164-14-747

**Published:** 2013-11-01

**Authors:** Nicholas J Pooley, Luca Tacchi, Christopher J Secombes, Samuel AM Martin

**Affiliations:** 1 Institute of Biological and Environmental Sciences, University of Aberdeen, Tillydrone Avenue, Aberdeen AB24 2TZ, UK; 2 Current address: Centre for Evolutionary and Theoretical Immunology, University of New Mexico, Albuquerque, NM 87131-0001, USA

**Keywords:** Transcriptomics, Atlantic salmon (*Salmo salar*), Muscle cell culture, Inflammation, Catabolism, Cell cycle, IGF binding proteins

## Abstract

**Background:**

The relationship between fish health and muscle growth is critical for continued expansion of the aquaculture industry. The effect of immune stimulation on the expression of genes related to the energy balance of fish is poorly understood. In mammals immune stimulation results in major transcriptional changes in muscle, potentially to allow a reallocation of amino acids for use in the immune response and energy homeostasis. The aim of this study was to investigate the effects of immune stimulation on fish muscle gene expression.

**Results:**

Atlantic salmon (*Salmo salar*) primary muscle cell cultures were stimulated with recombinant (r)IL-1β, a major proinflammatory cytokine, for 24 h in order to simulate an acute immune response. The transcriptomic response was determined by RNA hybridization to a 4 × 44 K Agilent Atlantic salmon microarray platform. The rIL-1β stimulation induced the expression of genes related to both the innate and adaptive immune systems. In addition there were highly significant changes in the expression of genes related to regulation of the cell cycle, growth/structural proteins, proteolysis and lipid metabolism. Of interest were a number of IGF binding proteins that were differentially expressed, which may demonstrate cross talk between the growth and immune systems.

**Conclusion:**

We show rIL-1β modulates the expression of not only immune related genes, but also that of genes involved in processes related to growth and metabolism. Co-stimulation of muscle cells with both rIGF-I and rIL-1β demonstrates cross talk between these pathways providing potential avenues for further research. This study highlights the potential negative effects of inflammation on muscle protein deposition and growth in fish and extends our understanding of energy allocation in ectothermic animals.

## Background

Muscle growth involves a tightly controlled balance between protein synthesis and degradation [1]. Protein synthesis is driven by the growth hormone (GH)/Insulin like growth factor (IGF)/mammalian target of rapamycin (mTOR) pathway [2-5], whereas protein degradation occurs via a number of pathways including ubiquitin proteasome [6-8], lysosomal [9], apoptotic [10] and the calcium dependant calpains [11]. These processes and the pathways underlying their regulation have been examined in Atlantic salmon (*Salmo salar*) [12], rainbow trout (*Oncorhynchus mykiss*) [13-16] and other fish [17,18]. The anabolic effects of the GH/IGF system have also been studied in ectothermic animals including Atlantic salmon [12,19,20], rainbow trout [21,22] and other teleosts [23]. The GH/IGF system has been seen to activate the mTOR pathway thus directing protein synthesis, and is highly conserved in teleosts [2-4].

In mammals the key signals involved in stimulating anabolic activity are free amino acids, GH and IGF [24], whereas catabolic signals include nutrient depletion, hormones such as cortisol and transcription factors such as forkhead box O (FOXOs) [25]. The actions of many of these key signals have been seen to be conserved in salmonid fish [2,12,22]. Despite being initiated by different signals, catabolism and anabolism share many aspects of downstream signalling machinery, providing the possibility of intracellular cross talk between these two processes [26]. In mammals undergoing acute inflammatory responses, muscle tissue goes into immediate catabolic state [27,28] where muscle fibres are broken down releasing free amino acids, likely to be used for liver protein synthesis of acute phase serum proteins. As skeletal muscle is the principal body store of proteins, this tissue is the main target for catabolism and release of free amino acids [29]. In mammals the inflammatory response leads to a loss of skeletal muscle mass in both acute and chronic inflammatory situations [30]. The current consensus in higher vertebrates is that this increase in muscle atrophy can be mediated by proinflammatory cytokines such as interleukin-1β (IL-1β) [26,31], IL-6 [27,32,33] and tumor necrosis factor-α (TNF-α) [26,27,32]. Several different processes have been identified by which proinflammatory cytokines can negatively affect muscle mass. IL-1β and TNFα receptors, on the surface of the cells, signal via conserved signal transduction pathways and alter gene expression, which in muscle tissue normally induces genes involved in protein degradation resulting in the release of free amino acids [28,31,34-36]. In parallel this cytokine signalling competes with and decreases the effects of IGF-I signalling, specifically during downstream signal transduction, hence reducing the anabolic hormone effect. Such intracellular receptor crosstalk between cytokines and anabolic hormones can lead to a state of endocrine resistance whereby no increase in the amount of ligand present will increase the hormonal effects [26,37,38]. This cytokine induced hormone resistance can result in a condition known as cachexia, one aspect of which is a chronic increase in proinflammatory cytokines such as TNFα and IL-1β [39,40]. The effects of cachexia are a loss of body mass, especially skeletal muscle protein, and it is thought that the ability of cytokines to cause hormone resistance is one of the primary mediators of cachexia. This condition differs from simple weight loss since the loss of body mass will continue despite feeding [26,39].

Transcriptional responses to various triggers of protein catabolism have been examined in salmonid fish, including starvation [41], starvation and refeeding [42], or following extensive anorexic migrations [18] and vitellogenesis [14,43]. However to date only a limited number of investigations have addressed the effects of infection or immune stimulation on muscle growth in fish [44,45]. Previously a cachexia model in rainbow trout was developed by chronic stimulation with lipopolysaccharides (LPS) [44], mimicking sepsis and chronic background infection. In these fish, muscle protein content was decreased, but levels of MyoD and myosin were unaffected indicating that while muscle accretion was altered, the mechanisms may be different to those known in mammals. In general the response was much less dramatic than is observed in mammals, probably reflecting the different control of amino acid reallocation in ectothermic fish.

Proinflammatory cytokines, which include IL-1β, are the primary mediators of the innate immune system [46] and show a rapid response at the transcriptional level following recognition of pathogens including bacterial and viral products [47]. IL-1β is secreted as the mature form following cleavage of the precursor molecule by interleukin 1 converting enzyme (ICE). The mature soluble protein binds to the IL-1 receptor 1 (IL-1R1) receptor which then recruits the IL-1 receptor accessory protein (IL-1RAcP) and initiates the signal cascade [47,48]. The signalling cascade activates pathways that positively regulate the activity of transcription factor nuclear factor-κβ (NFκB) and the mitogen activated protein kinases p38 (MAPK p38) and c-Jun N-terminal kinases (JNK) [26,48,49]. It is through the activation of these pathways that IL-1β is thought to negatively affect anabolism while stimulating catabolism [26,34,37]. Whilst there is some controversy as to how IL-1β is processed in fish [50-52], nevertheless a functional mature peptide has been produced in several species [53] and the receptor genes have been cloned [54,55].

This paper investigates the effects of acute proinflammatory stimulation on the transcriptome of Atlantic salmon primary myocyte cells. We hypothesise that the inflammatory stimuli will cause significant changes in the expression of genes related to immune function, protein metabolism and other cellular processes. Further to this, we hypothesize that co-incubation of cell cultures with IGF-I as well as rIL-1β will lead to an attenuation of the metabolic actions of inflammation.

## Results

### Cell culture and stimulation

Primary muscle cell cultures were assessed for differentiation and purity by light microscopy at 4× and 10× magnification (Data not shown). Nine grams of white skeletal muscle pooled from six fish provided sufficient cells to reach confluence when evenly split between two 6 well plates. Prior to performing the microarray analysis, confirmation that the cells responded to rIL-1β was carried out by real time PCR using IL-1β itself as a marker gene since it is known to increase in expression in response to rIL-1β stimulation. IL-1β expression was significantly increased (541 fold) in the stimulated samples compared to the control samples.

### Microarray analysis

Following filtering and quality control 27458 probes were retained for statistical analysis. Of these 7649 were significantly altered in expression at P < 0.05 following correction for multiple tests. We further filtered this set of genes by retaining those with a fold change of >2 leaving a differentially regulated set of 2504 genes for analysis (Table 1, full list of genes Additional file 1: Table S1). Within the gene set 1209 features were increased and 1295 features decreased in expression. The gene with the highest up-regulation is the cytokine TNFα2 with a 216 fold increase, whilst aquaporin 1 was the most decreased in expression with a 125 fold reduction in expression. Confirmation of microarray expression was conducted using seven key genes analysed with realtime PCR (Additional file 2: Figure S1) where a highly significant correlation (r^2^ = 0.8763 & P < 0.001) between qPCR and microarray data was found.

**Table 1:** Microarray analysis showing the numbers of transcripts found to be differentially expressed following stimulation of primary muscle cells by rIL-1β compared to unstimulated cells with various P value (corrected by Benjamini Hochberg FDR) and Fold change (FC) cutoffs

	**P all**	**P < 0.05**	**P < 0.02**	**P < 0.01**	**P < 0.005**	**P < 0.001**
FC all	27458	7649	3945	1912	591	0
FC > 1.1	21039	7592	3938	1910	591	0
FC > 1.5	7430	4752	3039	1630	554	0
FC > 2.0	3205	2504	1884	1167	455	0
FC > 3.0	1275	1131	956	695	317	0
Expected by chance		382	78	19	2	0

**Figure 1:**
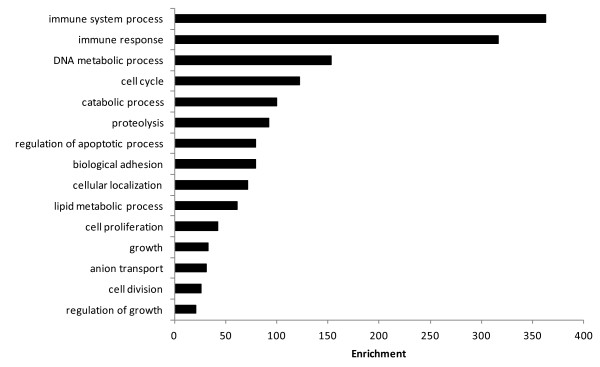
**Bar chart showing the 15 Gene ontologies found to be most highly statistically enriched in response to rIL-1β stimulation of muscle cells *in vitro*.** Gene ontology enrichment carried out using GOEAST, GOslimming of the subsequent list performed with REVIGO.

In order to better understand the changes in whole cell transcriptomic output, gene ontology analysis was used to indicate the biological processes that were modulated by the IL-1β stimulation. From the 2504 features retained for analysis, 2196 (88%) were annotated to a functional protein and 1945 (78%) were assigned at least one gene ontology (GO) identifier for biological process, enabling further assessment of biological function. These proportions reflect the annotation of all features on the microarray slide. Statistical analysis for enrichment for biological processes resulted in 1195 biological process GO terms being identified. The nature of GO analysis means that many of these are overlapping and only the non-redundant major groupings are presented (Figure 1). Observation of both the GO analysis and manual assignment identification of functions was used to assign genes to functional groups. The differentially expressed genes could be defined as belonging to a number of distinct functional classes, especially immune response, proteolysis, growth regulation and structural proteins, cell cycle and lipid metabolism. The directional expression changes indicated how these processes were being affected, with a general increase in the expression of immune related and protein metabolism genes, whereas growth, structural proteins and cell cycle showed a negative trend, with a majority of genes being down regulated in expression. A complex response was found for genes encoding lipid metabolism proteins, indicting major transcriptional changes relating to lipid mobilisation.

### Immune response genes

There was a clear increase in genes related to immune function (Table 2) most notably in the high increase of expression of mRNAs encoding proinflammatory cytokines such as IL-1β and TNFα (1/2) as well as chemokines such as IL-8. Transcription factors involved in IL-1β signalling were also increased in expression with subunits of NFκB and its inhibitor, MAP kinase-interacting serine/threonine kinase 2, MAPK activated jun-B and CCAAT/enhancer binding protein all being up regulated. Components of the IL-1β receptor machinery were also increased including IL-1 receptor accessory protein, IL-1 receptor kinase and an IL-1 receptor antagonist protein mRNA (Table 2). Other innate immune related genes were also increased including complement components, C-type lectins and the antimicrobial proteins hepcidin and ferritin. Both these latter two genes have roles in iron binding. Several negative regulators of inflammation were also found to be increased including two suppressors of cytokine signalling (SOCS) genes, SOCS 1 and 3, the anti-inflammatory cytokine IL-10, and as mentioned earlier an IL-1 antagonist (nIL-1 F).

**Table 2:** Differential expression of genes related to the immune response

**Gene ID**^**1**^	**Annotation**^**2**^	**Mean fold change± SE**^**3**^	**Identity**^**4**^
Ssa#S24188435	AY929386	216.3 ± 58.8	TNFα 2
Omy#gi185133433	NM_001124347	197.9 ± 79.1	IL-1B
Ssa#STIR00083_4	AY929385	99.2 ± 17.9	TNFα 1
Omy#gi13235345	AJ279069	93.3 ± 15.4	IL-8
Ssa#S34822137	AM397592	29.7 ± 5.1	Complement c3
Ssa#STIR04816	BT047247	25.5 ± 13.1	Hepcidin
Omy#S37211068	EF175381	20.7 ± 1.1	Prostaglandin G/H synthase 2b
Ssa#STIR08688	TC65065	17.9 ± 4.9	Ferritin
Ssa#S21512941	AY572832	16.1 ± 0.2	C type lectin receptor A
Ssa#STIR35259	NM_001124618	13.8 ± 0.9	Complement protein component c7-1
Ssa#STIR23928	NM_001124410.1	13.3 ± 10.8	Complement factor H precursor
Ssa#S43134841_S	NM_001123611	9.5 ± 2.5	CD4-like protein
Ssa#STIR00084_4	DW569632	8.5 ± 0.3	NF-kappa-b inhibitor alpha
Ssa#S18892257	AJ505008	3.9 ± 0.3	IL-1 receptor accessory protein
Ssa#STIR14647	TC73172	3.9 ± 0.0	MAP kinase-interacting serine/threonine kinase 2
Ssa#S30276405	DW563373	3.9 ± 0.2	Suppressor of cytokine signaling 1
Ssa#S35660755	EG895473	3.4 ± 0.3	NF-kappa-B p100 subunit
Ssa#S35667643	EG902361	3.4 ± 0.2	Complement c1q-like protein 4
Ssa#S31992293	DY720890	3.2 ± 0.2	IL-10 receptor beta chain precursor
Omy#gi197927463	NM_001124396	3.0 ± 0.2	IL-1 receptor antagonist
Ssa#KSS392	NM_001141766	2.9 ± 0.3	IL-1 receptor-associated kinase 4
Ssa#CB516003	CB516003	2.8 ± 0.3	NF-kappa-B 1 p105 subunit
Ssa#KSS3660	BT059477	2.8 ± 0.3	NF-kappa-B inhibitor epsilon
Ssa#STIR08793	TC65192	2.8 ± 0.1	Suppressor of cytokine signaling 3
Ssa#STIR00087_4	DW555246	2.7 ± 0.2	IL-10
Ssa#STIR12117	TC69580	2.3 ± 0.1	Transcription factor jun-B
Omy#S18157537	BX883008	2.1 ± 0.1	Interleukin-6 receptor subunit alpha precursor
Ssa#DW006091	DW006091	2.1 ± 0.2	MHC class i antigen
Ssa#TC106540	TC106540	−2.4 ± 0.1	TNF receptor-associated factor 6
Ssa#STIR08822	TC65229	−2.6 ± 0.1	IL-15
Ssa#S18849636_S	BT071912.1	−2.6 ± 0.2	Complement C4-1
Ssa#S35693513	EG928231	−3.3 ± 0.8	Complement component 6 precursor
Ssa#S35687715	EG922433	−22.7 ± 8.3	Complement factor D precursor

List of selected mRNAs associated with the immune response found to be increased or decreased in expression in response to rIL-1β stimulation. Genes were assigned to the table based upon both their GO identifier and previous knowledge of their functions. Genes with greatest fold differences in expression are presented, the genes that are lower in expression are denoted by (-) value. The genes shown were significant at p < 0.05 following t- tests with Benjamini-Hochberg FDR and greater than 2-fold change. ^1^Indicates the unique code for the feature on the microarray, ^2^Accession number of the cDNA sequence, accession numbers beginning with TC are for oligos from the TIGR Atlantic Salmon Gene Index. ^3^Fold change, in the case of oligos that featured multiple times in the gene list the one with the highest fold change is reported. ^4^Identity of the probe target as determined by BLASTX and BLASTN searches.

### Proteolysis

Genes related to protein metabolism were modulated by the IL-1β stimulation including those involved in both synthesis and degradation (Table 3). The largest group of protein metabolism genes found to be increased in expression were those related to proteolysis, specifically the ubiquitin proteasome pathway (UBP). Several E3 ubiquitin ligases, ubiquitin like proteins and four 20S proteasome subunits all increased in expression. Other genes encoding proteolytic proteins found to be increased in expression included collagenase 3 and a cytosolic dipeptidase. A number of proteases were decreased in expression including a subunit of calpain 1, serine protease htra1 and 35, cystatin B and ubiquitin-conjugating enzyme E2 T.

**Table 3:** Differential expression of genes related to proteolysis

**Gene ID**^**1**^	**Annotation**^**2**^	**Mean fold change± SE**^**3**^	**Identity**^**4**^
Ssa#CL13Contig1	CL13Contig1	10.0 ± 1.8	Collagenase 3
Ssa#S30266930	DW553898	8.3 ± 0.4	Angiotensinogen
Ssa#CK884742	CK884742	3.4 ± 0.5	Cytosolic non-specific dipeptidase
Ssa#S35528810	EG815188	3.2 ± 0.1	Ubiquitin-like protein 1
Ssa#S18890005	CB515535	2.8 ± 0.2	E3 ubiquitin-protein ligase RNF144A-A
Ssa#DW564916	DW564916	2.7 ± 0.4	Ubr5 protein
Ssa#CL300Ctg1	CL300Contig1	2.4 ± 0.1	Proteasome subunit beta type-6 precursor
Ssa#KSS4965	KSS4965	2.3 ± 0.1	Proteasome subunit beta type 7b
Ssa#STIR04015	BT048053	2.2 ± 0.1	Proteasomebeta type 8
Ssa#S30295323	DW582287	2.2 ± 0.1	Proteasome subunit alpha type-6
Ssa#S35509463	EG795841	2.0 ± 0.0	E3 ubiquitin-protein ligase CHFR
Ssa#S35533557	EG819935	−2.1 ± 0.1	Ubiquitin-conjugating enzyme E2 T
Ssa#S35507555	EG793933	−2.1 ± 0.2	Calpain small subunit 1
Ssa#S35530808	EG817186	−2.1 ± 0.1	Cystatin-B
Ssa#S31992074	DY720671	−3.8 ± 0.2	Ubiquitin- containing phd and ring finger 1
Ssa#S30242447	NM_001141717	−3.9 ± 0.2	Serine protease htra1
Ssa#S35564994	EG851372	−16.2 ± 0.9	Protease, serine, 35

List of selected mRNAs related to proteolysis found to be increased or decreased in expression in response to rIL-1β stimulation. Genes were assigned to the table based upon both their GO identifier and previous knowledge of their functions. Genes with greatest fold differences in expression are presented, the genes that are lower in expression are denoted by (-) value. The genes shown were significant at p < 0.05 following t- tests with Benjamini-Hochberg FDR and greater than 2-fold change. ^1^Indicates the unique code for the feature on the microarray, ^2^ Accession number of the cDNA sequence, accession numbers beginning with TC are for oligos from the TIGR Atlantic Salmon Gene Index. ^3^Fold change, in the case of oligos that featured multiple times in the gene list the one with the highest fold change is reported. ^4^Identity of the probe target as determined by BLASTX and BLASTN searches.

### Growth regulation and structural proteins

An interesting group of genes that can be regarded as controllers of anabolic signalling were also modulated. Most notable were the IGF binding proteins (IGFBPs), where IGFBP-6 was found increased in expression following the inflammatory stimulus whereas IGFBPs -4, 5 and rP1 decreased in expression (Table 4). Genes controlling muscle cell differentiation were also changed in expression including the transcriptional repressor yin-yang 1 (YY1) which showed an up regulation and myogenic regulatory factor 5 (MyF5) which was down regulated (Table 4). Structural protein encoding mRNAs showed a marked tendency to be down regulated, as seen with the collagens and the myosins, β-actin, and troponin (Table 4).

**Table 4:** Differential expression of genes for growth regulation & structural proteins

**Gene ID**^**1**^	**Annotation**^**2**^	**Mean fold change± SE**^**3**^	**Identity**^**4**^
Ssa#S30261281	DW548249	17.2 ± 2.5	IGF binding protein 6
Ssa#S22669043	AY462105	4.6 ± 0.2	Growth hormone receptor isoform 1 precursor
Ssa#S35518234	EG804612	2.9 ± 0.2	CCAAT/enhancer binding protein delta
Ssa#DW566454	DW566454	2.6 ± 0.4	Signal transducer and activator of transcription 2
Ssa#STIR16259	TC75448	2.3 ± 0.1	YY1 transcription factor
Ssa#STIR03019	BT049051	−2.1 ± 0.0	Regulator of g-protein signaling 18
SsaHomCont3_080	SsaHomContl3	−2.2 ± 0.3	Beta-actin
Ssa#S30278631	DW565599	−2.2 ± 0.1	Collagen alpha 2 type VI
Omy#TC165689	TC165689	−2.4 ± 0.2	Collagen alpha 2 type V preproprotein
Ssa#S18891260	CB515159	−2.4 ± 0.4	Type I collagen alpha 2 chain
Ssa#S26643985	DQ163908	−2.5 ± 0.2	Growth hormone receptor isoform 2 precursor
Ssa#S35580189	EG866567	−2.5 ± 0.0	Collagen alpha 1 type II isoform 1 precursor
Ssa#STIR17006	TC76573	−2.6 ± 0.2	Growth arrest-specific 1
Ssa#CA041082	CA041082	−2.8 ± 0.2	Transforming growth factor, beta receptor III
Ssa#S35580645	EG867023	−2.8 ± 0.2	Vascular endothelial growth factor D
Ssa#CA037592	CA037592	−2.8 ± 0.2	Myosin IB
Ssa#S31998683	DY727280	−2.8 ± 0.2	Laminin, beta 1
Ssa#S35563089	EG849467	−2.9 ± 0.3	Collagen alpha 1 type V
Ssa#S31977813	DY706603	−3.1 ± 0.1	Myosin phosphatase-Rho interacting protein isoform 1
Ssa#STIR25506	TC89337	−3.1 ± 0.1	Type i keratin s8
Ssa#S46924879	EU861009.1	−3.4 ± 0.1	IGF binding protein 5
Ssa#S32004569	DY733166	−3.5 ± 0.5	Corticotropin releasing factor precursor
Ssa#STIR05529	BT046528	−3.5 ± 0.2	Collagen triple helix repeat containing 1
Ssa#TC91867	TC91867	−4.2 ± 0.3	Collagen alpha 1 type XI isoform A preproprotein
Ssa#S35504964	EG791342	−4.6 ± 0.5	Troponin I, slow skeletal muscle
Ssa#STIR00115_3	BT045917	−4.8 ± 0.6	Tropomyosin-1 alpha chain
Ssa#STIR11900	TC69277	−5.2 ± 0.4	Myosin ic
Ssa#S37580916	EF432866	−5.4 ± 0.7	IGF binding 7 precursor
Ssa#S37580919	EF432861	−7.9 ± 0.4	IGF binding protein 4
Ssa#S35582593	EG868971	−10.9 ± 5.3	Collagen alpha 1 type X precursor
Ssa#STIR30922	TC97553	−27.9 ± 2.7	Myf5 protein

List of selected mRNAs related to growth regulation & structural proteins found to be increased or decreased in expression in response to rIL-1β stimulation. Genes were assigned to the table based upon both their GO identifier and previous knowledge of their functions. Genes with greatest fold differences in expression are presented, the genes that are lower in expression are denoted by (-) value. The genes shown were significant at p < 0.05 following t- tests with Benjamini-Hochberg FDR and greater than 2-fold change. ^1^Indicates the unique code for the feature on the microarray, ^2^ Accession number of the cDNA sequence, accession numbers beginning with TC are for oligos from the TIGR Atlantic Salmon Gene Index. ^3^Fold change, in the case of oligos that featured multiple times in the gene list the one with the highest fold change is reported. ^4^Identity of the probe target as determined by BLASTX and BLASTN searches.

### Cell cycle and DNA metabolism

The expression of genes regulating the cell cycle was clearly altered, with the majority of them being reduced in expression (Table 5). Five cyclins (A2, B1/2, E1/2), two cyclin dependent kinases, and several cell division cycle proteins were all reduced in expression. However, two cyclins (D1 and G2) were increased in expression. DNA metabolism genes were also generally decreased in expression, including several minichromosome maintenance complex components, DNA replication complex and DNA replication licensing factor mcm2.

**Table 5:** Differential expression of genes related to the cell cycle & DNA replication

**Gene ID**^**1**^	**Annotation**^**2**^	**Mean fold change± SE**^**3**^	**Identity**^**4**^
Ssa#S30294618	DW581582	2.4 ± 0.3	Cyclin D1
Ssa#S31971283	DY700073	2.4 ± 0.2	Cell division cycle associated 4 isoform 14
Ssa#S35549130	EG835508	2.2 ± 0.1	Cyclin G2
Ssa#S30291070	DW578034	−2.0 ± 0.1	Cyclin-dependent kinase 2 isoform 1
Ssa#STIR18340	TC78544	−2.1 ± 0.1	Cyclin B1
Omy#S19711047	CR367942	−2.1 ± 0.2	Cyclin B2
Ssa#S35661746	EG896464	−2.2 ± 0.2	Cell cycle progression 1 isoform 2
Ssa#S35547210	EG833588	−2.2 ± 0.1	Mediator of RNA polymerase II transcription subunit 22
Ssa#TC109012	TC109012	−2.6 ± 0.2	Cyclin E1 isoform 1
Ssa#KSS3754	NM_001173741	−2.8 ± 0.2	Minichromosome maintenance complex component 4
Ssa#STIR12008	TC69433	−3.0 ± 0.1	Cell division control protein 2
Ssa#TC103697_S	TC103697	−3.0 ± 0.1	DNA replication licensing factor mcm2
Ssa#S30290620	DW577584	−3.2 ± 0.1	Cyclin-dependent kinase 4
Ssa#S35659383	EG894101	−3.3 ± 0.1	Cyclin A2
Ssa#S18888540	CB514505	−4.2 ± 0.3	Minichromosome maintenance complex component 2
Ssa#S35699881	EG934599	−4.4 ± 0.1	Minichromosome maintenance complex component 3
Ssa#S35664683	EG899401	−4.8 ± 0.4	DNA replication complex GINS protein PSF1
Ssa#S30295467	DW582431	−5.1 ± 0.6	Spindle pole body component 24 homolog
Ssa#STIR15543	TC74419	−5.3 ± 0.5	Minichromosome maintenance complex component 7
Ssa#S18890448	CB516667	−6.7 ± 0.6	Minichromosome maintenance complex component 5
Ssa#S30277130	DW564098	−10.6 ± 0.4	Cell division cycle associated 7 isoform 1
Omy#S19711255	CR367985	−14.7 ± 8.7	Cyclin E2
Omy#S34311297	CU069027	−15.1 ± 2.8	Cell division cycle 6 protein

List of selected mRNAs related to the cell cycle & DNA replication found to be increased or decreased in expression in response to rIL-1β stimulation. Genes were assigned to the table based upon both their GO identifier and previous knowledge of their functions. Genes with greatest fold differences in expression are presented, the genes that are lower in expression are denoted by (-) value. The genes shown were significant at p < 0.05 following t- tests with Benjamini-Hochberg FDR and greater than 2-fold change. ^1^Indicates the unique code for the feature on the microarray, ^2^Accession number of the cDNA sequence, accession numbers beginning with TC are for oligos from the TIGR Atlantic Salmon Gene Index. ^3^Fold change, in the case of oligos that featured multiple times in the gene list the one with the highest fold change is reported. ^4^Identity of the probe target as determined by BLASTX and BLASTN searches.

### Lipid and sterol metabolism

Finally, stimulation with rIL-1β caused changes in the expression of genes involved in lipid metabolism (Table 6). These included the increase in expression of several cholesterol transport proteins such as apolipoprotein (Apo) L3 and lipoprotein lipase. However there was also a down regulation in other similar genes such as Apo A1 binding protein and Apo B and a down regulation of proteins involved in sterol synthesis.

**Table 6:** Differential expression of genes related to lipid and sterol metabolism

**Gene ID**^**1**^	**Annotation**^**2**^	**Mean fold change± SE**^**3**^	**Identity**^**4**^
Ssa#S18890165	CB515874	23.2 ± 2.2	Creatine kinase, ubiquitous mitochondrial precursor
Ssa#STIR00012_4	AY848944	13.3 ± 1.1	Prostaglandin-endoperoxide synthase 2
Ssa#S30259776	DW546744	8.7 ± 0.3	Sphingomyelin synthase 1
Ssa#STIR22551	TC84899	6.3 ± 0.2	Lipoprotein lipase
Ssa#TC105353	TC105353	5.5 ± 1.0	Mecr protein
Ssa#STIR12701	TC70393	3.6 ± 0.1	Retinol dehydrogenase 3
Ssa#STIR31819	TC98944	3.5 ± 0.3	Glucose-6-phosphate-1-dehydrogenase
Ssa#S48420588	NM_001173773	3.2 ± 0.7	Myotubularin
Ssa#S31962884	DY691674	2.9 ± 0.1	Cytochrome c oxidase subunit 5B, mitochondrial
Ssa#S31963491	DY692281	2.8 ± 0.1	PPAR-alpha interacting complex protein 285 isoform 1
Ssa#KSS1976	KSS1976	2.8 ± 0.2	78 kDa glucose-regulated protein
Ssa#S35587721	EG874099	2.7 ± 0.2	Apolipoprotein-L3
Ssa#S30242761	DW538822	2.6 ± 0.2	Glycolipid transfer protein
Ssa#S32012431	DY741028	2.3 ± 0.1	StAR-related lipid transfer domain containing 3
Ssa#CL50Contig2	CL50Contig2	2.3 ± 0.1	Fructose-bisphosphate aldolase A
Ssa#S32007249	DY735846	2.1 ± 0.2	Adipose differentiation-related protein
Ssa#STIR13627	TC71700	−2.0 ± 0.2	Cox18 cytochrome c oxidase assembly homolog
Ssa#CA043659	CA043659	−2.1 ± 0.2	Apolipoprotein B precursor
Ssa#DW564686	DW564686	−2.1 ± 0.1	Mitochondrial uncoupling protein 2
Ssa#STIR21893	TC83911	−2.2 ± 0.1	Creatine kinase b-type
Ssa#S35679641	EG914359	−2.5 ± 0.1	Lipid phosphate phosphohydrolase 1
Ssa#STIR22405	TC84675	−2.6 ± 0.1	Lipase a
Ssa#S30285553	DW572521	−2.6 ± 0.1	Lipid phosphate phosphohydrolase 2
Ssa#S30246050	DW542111	−2.9 ± 0.3	Glyceraldehyde-3-phosphate dehydrogenase-2
Ssa#TC108704	TC108704	−3.8 ± 0.2	Lipocalin precursor
Ssa#STIR21285	TC82989	−4.9 ± 0.3	Glutamine synthetase
Ssa#S35523399	EG809777	−5.2 ± 0.5	Cholesteryl ester transfer protein, plasma
Ssa#STIR22650	TC85053	−5.2 ± 1.4	Apolipoprotein a-i binding protein

List of selected mRNAs related to the lipid & sterol metabolism found to be increased or decreased in expression in response to rIL-1β stimulation. Genes were assigned to the table based upon both their GO identifier and previous knowledge of their functions. Genes with greatest fold differences in expression are presented, the genes that are lower in expression are denoted by (-) value. The genes shown were significant at p < 0.05 following t- tests with Benjamini-Hochberg FDR and greater than 2-fold change. ^1^Indicates the unique code for the feature on the microarray, ^2^Accession number of the cDNA sequence, accession numbers beginning with TC are for oligos from the TIGR Atlantic Salmon Gene Index. ^3^Fold change, in the case of oligos that featured multiple times in the gene list the one with the highest fold change is reported. ^4^Identity of the probe target as determined by BLASTX and BLASTN searches.

### Temporal response and interaction of IGF and IL-1β

**Figure 2:**
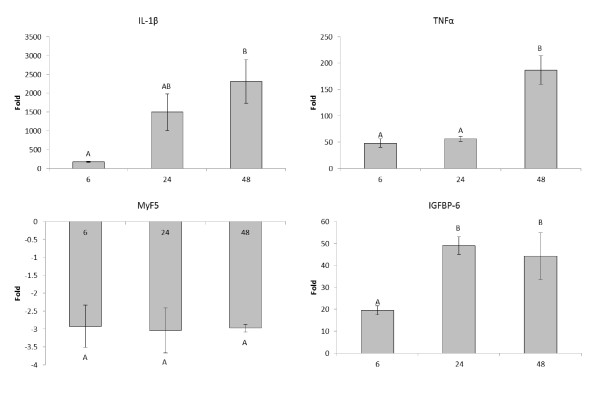
**Graph showing the fold effects of rIL-1β stimulation compared to control on the expression of genes involved in the immune response and growth after 6, 24 and 48 h stimulation.** Statistics were carried out using a one way ANOVA. Time points that do not share a letter are statistically different from each other. All mRNAs examined here were significantly altered in expression at all time points relative to the unstimulated control.

To assess the effect of time of rIL-1β stimulation on primary myocytes on gene expression, rIL-1β stimulation was performed at 6, 24 and 48 h and four key marker genes from the microarray analysis (IL-1β, TNFα, MyF5 and IGFBP-6) were examined by real time PCR (Figure 2). IL-1β was highly increased in expression at all-time points but it was at 48 h that the highest increase in expression was found. TNFα also showed the greatest fold increase at 48 h however this was more due to a reduction in the control expression seen at 48 h, than an increase in the stimulated cells. MyF5 was consistently down regulated at all time points with no increase in effect seen after 6 h. Finally IGFBP-6 was increased at all 3 times, but with a maximum fold increase at 24 h and 48 h.

To assess the interaction between rIL-1β and rIGF-I primary myocyte cultures were stimulated with rIL-1β (25 ng/ml), rIGF-I (100nM), rIL-1β (25 ng/ml) + rIGF-I (100nM) or maintained as control. These stimulations were carried out for both 6 h and 24 h to determine if rIL-1β interfered with early effects that IGF-I may have on the cell cultures. The genes analysed were chosen to represent the immune response (IL-1β, TNFα and hepcidin) and protein metabolism/growth (atrogin-1, MyF5, IGFBPs-4, 5 & 6).

**Figure 3:**
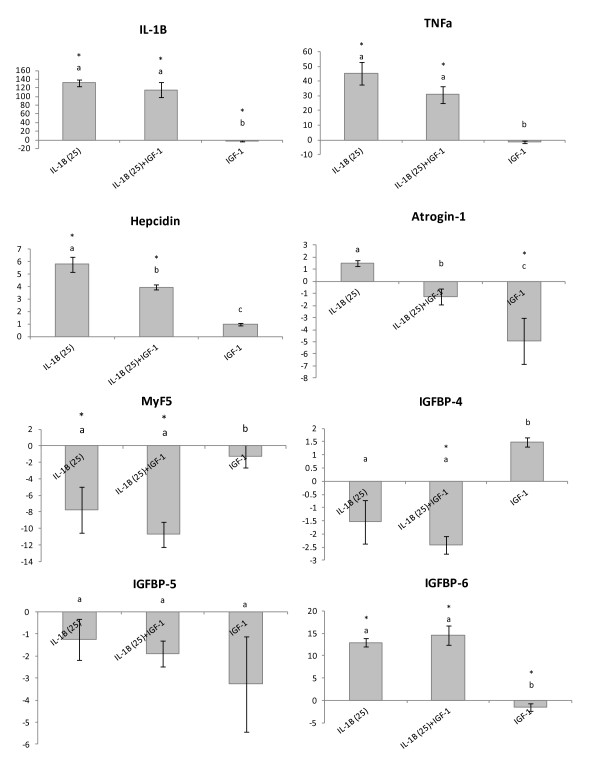
**Fold change of genes involved in both the immune response and growth in response to 6 h stimulation with either rIL-1β (25 ng/ml), rIL-1β (25 ng/ml) + rIGF(100nM), or rIGF(100nM).** * represents a significant difference from control, bars which share a letter are not significantly different. All fold changes were calculated compared to unstimulated control samples. Comparative gene expression was measured with qPCR.

At 6 h co-stimulation of cells there was an up regulation of IL-1β and TNFα expression in response to rIL-1β stimulation, and this was not significantly altered by co-incubation with rIL-1 β + rIGF-I (Figure 3). Hepcidin was also found to be up regulated in response to rIL-1β (5.8 fold), with co-incubation with rIL-1β + rIGF-I reducing the magnitude of this increase ~30% (Figure 3). Regarding the expression of the IGFBPs, there was no effect of any treatment on the expression of IGFBP-5. IGFBP-6 was up regulated in response to rIL-1β (13.0 fold) and this effect was not altered by co-incubation with rIGF-I. However, stimulation with just rIGF-I led to a significant reduction in the expression of IGFBP-6 (Figure 3). Curiously IGFBP-4 was found to be significantly down regulated in response to co-incubation with rIL-1β + rIGF-I (-2.4 fold) but not by either treatment alone. MyF5 was found to be down regulated in response to rIL-1β (-7.8 fold) and this effect was not significantly altered by co-incubation (-10.7 fold) (Figure 3). Lastly, atrogin-1 was found to be significantly down regulated in response to stimulation with rIGF-I (-4.9 fold) but unaltered by rIL-1β treatment (Figure 3). Co-incubation with rIL-1β + rIGF-I however ablated the rIGF-I effect.

**Figure 4:**
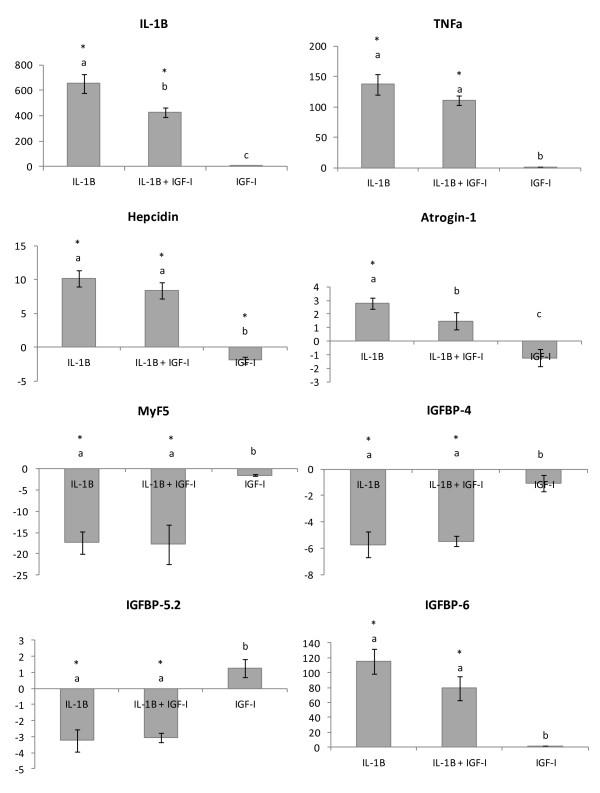
**Fold change of genes involved in both the immune response and growth in response to 24 h stimulation with either rIL-1β (25 ng/ml), rIL-1β (25 ng/ml) + rIGF(100nM), or rIGF(100nM).** * represents a significant difference from control, bars which share a letter are not significantly different. All fold changes were calculated compared to unstimulated control samples. Comparative gene expression was measured with qPCR.

At 24 h co-stimulation of cells with rIL-1β + rIGF-I significantly reduced the expression of IL-1β relative to cells only stimulated with rIL-1β, from 654 fold to 427 fold (Figure 4). No significant effect of co-incubation of rIL-1β + rIGF-I was found on the expression of TNFα or hepcidin (Figure 4). Additionally co-incubation did not alter the expression of MyF5 or any of the IGFBPs. While rIL-1β alone significantly increased the expression of atrogin-1 (2.8 fold) this increase was not found in cells co-incubated with rIL-1β + rIGF-I (Figure 4). However the co-incubated cells had significantly increased expression of atrogin-1 compared to cells stimulated with just rIGF-I. rIGF-I alone also significantly reduced the expression of hepcidin (-1.9 fold) but had no effect on the other genes. All the genes tested that were also hybridised with sufficient intensity on the microarray showed the same direction and similar magnitude of response in this cell culture experiment.

## Discussion

Regulation of muscle mass is under the control of a multitude of regulators related to both anabolic and catabolic processes. We hypothesised that the muscle cells would respond to the inflammatory stimulus by signalling the induction of inflammatory responsive genes in addition to other pathways related to protein metabolism for release of free amino acids as occurs during the acute phase response [56], or for gluconeogenesis and energy reallocation. Our approach of using primary cells to examine the transcriptomic responses of muscle cells stimulated with IL-1β avoids complex host and cell type responses observed during *in vivo* experiments. The response to the recombinant cytokine resulted in a large panel of genes that were significantly modulated being both increased and decreased in expression. Using gene ontology enrichment analysis for biological processes five key enriched processes were revealed: immune function, protein catabolism, IGF and growth regulation, cell cycle and lipid metabolism.

### Immune response

The immune genes up regulated included several proinflammatory cytokines such as TNF-α, IL-1β and IL-8, indicating that stimulated myocytes are capable of synthesising these cytokines and are undergoing a proinflammatory response. The response to IL-1β is extremely rapid in other cell types in fish [57,58] and it is likely that within 24 h these molecules will have been secreted into the medium. Several genes in the inflammatory signalling cascade were induced including NFκB subunits p100 and p105, and the NFκb inhibitor (IκB), as seen during inflammation in other cell types [58]. Under normal conditions IκB binds to NFκB to inactivate it but IκB is phosphorylated by IκB kinase (IKK) and subsequently ubiquitinated and destroyed by the proteasome [59,60]. A related key signalling molecule up regulated was MAP kinase-interacting serine/threonine kinase 2, central to the MAPK pathways involved in IL-1β signalling [61], and with additional roles in the regulation of IGF signalling [26]. Another important transcription factor up regulated was the MAPK activated jun-B which increases transcription of IL-1β responsive genes generally at AP-1 responsive sites [62]. Interestingly, although jun-B may be associated with inflammatory signalling, it also has a role in maintaining muscle mass and its over expression in mammals can induce hypertrophy [63], indicting complex regulation of transcriptional machinery. In parallel to this, several genes encoding proteins that have roles as anti-inflammatory factors were activated; these include two suppressors of cytokine signalling (SOCS 1 and 3), IL-10 and an IL-10 receptor chain. SOCS proteins are often co-regulated during inflammation to prevent cellular damage and are negative regulators of cytokine signalling and function that interferes with signal transduction from cytokine receptors. The SOCS genes have been characterised in salmonid fish [64] and are increased in expression following stimulation with several different cytokines including IL-1β, TNFα and IL-6. Other immune related genes such as hepcidin, ferritin, C type lectin and the complement system were also significantly increased in expression. Both hepcidin and ferritin control iron availability and have antimicrobial actions with ferritin sequestering iron to reduce availability to microbes [65], whereas hepcidin also has direct antimicrobial properties and is often described as an antimicrobial peptide [66-68]. C-type lectins recognise carbohydrate moieties and are often induced by proinflammatory signals [58,69], to regulate a variety of immune processes including the complement system [70-72]. There was also activation of some genes that are components of the adaptive immune system, such as major histocompatibility complex (MHC) class I and CD4-like protein, but at the time point we examined the predominant immune gene response was by molecules of the innate defences.

### Protein catabolic processes

A major proteolytic pathway in muscle is the ubiquitin proteasome pathway, which in mammals is believed to be responsible for the majority of muscle protein degradation initiated by a number of different stimuli including inflammation in mammals [30]. This pathway has also been seen to be activated in salmonid fish during muscle atrophy induced by food deprivation [45,73,74], hormonal changes [75], with some evidence of several components being modulated during immune responses [45,76]. The end product of proteolysis is the release of free amino acids for *de novo* protein synthesis or for the oxidation of the amino acids and gluconeogenesis. Following the inflammatory stimulus, several components of the UBP were increased in expression in myocytes. Several ubiquitin E3 ligases, which initiate the targeting of proteins for degradation and a number of proteasome subunits from the catalytic core of the proteasome (β subunits 6, 7 and 8 and α subunit 6), were increased in expression. We hypothesise that these changes would result in increased protein degradation and reduced muscle growth releasing free amino acids, which *in vivo* would be reallocated to other organs, such as the liver as occurs in mammals [77,78]. Although the predominant proteolytic genes modulated were related to the UBP system, cystatin B, an inhibitor of the acidic lysosomal cathepsins was down regulated, possibly indicating an increase in cathepsin bioavailability and activity [79]. In addition the calcium dependant protease calpain subunit 1 was down regulated following the IL-1β stimulation. This protease has roles in positive regulation of myofusion inhibiting the differentiation of myocyte cells [80,81] and this may indicate a reduction of muscle cell differentiation.

Other proteases observed to be increased included collagenase 3, that is increased in expression in NFkB mediated inflammation in mammals [82-84] and during vitellogenesis induced muscle atrophy in salmonids [43]. Angiotensinogen, the precursor of both angiotensin I & II, was also increased in expression, and is known to interfere with the actions and production of IGF-I, which in mammals is mediated by the NFκB pathway in collaboration with protein kinase C [85,86].

In general there was a clear effect of rIL-1β on the expression of genes related to catabolism as evidenced by a transcriptomic shift towards muscle catabolism by the increase in mRNAs related to protein degradation and the down regulation of protein degradation related genes that have positive effects on growth.

### IGFBPs

The IGF system is instrumental in the control of protein synthesis and growth in both mammals and fish [87]. The activity of IGF is under tight control, often by a family of IGF binding proteins (IGFBPs), which have recently been characterised in salmonid fish [88]. They function by either stabilising the IGF or by competitively binding the IGF to prevent attachment to the IGF receptor [87] and thus reducing the anabolic effects of IGF on the cells.

We found several IGFBP encoding mRNAs were modulated by the proinflammatory stimulus. IGFBP-6 is thought to have a binding preference for IGF-II but also binds IGF-I [89]. These direct effects on the activity of both IGFs might drive the cells away from high levels of protein synthesis and anabolism towards a state of catabolism [90,91]. Previous studies indicate IGFBP-6 expression is associated with the inhibition of cell proliferation in both fish [12] and mammals [89,92]. Additionally IGFBP-6 expression is reduced during resumption of growth following starvation [20,93]. These findings tend to indicate that IGFBP-6 expression has a negative relationship with growth due to the ability of IGFBP-6 to act as a negative regulator of IGF-I & II activity, thus making an increase in the expression of IGFBP-6 a potential marker of inflammation induced catabolism in salmon muscle.

Other IGFBPs 4, 5 and rP1 were all decreased in expression following the inflammatory stimulus. In salmonids IGFBP-4 expression in muscle is increased by anabolic stimuli such as refeeding after starvation [20,93] and is positively related to the expression of the promyogenic transcription factors MyoD and MyF5 *in vitro*[12]. IGFBP-5 can potentiate the effects of IGF-I especially with regard to bone [94] and muscle differentiation [90]. In rainbow trout IGFBP-5 increased in expression in muscle during refeeding after starvation [93] and, in Atlantic salmon primary myocytes, the expression of IGFBP-5 decreased during cell proliferation suggesting this protein is associated with entry to cell cycle [12]. Together these results suggest the IGFBPs are responding in a coordinated fashion to reduce IGF signalling and altering the balance between anabolic and catabolic pathways.

### Growth regulation and structural proteins

Many transcription factors involved in growth regulation were altered. CCAAT/enhancer binding protein delta was increased, and is a transcription factor with multiple functions, that is positively related to myostatin expression in mammals [95]. In rainbow trout muscle it is increased during energy reallocation caused by vitellogenesis [43] indicating a blocking of muscle growth. A second key transcription factor, NFκB, is often associated solely with immune function but also negatively regulates myogenesis via the transcriptional repressor YY1 [34,96]. Both of these molecules were increased in this experiment by IL-1β. YY1 is likely to be a mediator of NFκB induced muscle growth inhibition, achieving this by silencing myofibrillar promoters in myoblasts [34,96]. MyF5, a muscle specific transcription factor, regulates muscle cell differentiation [3,97] and a reduction in its expression level in this experiment fits with our anticipated reduction of muscle growth markers in response to rIL-1β stimulation. Additionally we found a general decrease in expression of mRNAs coding for muscle structural proteins such as collagens, myosins, actin and keratin, consistent with the hypothesis that the muscle cells are undergoing a reduction in growth in response to immune stimulation, as previously shown in mammals [98].

### Cell cycle

The cell cycle is largely mediated through the actions of cyclin/cyclin dependent kinase complexes [99]. In the salmon myocytes multiple cyclins were modulated by IL-1β stimulation strongly suggesting cell cycle activity is being altered. For example, cyclin D1 expression was increased and functions in combination with cyclin dependent kinases to initiate and progress through the G1 phase of the cell cycle [99,100]. The increase of cyclin D1 may be related to NFκB mediated arrest of muscle growth by preventing myocyte differentiation [101]. Cyclin G2 was also increased and may inhibit entry into the cell cycle [102,103].

The remaining cyclins A2, B (1 and 2) and E (1 and 2) were all decreased in expression. Cyclin A2 is a rate limiting factor during S-phase and DNA synthesis and entry to mitosis [104], whereas cyclins E1 and E2 are responsible for the transition from G1 to S phases and initiation of DNA replication [105]. Cyclins B1 & B2 have roles during the S-phase and the M-phase and are crucial for maintenance of the mitotic state [106]. Several other cyclin related kinases, cell division proteins and minichromosome maintenance complex components were generally down regulated indicating a major reduction in cell cycle activity and DNA metabolism in these primary muscle cells under an inflammatory stimulus.

### Lipid and sterol metabolism

A final group of genes found to be altered were those related to lipid and sterol metabolism, here several cholesterol transport proteins were increased in expression including Apo L3, glycolipid transfer protein and lipoprotein lipase. Apo-L3 is known to be a TNF-α inducible protein and its expression is known to be involved in the activation of the NFκB signalling pathway activated by cytokines in mammals [107]. The increase in the lipoprotein lipase could reflect an increased breakdown of lipoproteins for immune or cellular processes; this gene is under the control of many different signals in mammals including insulin, nutritional state and cytokines [108]. Prostaglandin-endoperoxide synthase 2, a gene known in mammals to be inducible by a variety of inflammatory substances [109], was also increased as a result of the rIL-1β stimulation. Apo A1 binding protein and Apo B were reduced in expression as well as several other sterol synthesis proteins. These results indicate that lipid metabolism is being actively changed in these myocytes under the inflammatory stimulus, resulting in complex changes in transcription of their mRNAs. Many of these changes could be mediated through intracellular crosstalk with the IGF/insulin pathway(s) [26,110].

### Interaction between IGF-I and IL-1β

Results from the microarray clearly indicated genes involved in the IGF regulation were being modulated by IL-1β, especially a number IGF binding proteins. We performed additional experiments to address if transcripts altered by inflammation would be modulated by IGF-1, a hormone which drives cells towards an anabolic status. Atrogin -1 a key gene involved in protein degradation was reduced in expression by incubation of cells with rIGF-1 as previously reported, conversely it is increased following incubation with IL-1β as occurs in mammals [31]. When cells were co-incubation with rIL-1β and rIGF-I an almost total inhibition of the atrogin-1 down regulation was found, suggesting the proinflammatory signal is blocking the anabolic effect of IGF-1. IL-1β alone results in an increase in atrogin -1 expression at 24 h as found in mammalian cells stimulated by proinflammatory cytokines [111]. The co-stimulation also decreased the magnitude of the response of the antimicrobial peptide hepcidin, highlighting an alternative allocation of resources depending on the signalling the muscle cells are receiving. Together these results show how anabolic signals may attenuate transcription of immune defence molecules and that proinflammatory signals can increase catabolic effects in the cells.

## Conclusions

**Figure 5:**
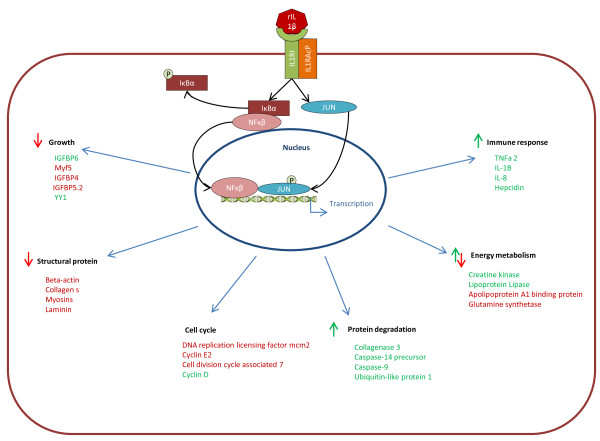
**Model of the proposed actions of rIL-1β on gene expression and physiology of muscle cells *in vitro*.** rIL-1β is recognised by its receptor and an accessory protein, this then sets off a signal cascade resulting in the activation of NFκβ and JUN which in turn result in changes in gene expression and physiology. The proposed changes in physiology are shown in black. Green arrows show a positive effect on this aspect of physiology and red arrows show a negative effect. A sample of genes related to each aspect of physiology is shown in either red (up regulation) or green (down regulation).

Muscle tissue is a complex and dynamic organ and is generally the only protein storage organ in the body; hence it needs to be able to control the synthesis of proteins and release of amino acids via degradation under a variety of environmental and physiological conditions. Muscle does respond to immune insults in fish, [45,76] but to date these responses have not been examined in an *in vitro* system removing the mileu of cytokines and hormones. Here we show a direct effect of a proinflammatory cytokine on primary muscle cells that induces not only immune genes, but also alters the wider transcriptome indicating increased catabolism, lipid mobilization and decreased cell proliferation with a large role potentially for the IGFBPs (Figure 5). Subsequent experiments demonstrate that both IL-1β and IGF-1 exert disparate effects on mechanisms that regulate growth and other physiological responses as highlighted by their interaction of expression on atrogin-1. These findings will direct future research into the control of muscle mass in ectothermic animals, particularly in relation to health and nutrition.

## Methods

### Myosatellite isolation and stimulation

Atlantic salmon (mean weight of 25 g and mean length of 12 cm) were used for skeletal muscle myosatellite cell extraction, as previously described [112-114]. For each muscle extraction 6 fish were used (~1.5 g tissue from each fish), this was to remove any individual fish effects. No experimental procedures were carried out on the fish and fish maintenance was in line with national ethical guidelines in an experimental facility at University of Aberdeen, UK. Fish were maintained in freshwater and fed a commercial diet at 1.5% body weight per day. Fish were killed using a schedule one method and muscle tissue from above the mid line of the fish was removed sterilely with scalpel and forceps. This pooled muscle (approx 9 g) was placed into a pre-weighed flask containing 30 ml of Leibovitz L15 medium (Gibco) + penicillin/streptomycin 1% (Pen/Strep, Gibco, Penicillin 10,000 units/ml, streptomycin 10,000 μg/ml) (L15 + P/S). The muscle was diced into small blocks (2 mm^3^) using sterile procedures and the diced muscle then centrifuged (300 g, 5 min) and the supernatant removed. The tissue was digested in collagenase (0.2%) at 11°C for 1 h. Following digestion the cell suspension was centrifuged (300 g, 5 min) and washed before being centrifuged again (300 g, 5 min). This pellet was digested in trypsin (0.1%) at 11°C for 30 min. The cell suspension was again centrifuged (300 g, 40 sec) and the remaining supernatant was added to L15 + P/S plus 10% foetal calf serum (FCS, Sigma) before being passed through a 200 μm nylon mesh. The suspension was centrifuged (300 g, 15 min), the supernatant was removed and 12 ml of L15 + P/S + 10% FCS were added. Finally the contents of this tube were added to two 6 well plates. Prior to plating laminin (mouse laminin, Sigma-Aldrich) was applied to the well surfaces 24 h before the cells were plated out, at a concentration of 1 mg/ml. Cell cultures were then left for 1 h for microsatellite cells to bind to the surface before the medium was first changed and cells allowed to differentiate at 22°C, with the medium being changed every 2 days.

### Stimulation of cells

Cells were cultured for 4 days to allow cellular differentiation, this was observed using light microscopy. Morphology typical of satellite cells and time taken to reach confluence in our system was approximately 6 days. The cells were found to exhibit the typical growth pattern previously observed for muscle satellite cells as described in Bower & Johnston (2010) [12]. Initially cells were mono-nucleic before beginning to proliferate and differentiate into spindle shaped cells, a small number of which were beginning to fuse together by day 4. For the microarray experiment stimulations, the medium was removed and 1 ml of new medium (with 0.5% FCS) containing either 10 μl recombinant trout IL-1β protein (rIL-1β) to achieve a concentration of 25 ng/ml or a non-stimulated control with 10 μl PBS. The concentration of IL-1β has previously been determined to be optimal in salmonid cell lines [58]. The cells were then stimulated for 24 h before RNA extraction was carried out.

Subsequent experiments were carried out to further investigate the effects of rIL-1β at different time points and to investigating the effects of the anabolic hormone IGF-I on rIL-1β actions. For these experiments the same procedure was carried out with the only alterations being the length of time the cells were stimulated and in some cases the addition of 100nM of recombinant human IGF-I (rIGF-I) (Sigma). For these experiments the cells were cultured in the stimulant for either 6, 24 or 48 h as specified.

### Study design and sample replicates

**Figure 6:**
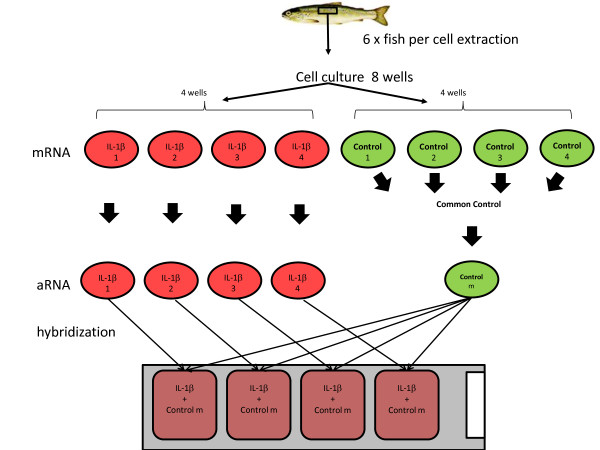
**The experimental design carried out for the microarray experiment.** Cells were genereated from six individual fish, these cells were pooled and plated onto six well plates. RNA extracted from four biological replicates stimulated with rIL-1β stimulation were kept separate. For control wells, RNA was pooled to generate a common reference RNA sample.

Cells cultures were generated from six individual fish, this allowed purification of sufficient cells for two six well plates. For the microarray four of these wells were used as biological replicates and stimulated with rIL1β and the remaining four were mock stimulated as described above. RNA extractions were performed and the stimulated samples were kept separate whereas the control unstimulated samples were pooled to have a single common reference prior to mRNA amplification and labelling (Figure 6).

### RNA extraction

For microarray experiments RNA was isolated using the RNAeasy extraction kit (Qiagen) as per the manufacturer’s instructions. For all other samples RNA was isolated using Trizol (Sigma) as per the manufacturer’s instructions. In both cases the RNA was resuspended in 50 μl of nuclease free water and concentration measured by Nanodrop ND1000 (LabTech). The quality of the RNA was assessed using an Agilent Bioanalyzer RNA 6000 Nano Kit as per the manufacturer’s instructions.

### Microarray hybridization and analysis

Microarray analysis of the samples was carried out using a custom-designed Agilent microarray platform with 4 × 44 K probes per slide (Salar_2; Agilent Design ID:025520) as previously described [115]. The microarray platform design is available at array express accession number A-MEXP-2065.

To produce labelled template for hybridisations aRNA was generated using a MessageAMP II aRNA Amplification Kit (Ambion) as per the manufacturer’s instructions. Briefly 1 μg of total RNA was reverse transcribed to create first strand cDNA. This was then used in the synthesis of second strand cDNA and this product was purified using the supplied reagents and columns. Finally the *in vitro* transcription to synthesise amino allyl modified aRNA was carried out to incorporate amino allyl dUTP in to the aRNA after a 14 h incubation at 37°C and the product purified using the supplied reagents and columns.

For incorporation of flouresence dye 3 μg of aRNA in a volume of 10 μl was denatured at 70°C for 2 min, and to this was added 3 μl of NaHCO_3_ and 2 μl Cy dye (Cy3 or Cy5 mono-reactive dye pack, Amersham, resuspended in DMSO). The dye was allowed to incorporate for 1 h in the dark before excess dye was removed using a DyeEx spin column purification kit (Qiagen). Confirmation of dye incorporation and aRNA recovery was by nanodrop spectrometry. Agilent microarrays were hybridised according to the manufacturer’s instructions as described by [115]. Briefly 825 ng cDNA of each labelled template was fragmented in the dark and made up to a final volume of 20 μl with nuclease free dH_2_O. After fragmentation, 57 μl of 2XGEx hybridisation buffer (Agilent) was added to each sample which was then briefly mixed and spun down before being stored on ice in preparation for loading 103 μl onto each microarray. Four biological replicates of rIL-1β stimulated cells aRNA were labelled with Cy3 dye and a control consisting of four biological replicates of control cells aRNA was labelled with Cy5 dye. Each rIL-1β stimulated aRNA was hybridised against the control. Hybridisations were carried out in a microarray hybridisation oven (Agilent) overnight (18 h) at 65°C. Following hybridisation the slides were washed in the gene expression wash buffers 1 and 2 (Agilent) following the manufacturer’s instructions. The slides were scanned using a GenePix personal 4100A scanner (axon Instruments) at a resolution of 5 μm. Files saved as ^*^. TIF files were extracted using feature extraction software v9.5.3 (Agilent) and background correction and normalization were carried out within this program. Statistical analysis was performed using the Genespring GX analysis platform (version 9.5, Agilent Technologies). Significant differences between IL-1β stimulated cells and control cells were established by t-test analysis (P < 0.05) followed by correction for multiple tests (Benjamini Hochberg FDR post hoc test). Further filtering was carried out to maintain only those genes that showed a ≥2 fold difference in expression as a result of the stimulation. The raw hybridisations data have been deposited at ArrayExpress under accession number (http://www.ebi.ac.uk/arrayexpres, E-MTAB-1692).

Gene ontology (GO) enrichment was carried out on all features with associated GO identifiers using GOEAST software [116]. Fishers exact test was used within the GOEAST program to determine if the GO identifiers occurred significantly more often in a group than would be expected by chance. The output from GOEAST was entered into the software REVIGO [117] to remove redundant GOs.

### Gene expression analysis by real time PCR

For cDNA synthesis total RNA (500 ng) was added to 1 μl of oligo-dT_17_ (500 ng μl^-1^) and RNase/DNase free water (Sigma-Aldrich) up to a volume of 11 μl, then denatured for 3 min at 70°C and cooled on ice. To each of these denatured RNA samples was added 1 μl of Bioscript reverse transcriptase enzyme (10000U μl, Bioline), 5 μl of 5x reaction buffer, 1 μl of deoxynucleoside triphosphate mix (12.5 mM each, Bioline) and 7 μl of RNase/DNase free water (Sigma-Aldrich) and the mix incubated at 42°C for 1.5 h in a final volume of 25 μl. The cDNA was diluted 4-fold to 100 μl in 1x TE. (Sigma-Aldrich). qPCR amplifications were performed using 3 μl cDNA, 2x Sybr Green PCR master mix (Biorad) and gene specific primers (Table 7) at 10 μM, with a final reaction volume of 20 μl in 96 well plates in an Opticon real time PCR machine. A typical qPCR cycle used was an initial denaturation for 5 min at 95°C followed by 30-40 cycles of 30 sec at 94°C, 30 sec at 55°C, 30 sec at 72°C, and a final 5 sec at 80°C in which the machine read the plate. The number of cycles used was varied depending upon the expression level of the gene under study. The annealing and measuring temperature was also varied depending upon the primers being used. To calculate the relative quantities of the gene of interest in each sample the standard curve method of relative quantification was used. A dilution series of cDNA diluted 1, 10, 100 and 1000 times was run in each plate to provide a standard curve which was used to calculate primer efficiency to ensure efficiency between 1.8 and 2. Next a linear regression was applied to the standard curve with the subsequent formulas being used to interpolate the relative amount of the gene of interest in the samples [118]. Negative control PCRs were run on all plates. For normalization three “house keeping genes” previously found to be stable during immune reactions in fish, namely elongation factor 1α, hypoxanthine-guanine phosphoribosyl transferase (HPRT1) and RNA polymerase 1 (RPL1) were used. The arbitrary units of each individual house keeping gene were normalized to give an average value of 100 to account for different expression levels of the genes, a geometric mean of the arbiatry units of the three housekeeping genes was taken and used for normalization of genes of interest. None of these three genes were found to show any difference in expression over the experiment. For the comparison between microarray expression and qPCR one way T-tests were used to establish if a difference between stimulated and control samples was significant at P ≤ 0.05. For the subsequent qPCR experiments significant differences were established using one way ANOVAs with a Fishers post hoc test to control for multiple testing. Statistics were performed on log transformed arbitrary units. Fold was calculated by division of experimental sample arbitrary units by the average of the control. In the case of negative fold changes below 1, the number was inverted to give a negative fold change.

**Table 7:** Primers used for qPCR

**Name**^**1**^	**Accession**^**2**^		**Sequence 5′ to 3′**	**bp**^**3**^
EF-1α	AF498320.1	F	CAAGGATATCCGTCGTGGCA	327
		R	ACAGCGAAACGACCAAGAGG	
RPL1	CB516726	F	ACTATGGCTGTCGAGAAGGTGCT	118
		R	TGTACTCGAACAGTCGTGGGTCA	
HPRT1	EG866745	F	CCGCCTCAAGAGCTACTGTAAT	255
		R	GTCTGGAACCTCAAACCCTATG	
IL-1β	AJ223954.1	F	GCTGGAGAGTGCTGTGGAAGAACATATAG	179
		R	CCTGGAGCATCATGGCGTG	
Hepcidin	NM_001140849.1	F	CATTGAAAATCGTGCATTGG	150
		R	AGGCCTTCATTCTCGGTTTT	
IGFBP-6	DQ190459.2	F	GCTCAATAGTGTTCTGCGTGG	118
		R	CTTGGAGGAACGACACTGCTT	
TNFα1	NM_001123589.1	F	TGTGTGGCGTCCTCTTAGTAGCAGCTT	101
		R	CTCCATTTTGTCCTGCATCGTTGC	
IGFBP-4	EU861007	F	TGTCGTGCTGAGCTGCAGAG	129
		R	TGGCTGGCACTGCTTGGCAT	
IGFBP-5.2	EU861009	F	TTCTCCAGAGGAAGCTATGTTAG	170
		R	TCAAGGCTGCTGACAGAGTG	
Myf5	TC97553	F	CGCATACCGCTTTTACTTCC	245
		R	TGATCATGAGAAACGTGAAGC	
High choriolytic enzyme	TC63579	F	ATCAATGGGGCTCATCTCAG	239
		R	ATGAGCAAACACGCAGTGAC	
Atrogin-1	GU456729.1	F	CGAGTGCTTCCAGGAGAATCTG	384
		R	GTCTGAAGGAGCTCCTTGATGG	

List of primers used in qPCR, all primers were checked to ensure that they had an efficiency of between 1.8 & 2.0. All except for Atrogin 1 were used for qPCR confirmation of the microarray.^1^Identity of the gene, ^2^ Accession number of the cDNA sequence, ^3^Size of PCR product produced by the primers.

### Availability of supporting data

The microarray data is submitted to Array express public archive (E-MTAB-1692). Other supporting data is as supplementary files attached to this paper.

## Competing interests

The authors do not have any competing interests.

## Authors’ contributions

NJP performed the cell culture stimulations, microarray data analysis, real time PCR and wrote the manuscript. LT carried out the microarray hybridizations CJS and SAMM conceived and designed the experiment and drafted the manuscript. All the authors read and approved the final manuscript.

## Supplementary Material

Additional file 1: Table S1 The genes shown were significant at p < 0.05 following t- tests & Benjamini–Hochberg FDR and greater than 2-fold change. ^1^Indicates the unique code for the feature on the microarray, ^2^Accession number of the cDNA sequence. ^3^Fold-change for genes higher or lower expressed in cells stimulated with rIL-1β compared to control. ^4^Regulation of fold change. ^5^Identity of the probe target as determined by BLASTX & BLASTN search. Click here for file

Additional file 2: Figure S1 Scatter plot showing comparative expression of genes from the microarray (n = 4) and qPCR (n = 4). The mean value was used in situations where a gene appeared multiple times on the microarray. Regression analysis found the expression levels for these 8 genes were significantly correlated between microarray and qPCR (p = 0.001). Click here for file
