# Supplementary material for: Inflammatory responses in primary muscle cell cultures in Atlantic salmon (Salmo salar)
Source: BMC Genomics. 2013 Nov 1;14:747. doi: 10.1186/1471-2164-14-747 (PMC3819742; doi:10.1186/1471-2164-14-747)
Supplement: Additional file 1: Table S1 — The genes shown were significant at p < 0.05 following t- tests & Benjamini–Hochberg FDR and greater than 2-fold change. 1Indicates the unique code for the feature on the microarray, 2Accession number of the cDNA sequence. 3Fold-change for genes higher or lower expressed in cells stimulated with rIL-1β compared to control. 4Regulation of fold change. 5Identity of the probe target as determined by BLASTX & BLASTN search. [file 1471-2164-14-747-S1.pdf]

Supplementary Table 1

The genes shown were significant at p<0.05 following t- tests & Benjamini–Hochberg FDR and greater than 2-fold change. [1]Indicates the unique code for the feature on the microarray, [2]Accession number of the cDNA sequence. [3]Fold-change for genes higher or lower expressed in cells stimulated with rIL-1β compared to control [4]Regulation of fold change [5]Identity of the probe target as determined by BLASTX & BLASTN search.

[1] ProbeName	[2] Acc	[3] FC	[4] Regulatic	[5] Identity
Ssa#S24188435	AY929386	216.3	up	tumor necrosis factor alpha-2 precursor
Omy#gi185133433	NM_001124347	197.9	up	Oncorhynchus mykiss interleukin-1-beta (LOC100136024), mRNA
Ssa#KSS3969	KSS3969	158.1	up	Leukocyte cell-derived chemotaxin 2 precursor
Ssa#S23659093	S23659093	99.4	up	Angiotensinogen
Ssa#STIR00083_4	AY929385	99.2	up	tumor necrosis factor alpha
Omy#gi13235345	AJ279069	93.3	up	Oncorhynchus mykiss mRNA for putative interleukin 8 (IL-8 gene)
Omy#gi31087935	gi31087935	72.9	up	Oncorhynchus mykiss interleukin-8 (IL-8) gene, IL-8-E allele,
Ssa#S32001918	S32001918	71.7	up	Small inducible cytokine A4 precursor
Ssa#STIR00083_2	AY929385	71.0	up	tumor necrosis factor alpha
Omy#gi31087937	gi31087937	69.5	up	Oncorhynchus mykiss interleukin-8 (IL-8) gene, IL-8-nL allele,
Omy#gi31087933	gi31087933	60.7	up	Oncorhynchus mykiss interleukin-8 (IL-8) gene, IL-8-D allele,
Ssa#S22336501	S22336501	58.0	up	toll-like leucine-rich repeat
Ssa#S31995868	S31995868	49.9	up	transposase
Ssa#STIR00013_3	AY929386	47.0	up	tumor necrosis factor alpha
Ssa#STIR19180	TC79788	46.9	up	Salmo salar clone ssal-rgf-538-144 Transmembrane protein 100 putative mRNA,
Ssa#STIR22719	TC85155	41.4	up	mantle gene 8
Ssa#S35697234	S35697234	41.1	up	P-selectin precursor
Omy#gi31087931	gi31087931	40.4	up	Oncorhynchus mykiss interleukin-8 (IL-8) gene, IL-8-C allele,
Ssa#S41489473	S41489473	36.7	up	CCL4-like chemokine
Ssa#S30240261	S30240261	36.2	up	Cholesterol 25-hydroxylase-like protein A
Ssa#S31995868_S	S31995868	35.5	up	transposase
Ssa#STIR00162_4	gi|209154423	33.9	up	transferrin
Ssa#S18892517	S18892517	31.8	up	transferrin
Con_CANDS_14	gi|209154423|gb|BT045	30.6	up	423|gb|BT045182.1| Salmo salar clone ssal-rgf-514-133 Serotransferrin-1 precursor putative mRNA,
Ssa#S31981965	S31981965	30.4	up	glutathione peroxidase 3 (plasma)
Ssa#STIR00162_2	gi|209154423	30.2	up	transferrin
Ssa#STIR21056	TC82634	30.0	up	---NA---
Ssa#S34822137	AM397592	29.7	up	complement component C3
Ssa#STIR14630	TC73147	28.9	up	Gasterosteus aculeatus clone CH213-10I3, complete sequence
Ssa#CL122Ctg1	CL122Contig1	28.5	up	interleukin-1 beta
Ssa#S20293825	S20293825	28.1	up	interleukin-1 beta
Ssa#STIR00010_4	AY617117	27.6	up	interleukin-1 beta
Ssa#S30240738_S	S30240738	26.3	up	complement c1q-like protein 4 precursor
Ssa#S18835882	S18835882	26.0	up	si:ch1073-126c3.2
Ssa#STIR04816	BT047247	25.5	up	hepcidin
Ssa#S18836142	S18836142	25.1	up	Microfibril-associated glycoprotein 4
Ssa#S30240738	S30240738	25.0	up	tetraodon nigroviridis full-length cdna
Ssa#S35545113	S35545113	23.8	up	hypothetical protein LOC560193
Ssa#S18890165	CB515874	23.2	up	Creatine kinase, ubiquitous mitochondrial precursor
Ssa#STIR05988	gi|209156015	23.2	up	transmembrane protein 100
Ssa#S35595553	S35595553	23.0	up	AF401631_1TNF decoy receptor
Ssa#STIR10460	TC67326	23.0	up	novel proteinoptineurin (zgc:77868)
Ssa#S35478597	S35478597	22.6	up	Regulator of G-protein signaling 5
Ssa#STIR15959	TC75013	22.1	up	Zebrafish DNA sequence from clone DKEY-63L2 in linkage group 10, complete sequence
Ssa#KSSb2780	KSSb2780	21.4	up	Matrix metalloproteinase-9
Ssa#TC102550_S	TC102550	21.4	up	unnamed protein product
Ssa#STIR24426	TC87710	20.9	up	hepcidin
Omy#S37211068	EF175381	20.7	up	prostaglandin G/H synthase 2b
Omy#S34424325	S34424325	19.8	up	alpha-2,3-sialyltransferase ST3Gal I
Ssa#S31993512	S31993512	19.6	up	Hepatocyte growth factor activator
Ssa#STIR14407	TC72828	18.9	up	Zebrafish DNA sequence from clone CH211-222M15 in linkage group 15, complete sequence
Ssa#STIR03218	gi|209735567	18.6	up	hemagglutinin amebocyte aggregation factor precursor
Omy#S22610346	S22610346	18.6	up	interleukin-11
Omy#S24637060	S24637060	18.4	up	---NA---
Con_CANDS_12	gi|209736931|gb|BT049	18.1	up	931|gb|BT049534.1| Salmo salar clone ssal-evd-517-045 Hepcidin-1 precursor putative mRNA,
Ssa#STIR08688	TC65065	17.9	up	Salmo salar clone ssal-rgb2-578-320 Ferritin, lower subunit putative mRNA,
Ssa#S31979620	S31979620	17.3	up	glutathione peroxidase 3 (plasma)
Ssa#S30261281	DW548249	17.2	up	IGF binding protein 6
Ssa#STIR00013_4	AY929386	17.1	up	tumor necrosis factor alpha
Omy#TC167537	TC167537	17.0	up	homo sapiens protein tyrosine non-receptor type 3 transcript variant mrna
Ssa#S21512941	AY572832	16.1	up	C type lectin receptor A
Ssa#STIR22988	TC85560	16.1	up	complement component C3
Ssa#STIR10673	TC67607	15.4	up	chemokine (c-x-c motif) receptor 3
Ssa#STIR05999	gi|209155993	15.3	up	chemokine (c-x-c motif) receptor 3
Ssa#STIR17909	TC77896	14.9	up	---NA---
Ssa#TC110050	TC110050	14.4	up	Metalloreductase STEAP4
Ssa#STIR17200	TC76867	13.9	up	tumor necrosisalpha-induced protein 2
Ssa#STIR35259	NM_001124618	13.8	up	complement protein component C7-1
Ssa#KSSb2228	KSSb2228	13.8	up	toll-like leucine-rich repeat
Ssa#S48434567	S48434567	13.8	up	interleukin 8
Ssa#S21512940	S21512940	13.6	up	C-type lectin domain family 4 member E
Ssa#STIR00012_4	AY848944	13.3	up	prostaglandin-endoperoxide synthase 2 (prostaglandin g h synthase and cyclooxygenase)
Ssa#STIR23928	NM_001124410.1	13.3	up	Oncorhynchus mykiss mRNA for complement factor H precursor (cfh gene)
Ssa#STIR00019_2	BQ036596	13.0	up	complement component 7
Ssa#STIR00160_3	gi|209736931	13.0	up	hepcidin
Ssa#S35561448	S35561448	12.8	up	Catechol-O-methyltransferase domain-containing protein 1
Ssa#S35568346	S35568346	12.6	up	alpha 2,6-sialyltransferase ST6GalNAc I
Ssa#TC102550	TC102550	12.5	up	salmo salar clone bac chori214- complete sequence
Ssa#STIR23807	TC86795	12.3	up	Salmo salar clone ssal-rgf-507-188 unknown large open reading frame mRNA, novel cds
Ssa#S35595553_S	S35595553	12.2	up	AF401631_1TNF decoy receptor
Ssa#S30260858	S30260858	12.2	up	similar to Rasip1 protein
Ssa#STIR23716	TC86664	12.2	up	---NA---
Ssa#S32009767	S32009767	12.1	up	salmo salar clone ssal-rgf-001-138 multimerin-2 precursor pseudogene cds
Ssa#STIR34850	TC103455	12.0	up	Serine/threonine-protein kinase Pim-1
Omy#S34425780	S34425780	11.8	up	neprilysin
Ssa#S22839335	S22839335	11.6	up	TNF-alpha 2

Ssa#KSS4972	KSS4972	11.6	up	danio rerio zgc: mrna (cdna clone mgc:191676 image:100059985)
Ssa#STIR00019_3	BQ036596	11.4	up	complement component 7
Ssa#STIR21179	TC82828	11.4	up	hig1 domainmember 2a
Ssa#STIR19531	TC80331	11.2	up	glutathione peroxidase 3
Ssa#S35672975	S35672975	11.2	up	similar to tripartite motif-containing 39
Ssa#STIR15643	TC74562	11.1	up	scavenger receptor classmember 2
Ssa#S31979620_S	S31979620	11.1	up	glutathione peroxidase 3 (plasma)
Ssa#STIR26053	TC90164	11.1	up	g protein-coupled receptord17leh66c region
Ssa#STIR09097	TC65585	11.0	up	---NA---
Ssa#S31983951	S31983951	10.9	up	deltaB
Omy#S15239605	S15239605	10.8	up	3'-phosphoadenosine 5'-phosphosulfate synthase 2
Ssa#S35559348	S35559348	10.8	up	Nicotinic acid receptor 1
Ssa#DY740104	DY740104	10.6	up	carassius auratus tnfsf11 mrna for tumor necrosis factor ligand superfamily member partial cds
Ssa#STIR09479	TC66072	10.5	up	hepcidin
Ssa#STIR10915	TC67934	10.1	up	---NA---
Ssa#STIR15435	TC74269	10.1	up	: Taeniopygia guttata similar to receptor-interacting serine-threonine kinase 2
Ssa#CL13Contig1	CL13Contig1	10.0	up	Collagenase 3
Omy#S24139122	S24139122	10.0	up	bone morphogenetic protein 7
Omy#CT566393	CT566393	9.9	up	transferrin
Ssa#STIR06556	gi|209154871	9.8	up	member ras oncogene family
Ssa#STIR25488	TC89311	9.6	up	kiaa1872 protein
Ssa#S35697434	S35697434	9.6	up	CCAAT/enhancer binding protein beta2
Ssa#S43134841_S	NM_001123611	9.5	up	CD4-like protein
Ssa#EG762540	EG762540	9.5	up	mus musculus pantothenate kinase 1 transcript variant mrna
Ssa#STIR09973	TC66684	9.4	up	c-type lectin domain family 4 member e
Ssa#KSS4341	KSS4341	9.2	up	similar to phosphodiesterase 4B, cAMP-specific isoform 2
Ssa#AM049745	AM049745	9.2	up	haptoglobin
Ssa#S18835342	S18835342	9.1	up	differentially regulated trout protein 1
Ssa#STIR03264	gi|209735475	9.1	up	lymphocyte g0 g1 switch protein 2
Omy#gi197096774	gi197096774	8.9	up	Oncorhynchus mykiss partial il-8 gene for interleukin 8, promoter region
Ssa#TC84694_S	TC84694	8.9	up	SJCHGC03026 protein
Ssa#STIR22054	TC84154	8.9	up	c-type lectin domain family 4 member e
Omy#S38753637	S38753637	8.8	up	CCAAT/enhancer binding protein gamma
Ssa#S35582566	S35582566	8.8	up	Caspase-14 precursor
Ssa#STIR04098	gi|209733803	8.8	up	chemokine (c-x-c motif) ligand 10
Ssa#S35477400	S35477400	8.7	up	tetraodon nigroviridis full-length cdna
Ssa#S30259776	DW546744	8.7	up	sphingomyelin synthase 1
Omy#gi20270914	gi20270914	8.6	up	Oncorhynchus mykiss clone B191 VHSV-induced C-lectin-like protein mRNA
Ssa#S35492233	S35492233	8.5	up	Tartrate-resistant acid phosphatase type 5 precursor
Ssa#STIR00084_4	DW569632	8.5	up	nf-kappa-b inhibitor alpha
Ssa#KSS4516	KSS4516	8.5	up	B-cell linker protein
Ssa#STIR00160_4	gi|209736931	8.4	up	hepcidin
Ssa#STIR11153	TC68266	8.4	up	chemokine (c-x-c motif) receptor 3
Omy#TC157410	TC157410	8.3	up	synthetic construct homo sapiens clone wingless-type mmtv integration site family member 5a
Ssa#STIR20350	TC81573	8.3	up	---NA---
Ssa#S30266930	DW553898	8.3	up	Angiotensinogen
Omy#BX082697	BX082697	8.3	up	NF-kappa-B inhibitor alpha
Omy#BX319519	BX319519	8.2	up	BCL2/adenovirus E1B 19kD interacting protein like
Ssa#STIR00132_4	gi|209155113	8.1	up	tumor necrosisalpha-induced protein 2
Ssa#S35606391	S35606391	8.1	up	Sulfotransferase 6B1
Ssa#S35680120_S	S35680120	8.0	up	unnamed protein product
Ssa#TC104355	TC104355	7.9	up	Rho-related GTP-binding protein RhoE precursor
Ssa#KSS2955	KSS2955	7.8	up	Tumor necrosis factor, alpha-induced protein 2
Ssa#STIR12491	TC70097	7.8	up	---NA---
Omy#CA385474	CA385474	7.7	up	rattus norvegicus fat tumor suppressor homolog 4 mrna
Ssa#S35498882	S35498882	7.7	up	Regulator of G-protein signaling 5
Ssa#STIR00012_2	AY848944	7.6	up	prostaglandin-endoperoxide synthase 2 (prostaglandin g h synthase and cyclooxygenase)
Ssa#S30281294	S30281294	7.5	up	Bifunctional 3-phosphoadenosine 5-phosphosulfate synthetase 2
Ssa#TC102289	TC102289	7.4	up	homo sapiens anthrax toxin receptor 2 transcript variant mrna
Ssa#STIR07882	TC64103	7.4	up	Salmo salar clone ssal-rgb2-578-320 Ferritin, lower subunit putative mRNA,
Ssa#AJ424540	AJ424540	7.4	up	Collagenase 3
Ssa#STIR00132_3	gi|209155113	7.3	up	tumor necrosisalpha-induced protein 2
Ssa#S30263781	S30263781	7.3	up	sphingomyelin synthase 1
Ssa#STIR09593	TC66216	7.3	up	glutathione peroxidase 3
Ssa#S18892082	S18892082	7.3	up	similar to vertebrate solute carrier family 2 (facilitated glucose transporter), member 6 (SLC2A6)
Ssa#S35533728	S35533728	7.3	up	Collagenase 3
Ssa#S35693720	S35693720	7.3	up	Leukocyte cell-derived chemotaxin 2 precursor
Ssa#STIR00084_3	DW569632	7.3	up	nf-kappa-b inhibitor alpha
Ssa#CL488Ctg1	CL488Contig1	7.3	up	hypothetical protein BRAFLDRAFT_94522
Ssa#STIR17441	TC77222	7.2	up	Salmo salar clone ssal-rgf-532-257 unknown large open reading frame mRNA, novel cds
Ssa#STIR24772	TC88226	7.2	up	Rho-related GTP-binding protein RhoE precursor putative mRNA,
Omy#S22907650	S22907650	7.2	up	TNFAIP3 interacting protein 1
Ssa#STIR22490	TC84817	7.2	up	Neurofilament light polypeptide
Omy#CA385480	CA385480	7.1	up	hypothetical protein LOC100149890
Ssa#S41489475	S41489475	7.1	up	tumor necrosis factor alpha
Ssa#S31966293_S	S31966293	7.0	up	similar to transmembrane protease, serine 5
Ssa#STIR18971	TC79489	6.9	up	---NA---
Ssa#S30291549	S30291549	6.8	up	novel protein similar to vertebrate interleukin-1 receptor-associated kinase 3 (IRAK3)
Ssa#S35590638	S35590638	6.8	up	Tumor necrosis factor, alpha-induced protein 2
Ssa#STIR02363	gi|209737289	6.8	up	hemagglutinin amebocyte aggregation factor precursor
Ssa#S35688317	S35688317	6.7	up	tissue factor
Omy#S29351043	S29351043	6.7	up	tumor necrosis factor, alpha-induced protein 2 homologue
Ssa#CL231Ctg1	CL231Contig1	6.7	up	hypothetical protein LOC568780
Omy#TC172616	TC172616	6.6	up	oncorhynchus mykiss gene for mature partial cds
Ssa#S30240353	S30240353	6.6	up	Secretagogin
Ssa#DY714453	DY714453	6.6	up	Tripartite motif-containing protein 47
Ssa#STIR00010_3	AY617117	6.6	up	interleukin-1 beta
Ssa#S30244746	S30244746	6.5	up	danio rerio wu:fb77e12 (wu:fb77e12) mrna
Ssa#STIR10876	TC67882	6.5	up	acyl-synthetase short-chain family member 1
Ssa#STIR02553	gi|209736899	6.4	up	hemagglutinin amebocyte aggregation factor precursor
Ssa#S30280915	S30280915	6.4	up	Regulator of G-protein signaling 5
Ssa#TC100333	TC100333	6.4	up	Lipopolysaccharide-induced tumor necrosis factor-alpha factor homolog
Ssa#STIR22551	TC84899	6.3	up	lipoprotein lipase
Ssa#STIR22901	TC85422	6.3	up	beta a1
Ssa#S30245972	S30245972	6.1	up	small protein effector 1 of Cdc42
Ssa#STIR39740	TC110595	6.1	up	Hemagglutinin/amebocyte aggregation factor precursor

Ssa#STIR07746	TC63946	6.1	up	---NA---
Omy#S15289032	S15289032	6.1	up	Lipopolysaccharide-induced tumor necrosis factor-alpha factor homolog
Ssa#TC108295	TC108295	6.0	up	Metalloreductase STEAP4
Ssa#TC102380	TC102380	6.0	up	TNFAIP3 interacting protein 1
Ssa#STIR04042	gi|209733915	6.0	up	lymphocyte g0 g1 switch protein 2
Ssa#S35559165_S	S35559165	6.0	up	VHSV-induced protein-10
Omy#S34424587	S34424587	6.0	up	hypothetical protein LOC564685
Ssa#DW471186_S	DW471186	5.9	up	Lipopolysaccharide-induced tumor necrosis factor-alpha factor homolog
Ssa#S35493922	S35493922	5.9	up	NF-kappa-B inhibitor alpha
Ssa#CA041540	CA041540	5.9	up	homo sapiens odd-skipped related 2 transcript variant mrna
Ssa#S30282687	S30282687	5.9	up	similar to TNFAIP3 interacting protein 1
Omy#BX081658	BX081658	5.8	up	hypothetical protein LOC734939
Ssa#S43134843	S43134843	5.8	up	T-cell surface glycoprotein CD4
Ssa#STIR02512	gi|209736987	5.8	up	lymphocyte g0 g1 switch protein 2
Omy#CX262098	CX262098	5.8	up	hypothetical protein LOC100126135
Ssa#STIR15794	TC74789	5.8	up	similar to vertebrate ataxin 2 (ATXN2) and a CpG island, complete sequence
Ssa#S35529741	S35529741	5.8	up	Interleukin-8
Ssa#STIR00132_2	gi|209155113	5.8	up	tumor necrosisalpha-induced protein 2
Omy#S34425162	S34425162	5.8	up	danio rerio misc_rna miscrna
Ssa#CK879362	CK879362	5.8	up	TPA: NADPH oxidase organizer 1
Ssa#STIR26783	TC91232	5.8	up	novel protein with a PH domain
Ssa#STIR07298	gi|209148084	5.8	up	guanine nucleotide binding protein (g protein)alpha inhibiting activity polypeptide 2
Ssa#S48402747	S48402747	5.7	up	Ectonucleotide pyrophosphatase/phosphodiesterase family member 2 precursor
Ssa#AM402522	AM402522	5.7	up	oncorhynchus keta clone haptoglobin partial sequence
Ssa#DY716619	DY716619	5.7	up	small protein effector 1 of Cdc42
Con_INTmed_11	gi|209155113|gb|BT045	5.6	up	TRAITS_Intensity_Control_tes_tsa_01D07_gal_sal_std_5p_11C_LOW1K
Ssa#S18849640	S18849640	5.6	up	ADP-ribosylation factor
Ssa#DY729561	DY729561	5.6	up	LOC791944 protein
Ssa#STIR13082	TC70911	5.6	up	ssal-rgf-537-067 6-phosphofructo-2-kinase/fructose-2,6-biphosphatase 3 putative mRNA
Ssa#STIR12109	TC69567	5.6	up	family with sequence similaritymember a
Ssa#DW557369_S	DW557369	5.6	up	F-box protein 46
Ssa#S25806768	S25806768	5.6	up	B-cell receptor-associated protein 29
Ssa#CL285Ctg1	CL285Contig1	5.5	up	CCAAT/enhancer-binding protein beta
Ssa#STIR42087_S	AM402598	5.5	up	complement protein component C7-1
Ssa#S31966292	S31966292	5.5	up	similar to transmembrane protease, serine 5
Ssa#TC105153	TC105153	5.5	up	similar to cGMP kinase type I alpha
Ssa#DY716725	DY716725	5.5	up	transcription factor EC
Ssa#S18848339	S18848339	5.5	up	tetraodon nigroviridis full-length cdna
Ssa#STIR20999	TC82540	5.5	up	similar to vertebrate ataxin 2 (ATXN2) and a CpG island, complete sequence
Ssa#TC105353	TC105353	5.5	up	Mecr protein
Ssa#STIR00010_2	AY617117	5.5	up	interleukin-1 beta
Ssa#S35566535	S35566535	5.4	up	CD4
Ssa#S35590193	S35590193	5.4	up	Interferon-induced protein 44
Ssa#S32011674	S32011674	5.4	up	Alcohol dehydrogenase
Ssa#BG935071	BG935071	5.4	up	tetraodon nigroviridis full-length cdna
Ssa#STIR23508	TC86350	5.4	up	---NA---
Ssa#STIR11277	TC68442	5.3	up	---NA---
Ssa#S35703038	S35703038	5.3	up	Caspase recruitment domain-containing protein 9
Ssa#STIR05753	gi|209730471	5.3	up	lymphocyte g0 g1 switch protein 2
Ssa#TC91379	TC91379	5.2	up	homo sapiens immunoresponsive 1 homolog mrna
Ssa#STIR21065	TC82652	5.2	up	---NA---
Omy#S32042888	S32042888	5.2	up	haptoglobin fragment 1
Ssa#STIR18540	TC78850	5.2	up	guanine nucleotide binding protein (g protein)alpha inhibiting activity polypeptide 2
Ssa#STIR16157	TC75298	5.2	up	tnfaip3-interacting protein 1
Ssa#STIR16984	TC76540	5.2	up	---NA---
Ssa#TC68131	TC68131	5.2	up	salmo salar fam43a mrna
Ssa#KSS4389	KSS4389	5.2	up	homo sapiens immunoresponsive 1 homolog mrna
Omy#CX034335	CX034335	5.1	up	Cbp/p300-interacting transactivator 2
Ssa#S35676559	S35676559	5.1	up	salmo salar clone ssal-rgf-529-261 b-cell receptor cd22 precursor pseudogene cds
Ssa#STIR00084_2	DW569632	5.1	up	nf-kappa-b inhibitor alpha
Omy#DV200037	DV200037	5.1	up	Tripartite motif-containing protein 25 (Zinc finger protein 147) (Efp) (RING finger protein 147)
Ssa#DY725800	DY725800	5.1	up	bone morphogenetic protein 5
Ssa#CL106Ctg1	CL106Contig1	5.1	up	NF-kappa-B inhibitor alpha
Ssa#S35542545_S	S35542545	5.1	up	TNFAIP3 interacting protein 1
Ssa#STIR03266	gi|209735471	5.1	up	barrier-to-autointegration factor
Ssa#TC81241	TC81241	5.0	up	Acetyl-coenzyme A synthetase 2-like, mitochondrial precursor
Ssa#STIR20862	TC82316	5.0	up	Salmo salar clone ssal-rgf-507-021, novel cds
Ssa#S31963829	S31963829	5.0	up	Retinol dehydrogenase 3
Ssa#S35660727	S35660727	5.0	up	complement protein component C7-1
Ssa#CK880429	CK880429	5.0	up	Radical S-adenosyl methionine domain-containing protein 2
Ssa#S35565814	S35565814	5.0	up	Metallophosphoesterase domain-containing protein 2
Omy#S23932999	S23932999	5.0	up	Oocyte zinc finger protein XlCOF20
Ssa#S35571159	S35571159	5.0	up	basic helix-loop-helix family, member e22
Ssa#STIR18872	TC79334	4.9	up	---NA---
Ssa#S31996892	S31996892	4.9	up	transmembrane protein 164
Ssa#STIR14164	TC72465	4.9	up	Salmo salar clone ssal-evf-556-228 Zinc finger matrin-type protein 5 putative mRNA,
Ssa#S35590792	S35590792	4.9	up	similar to Probable G-protein coupled receptor 114 precursor (G-protein coupled receptor PGR27)
Ssa#CX357047	CX357047	4.9	up	similar to vertebrate solute carrier family 2 (facilitated glucose transporter), member 6 (SLC2A6)
Ssa#S30290701	S30290701	4.9	up	NADPH--cytochrome P450 reductase
Ssa#CL368Ctg1	CL368Contig1	4.9	up	CD209 antigen-like protein E
Ssa#BQ035726	BQ035726	4.8	up	similar to mCG1046517
Ssa#TC103458	TC103458	4.8	up	G protein alpha i1
Ssa#S30287211	S30287211	4.8	up	danio rerio sc:d808 (sc:d808) mrna
Ssa#STIR20012	TC81071	4.8	up	novel protein
Ssa#DW556678	DW556678	4.8	up	glycoprotein A repetitions predominant precursor
Omy#gi185133787	gi185133787	4.8	up	Oncorhynchus mykiss tissue factor (tf), mRNA
Ssa#S35590638_S	S35590638	4.8	up	Tumor necrosis factor, alpha-induced protein 2
Ssa#TC103109	TC103109	4.8	up	similar to protein tyrosine kinase TecIV
Omy#BX889608	BX889608	4.8	up	hect domain and RLD 3
Ssa#STIR08687	TC65064	4.8	up	immune-lectin-like receptor 3
Ssa#S35562464	S35562464	4.8	up	similar to protein phosphatase 4, regulatory subunit 1-like
Ssa#STIR10019	TC66744	4.8	up	---NA---
Ssa#S35536987	S35536987	4.7	up	Platelet basic protein precursor
Ssa#STIR20028	TC81092	4.7	up	Arntl1a protein
Omy#TC136428	TC136428	4.7	up	gamma-aminobutyric acid receptor beta subunit
Ssa#STIR20578	TC81885	4.7	up	Salmo salar clone 257A09 TCR gamma locus region

Ssa#S30263433	S30263433	4.7	up	hypothetical protein LOC558108
Ssa#STIR22198	TC84364	4.7	up	---NA---
Ssa#STIR24327	TC87556	4.7	up	Salmo salar clone ssal-rgf-531-214 Interferon-induced protein 44 putative mRNA,
Ssa#S30245768	S30245768	4.7	up	Bdkrb2 protein
Ssa#STIR03335	gi|209735331	4.7	up	c-x-c motif chemokine 10 precursor
Ssa#EL698275	EL698275	4.7	up	Cysteine and glycine-rich protein 3
Ssa#DW576031	DW576031	4.6	up	transcription factor EC
Ssa#STIR17382	TC77140	4.6	up	---NA---
Ssa#STIR16728	TC76152	4.6	up	---NA---
Ssa#STIR00012_3	AY848944	4.6	up	prostaglandin-endoperoxide synthase 2 (prostaglandin g h synthase and cyclooxygenase)
Ssa#S30276901	S30276901	4.6	up	danio rerio gtp binding protein 2 mrna
Ssa#S22669043	AY462105	4.6	up	growth hormone receptor isoform 1 precursor
Ssa#STIR17013	TC76581	4.6	up	---NA---
Ssa#EG815196	EG815196	4.6	up	canis familiaris ran-binding protein 10 mrna
Ssa#S35542545	S35542545	4.6	up	TNFAIP3 interacting protein 1
Ssa#STIR08579	TC64937	4.6	up	tumor necrosisalpha-induced protein 2
Ssa#STIR21072	TC82660	4.6	up	---NA---
Omy#S18093277	S18093277	4.6	up	Lipopolysaccharide-induced tumor necrosis factor-alpha factor homolog
Ssa#S35538602	S35538602	4.5	up	tissue factor
Omy#S34421721	S34421721	4.5	up	follistatin-like 3 glycoprotein
Ssa#STIR19902	TC80917	4.5	up	Danio rerio zgc:103418, mRNA (cDNA clone MGC:103418 IMAGE:7233104),
Omy#S23939908	S23939908	4.5	up	forkhead box C1a
Ssa#S43134842	S43134842	4.5	up	CD4-2B-like protein
Ssa#STIR17467	TC77258	4.5	up	fibronectin 1
Ssa#S30239545	S30239545	4.4	up	---NA---
Ssa#S22839336	S22839336	4.4	up	cyclooxygenase 2
Ssa#KSS5249	KSS5249	4.4	up	Interferon-induced protein 44
Ssa#S35701601	S35701601	4.4	up	similar to pleckstrin homology-like domain, family B, member 1
Ssa#STIR04500	gi|209732999	4.4	up	lymphocyte g0 g1 switch protein 2
Ssa#STIR17883	TC77861	4.4	up	loc495244 protein
Ssa#S30243674	S30243674	4.4	up	Alanine aminotransferase 2-like
Omy#NP544348	NP544348	4.4	up	oncorhynchus mykiss gene for mature partial cds
Ssa#S32007685	S32007685	4.4	up	CEF-10
Omy#S34424380	S34424380	4.4	up	immune-related, lectin-like receptor 4
Ssa#STIR03632	gi|209734735	4.4	up	lymphocyte g0 g1 switch protein 2
Ssa#S18890026	S18890026	4.4	up	Probable protein COQ10, mitochondrial precursor
Ssa#STIR07904	TC64128	4.4	up	ccaat enhancer binding protein beta
Omy#gi20270930	gi20270930	4.4	up	Oncorhynchus mykiss clone B160 VHSV-induced mRNA, partial sequence
Ssa#STIR18943	TC79444	4.4	up	---NA---
Omy#CA377056	CA377056	4.4	up	villin 2
Ssa#STIR34406	TC102792	4.3	up	Nucb2b protein
Ssa#S35693229	S35693229	4.3	up	similar to Amine sulfotransferase (Sulfotransferase 3A1) (SULT-X2)
Ssa#S18835747	S18835747	4.3	up	tetraodon nigroviridis full-length cdna
Ssa#STIR02663	gi|209736679	4.3	up	chemokine (c-x-c motif) ligand 10
Ssa#STIR00073_4	CK882031	4.3	up	interferon-induced protein with tetratricopeptide repeats 1
Ssa#S35561655	S35561655	4.3	up	similar to UDP glucuronosyltransferase 2 family, polypeptide B17
Ssa#S35685163	S35685163	4.3	up	Cytidine deaminase
Ssa#STIR15951	TC75001	4.3	up	---NA---
Omy#S18145630	S18145630	4.3	up	NEDD8 ultimate buster 1
Ssa#TC112114	TC112114	4.3	up	danio rerio potassium channel tetramerisation domain containing ( ) mrna
Ssa#S35679930	S35679930	4.3	up	sodium-hydrogen exchange protein-beta
Ssa#STIR10919	TC67939	4.3	up	sjchgc06985 protein
Ssa#STIR07762	TC63964	4.3	up	Salmo salar clone ssal-rgf-537-067 6-phosphofructo-2-kinase/fructose-2,6-biphosphatase 3
Ssa#TC84485	TC84485	4.3	up	: UDP-GlcNAc:betaGal beta-1,3-N-acetylglucosaminyltransferase 5
Ssa#S35665025	S35665025	4.3	up	Interferon-induced protein 44
Ssa#S43134841	S43134841	4.3	up	CD4-like protein
Ssa#S31963661	S31963661	4.3	up	salmo salar transcription factor etv6 mrna
Ssa#STIR08324	TC64640	4.2	up	---NA---
Ssa#STIR04406	gi|209733187	4.2	up	c-x-c motif chemokine 10 precursor
Ssa#DW582404	DW582404	4.2	up	similar to cadherin 2, type 1 preproprotein
Ssa#STIR02395	gi|209737225	4.2	up	lymphocyte g0 g1 switch protein 2
Ssa#STIR24132	TC87294	4.2	up	Oncorhynchus mykiss mitogen-activated protein kinase kinase kinase kinase 4-like protein gene
Ssa#STIR12498	TC70105	4.2	up	chemokine (c-x-c motif) ligand 10
Ssa#STIR18587	TC78923	4.2	up	t-cell surface glycoprotein cd4 precursor
Ssa#STIR26041	TC90144	4.2	up	---NA---
Omy#TC155623	TC155623	4.2	up	danio rerio misc_rna miscrna
Ssa#CX358043	CX358043	4.2	up	Lipase member H precursor
Ssa#STIR23181	TC85848	4.1	up	retinol dehydrogenase 3
Ssa#S48442658	S48442658	4.1	up	M17 homologue
Ssa#STIR15330	TC74132	4.1	up	---NA---
Ssa#DW544989	DW544989	4.1	up	putative transposase
Ssa#S31970169	S31970169	4.1	up	Transmembrane protein 100
Ssa#TC87660	TC87660	4.1	up	kinase C delta type
Ssa#S35678167	S35678167	4.1	up	endonuclease/exonuclease/phosphatase family domain containing 1
Ssa#STIR18449	TC78713	4.1	up	glutathione peroxidase 3
Ssa#STIR14034	TC72286	4.1	up	Salmo salar clone ssal-rgf-519-201, novel cds
Ssa#CL382Ctg1	CL382Contig1	4.1	up	NF-kappa-B inhibitor alpha
Omy#S34424313	S34424313	4.1	up	danio rerio ring finger protein mrna (cdna clone mgc:191581 image:100059890)
Ssa#DW553253	DW553253	4.1	up	Nucleobindin 2a
Ssa#STIR09120	TC65618	4.1	up	Zebrafish DNA sequence from clone CH73-70I4 in linkage group 16, complete sequence
Ssa#STIR20045	TC81116	4.1	up	neural cell adhesion molecule 2
Ssa#EG809754	EG809754	4.0	up	serpin peptidase inhibitor, clade E (nexin, plasminogen activator inhibitor type 1), member 1
Ssa#KSS4731	KSS4731	4.0	up	salmo salar clone ssal-rgf-529-261 b-cell receptor cd22 precursor pseudogene cds
Ssa#TC111777	TC111777	4.0	up	: wu:fj85b02
Ssa#S18836083	S18836083	4.0	up	tetraodon nigroviridis full-length cdna
Ssa#STIR07931	TC64162	4.0	up	Salmo salar clone ssal-rgf-507-021, novel cds
Omy#DV192811	DV192811	4.0	up	homo sapiens immunoresponsive 1 homolog mrna
Ssa#KSS4495	KSS4495	4.0	up	interferon regulatory factor 7
Omy#S15341085	S15341085	4.0	up	chemokine CK-1 precursor
Ssa#EG826133	EG826133	4.0	up	Cbp/p300-interacting transactivator 2
Ssa#S35678013	S35678013	4.0	up	: novel NACHT domain containing protein
Ssa#S35670682	S35670682	4.0	up	Wilms' tumor suppressor 2b
Ssa#CX357114	CX357114	3.9	up	Wnt9a
Ssa#S35694475	S35694475	3.9	up	TNFAIP3 interacting protein 1
Ssa#S31984657	S31984657	3.9	up	Tumor necrosis factor receptor superfamily member 5
Ssa#S30239956	S30239956	3.9	up	NF-kappa-B inhibitor alpha

Ssa#S35663525	S35663525	3.9	up	hypothetical protein LOC436879
Ssa#S30265696	S30265696	3.9	up	hypothetical protein
Ssa#STIR13314	TC71232	3.9	up	immune-lectin-like receptor 3
Ssa#S18892257	AJ505008	3.9	up	interleukin 1 receptor accessory protein
Ssa#STIR14647	TC73172	3.9	up	Contains the mknk2 gene for MAP kinase-interacting serine/threonine kinase 2
Ssa#S35580297	S35580297	3.9	up	Cysteine and glycine-rich protein 3
Ssa#STIR22311	TC84526	3.9	up	prostaglandin e receptorsubtype ep2
Omy#BX883524	BX883524	3.9	up	activin beta A precursor
Omy#TC154779	TC154779	3.9	up	danio rerio hypothetical loc555629 mrna
Ssa#STIR15699	TC74643	3.9	up	---NA---
Ssa#S30276405	DW563373	3.9	up	suppressor of cytokine signaling 1
Ssa#S32011160	S32011160	3.8	up	Androgen-induced proliferation inhibitor
Ssa#STIR12227	TC69734	3.8	up	---NA---
Ssa#DY724777	DY724777	3.8	up	Probable tubulin polyglutamylase TTLL1
Ssa#S32008132	S32008132	3.8	up	bone morphogenetic protein 6
Ssa#S35590181	S35590181	3.8	up	serpin peptidase inhibitor, clade E (nexin, plasminogen activator inhibitor type 1), member 1
Ssa#S18836991_S	S18836991	3.8	up	Hemoglobin subunit alpha-4
Ssa#CA048716	CA048716	3.8	up	Integrin beta-1 precursor
Ssa#STIR09745	TC66402	3.8	up	---NA---
Ssa#S35683074	S35683074	3.8	up	hypothetical protein
Ssa#CL344Ctg1	CL344Contig1	3.8	up	CCAAT/enhancer binding protein beta2
Ssa#STIR10893	TC67903	3.8	up	ubiquitin-like protein 1
Ssa#S35674656	S35674656	3.8	up	LIM domain kinase 1
Ssa#STIR12571	TC70217	3.8	up	TSA: Hippoglossus hippoglossus all_halibut.2451.C1 mRNA sequence
Ssa#STIR09145	TC65652	3.7	up	---NA---
Omy#CA370819	CA370819	3.7	up	---NA---
Ssa#TC94054	TC94054	3.7	up	Leukocyte cell-derived chemotaxin 2 precursor
Ssa#CK887906	CK887906	3.7	up	transposase
Ssa#STIR04257	gi|209733485	3.7	up	lymphocyte g0 g1 switch protein 2
Ssa#STIR20703	TC82084	3.7	up	glutamic pyruvate transaminase (alanine aminotransferase) 2
Ssa#DW537229	DW537229	3.7	up	transmembrane protein 200A
Ssa#DW565773	DW565773	3.7	up	: transmembrane protein 200A
Ssa#STIR18005	TC78042	3.7	up	interleukin 20alpha
Ssa#KSS3052	KSS3052	3.7	up	Interferon-induced protein with tetratricopeptide repeats 5
Ssa#STIR44286	DY694909	3.7	up	vascular cell adhesion molecule-like protein
Ssa#DW572300	DW572300	3.7	up	Alcohol dehydrogenase
Ssa#CK879399	CK879399	3.6	up	Hexokinase 1
Ssa#STIR00073_3	CK882031	3.6	up	interferon-induced protein with tetratricopeptide repeats 1
Ssa#EG828208	EG828208	3.6	up	Alcohol dehydrogenase
Ssa#STIR18501	TC78793	3.6	up	---NA---
Ssa#S32010071	S32010071	3.6	up	dopachrome tautomerase
Ssa#S30240058	S30240058	3.6	up	3-oxo-5-alpha-steroid 4-dehydrogenase 2
Ssa#EG904665	EG904665	3.6	up	similar to formin, inverted
Ssa#S35565503	S35565503	3.6	up	DNA-damage-inducible transcript 4 protein
Ssa#STIR09597	TC66221	3.6	up	sulfotransferase 6b1
Ssa#DW575324	DW575324	3.6	up	rattus norvegicus chromobox homolog 7 mrna
Ssa#STIR12701	TC70393	3.6	up	retinol dehydrogenase 3
Ssa#STIR19401	TC80134	3.6	up	---NA---
Ssa#KSS5048	KSS5048	3.6	up	DNA-damage-inducible transcript 4 protein
Ssa#KSS4154	KSS4154	3.6	up	TRAF2-binding protein
Ssa#S35490809	S35490809	3.6	up	Band 4.1-like protein 4
Ssa#KSSb2765	KSSb2765	3.6	up	Receptor-transporting protein 3
Omy#S22912358	S22912358	3.6	up	G protein-coupled receptor 157
Ssa#S35538989	S35538989	3.5	up	aryl hydrocarbon receptor nuclear translocator-like 1a
Ssa#STIR14402	TC72823	3.5	up	---NA---
Ssa#S35703486	S35703486	3.5	up	salmo salar clone ssal-rgf-529-261 b-cell receptor cd22 precursor pseudogene cds
Ssa#KSS2007	KSS2007	3.5	up	Myelin-associated glycoprotein
Ssa#STIR10396	TC67245	3.5	up	---NA---
Ssa#DW540525	DW540525	3.5	up	novel protein similar to gelsolin, like 1 (gsnl1, zgc:77481)
Ssa#S18855772	S18855772	3.5	up	Bloodthirsty
Ssa#S35559407	S35559407	3.5	up	Sulfotransferase 6B1
Ssa#STIR31819	TC98944	3.5	up	glucose-6-phosphate-1-dehydrogenase
Omy#S32802584	S32802584	3.5	up	macrophage myristoylated alanine-rich C kinase-like protein
Ssa#STIR12507	TC70118	3.5	up	syndecan 4
Ssa#S18833375	S18833375	3.5	up	similar to perilipin
Ssa#TC82465	TC82465	3.5	up	Kctd12.2 protein
Ssa#S32012246	S32012246	3.5	up	F-box/LRR-repeat protein 5
Ssa#KSS4555	KSS4555	3.5	up	Chloride intracellular channel protein 2
Ssa#STIR00011	AY708653	3.5	up	caspase-9
Ssa#STIR21746	TC83679	3.5	up	casp8 and fadd-like apoptosis regulator
Ssa#S35472445	S35472445	3.5	up	Fn1 protein
Ssa#CX356711	CX356711	3.5	up	Cbp/p300-interacting transactivator, with Glu/Asp-rich carboxy-terminal domain, 2
Ssa#S35520917	S35520917	3.5	up	drebrin-like
Ssa#CK886748	CK886748	3.5	up	cytochrome b-245, beta polypeptide
Omy#S34423978	S34423978	3.5	up	Sequestosome-1
Ssa#BG934050	BG934050	3.5	up	kinase C delta type
Ssa#S31975661	S31975661	3.4	up	similar to centaurin, delta 2,
Ssa#STIR17046	TC76631	3.4	up	Salmo salar clone ssal-rgf-521-313 unknown large open reading frame mRNA, novel cds
Ssa#S31990924	S31990924	3.4	up	Probable protein COQ10, mitochondrial precursor
Ssa#STIR22086	TC84201	3.4	up	Pongo abelii BAC clone CH276-340B19 from chromosome unknown, complete sequence
Ssa#CX355009	CX355009	3.4	up	Interferon regulatory factor 3
Ssa#S35564673	S35564673	3.4	up	NF-kappa-B inhibitor alpha
Ssa#CK884742	CK884742	3.4	up	Cytosolic non-specific dipeptidase
Ssa#STIR15354	TC74165	3.4	up	mitogen-activated protein kinase kinase kinase 8
Omy#S34425511	S34425511	3.4	up	rattus norvegicus pseudouridylate synthase 7 homolog (cerevisiae) mrna
Ssa#KSS3231	KSS3231	3.4	up	Sequestosome-1
Ssa#CX357223	CX357223	3.4	up	Cytochrome P450 7B1
Ssa#S31978932	S31978932	3.4	up	Sequestosome-1
Ssa#S30295513	S30295513	3.4	up	putative homeodomain transcription factor 1
Ssa#TC104293	TC104293	3.4	up	Sequestosome-1
Ssa#STIR08426	TC64760	3.4	up	sjchgc03026 protein
Ssa#STIR13286	TC71194	3.4	up	Salmo salar clone ssal-rgf-519-251 Apolipoprotein-L3 putative mRNA,
Ssa#STIR24788	TC88255	3.4	up	high affinity immunoglobulin gamma fc receptor i precursor
Ssa#S35667643	EG902361	3.4	up	Complement C1q-like protein 4
Omy#CA377958	CA377958	3.4	up	LOC555629 protein
Ssa#S35475340	S35475340	3.4	up	Platelet basic protein

Ssa#S30242214	S30242214	3.4	up	Receptor-transporting protein 3
Ssa#KSS628	KSS628	3.4	up	SH3 and PX domain-containing protein 2A
Ssa#S35660755	EG895473	3.4	up	Nuclear factor NF-kappa-B p100 subunit
Ssa#STIR21272	TC82967	3.4	up	interferon inducible mx protein
Ssa#STIR23437	TC86236	3.4	up	alpha actin
Ssa#S35697292	S35697292	3.4	up	Chloride intracellular channel protein 2
Ssa#STIR00073_2	CK882031	3.3	up	interferon-induced protein with tetratricopeptide repeats 1
Ssa#STIR21694	TC83608	3.3	up	Salmo salar clone ssal-rgf-527-326 NAD kinase putative mRNA
Ssa#S35550575	S35550575	3.3	up	similar to Patched domain-containing protein 3 (RND-type protein RNDEu-3)
Ssa#S31979481	S31979481	3.3	up	salmo salar clone ssal-rgf-518-353 heterogeneous nuclear ribonucleoprotein r
Ssa#TC91713	TC91713	3.3	up	SH3 and PX domain-containing protein 2A
Ssa#STIR21739	TC83671	3.3	up	myelin-associated glycoprotein
Ssa#STIR18514	TC78810	3.3	up	Salmo salar clone ssal-rgf-519-251 Apolipoprotein-L3 putative mRNA,
Ssa#S35582370	S35582370	3.3	up	DnaJ homolog subfamily C member 3
Ssa#STIR11364	TC68558	3.3	up	chemokine cxcl-c1c
Ssa#S35501248	S35501248	3.3	up	Integrin beta-3 precursor
Ssa#S30054066	S30054066	3.3	up	: novel protein similar to mucosa associated lymphoid tissue lymphoma translocation gene 1 (malt1)
Ssa#S31979589	S31979589	3.3	up	Cysteine and glycine-rich protein 1
Ssa#S48394398	S48394398	3.3	up	tetraspanin 13
Ssa#CL424Ctg1	CL424Contig1	3.3	up	similar to Protein asteroid homolog 1
Ssa#CL177Ctg1	CL177Contig1	3.3	up	cytochrome b-245, beta polypeptide
Ssa#S31987402_S	S31987402	3.3	up	NADH dehydrogenase 1 alpha subcomplex subunit 4-like 2
Ssa#STIR00073	CK882031	3.3	up	Interferon-induced protein with tetratricopeptide repeats 5 (IFIT-5)
Ssa#STIR14914	TC73545	3.3	up	Oncorhynchus mykiss macrophage myristoylated alanine-rich C kinase-like protein mRNA,
Ssa#STIR00067_3	U66477	3.3	up	interferon inducible mx protein
Ssa#S31983293	S31983293	3.3	up	Rho-related GTP-binding protein RhoE
Ssa#S48410674	S48410674	3.3	up	SYM1
Ssa#TC77098	TC77098	3.3	up	similar to human immunodeficiency virus type I enhancer binding protein 1
Ssa#S35588230	S35588230	3.3	up	ADP-ribosylation factor 4
Ssa#TC112660_S	TC112660	3.3	up	ADP-ribose/CDP-alcohol pyrophosphatase
Ssa#TC101720	TC101720	3.3	up	similar to Shaw-related voltage-gated potassium channel protein 4 isoform a isoform 1
Ssa#STIR02057	gi|209737903	3.3	up	protein tyrosine phosphatase-like (proline instead of catalytic arginine)member b
Ssa#S35528810	EG815188	3.2	up	Ubiquitin-like protein 1
Ssa#CK897705	CK897705	3.2	up	phosphatase 1 regulatory subunit 1C
Omy#S22922238	S22922238	3.2	up	similar to conserved hypothetical protein
Ssa#TC107862_S	TC107862	3.2	up	cytochrome P450
Ssa#STIR11368	TC68563	3.2	up	Salmo salar clone ssal-rgf-535-211 Probable palmitoyltransferase ZDHHC13 putative mRNA,
Ssa#STIR11230	TC68378	3.2	up	Salmo salar clone ssal-rgf-524-099 S-adenosylmethionine synthetase isoform type-2
Ssa#S48420588	NM_001173773	3.2	up	Myotubularin
Ssa#STIR22578	TC84941	3.2	up	INSL5 gene for insulin-like 5 and the 3' end of a novel gene (FLJ23129), complete sequence
Omy#S15245231	S15245231	3.2	up	hypothetical protein LOC100003142
Ssa#STIR08772	TC65168	3.2	up	chemokine (c-c motif) ligand 8
Ssa#S35534306	S35534306	3.2	up	salmo salar fxyd domain containing ion transport regulator 5b mrna
Ssa#STIR05157	gi|209731669	3.2	up	lymphocyte g0 g1 switch protein 2
Ssa#STIR04277	gi|209733445	3.2	up	limb bud and heart development homolog
Ssa#STIR00011_2	AY708653	3.2	up	caspase 9
Ssa#S35515578	S35515578	3.2	up	similar to cardiac ankyrin repeat protein
Ssa#S30281534	S30281534	3.2	up	Interferon-induced protein with tetratricopeptide repeats 5
Ssa#S31992293	DY720890	3.2	up	Interleukin-10 receptor beta chain precursor
Ssa#S35559407_S	S35559407	3.2	up	Sulfotransferase 6B1
Ssa#STIR11118	TC68218	3.2	up	kinesin family memberpartial
Ssa#STIR23855	TC86869	3.2	up	hypoxia up-regulated 1
Ssa#STIR23865	TC86882	3.2	up	: novel protein similar to mucosa associated lymphoid tissue lymphoma translocation gene 1 (malt1)
Omy#S32127737	S32127737	3.2	up	novel protein (zgc:163136)
Ssa#STIR24004	TC87105	3.1	up	Zebrafish DNA sequence from clone DKEY-193P22, complete sequence
Ssa#S35591126	S35591126	3.1	up	Radical S-adenosyl methionine domain-containing protein 2
Ssa#STIR14969	TC73619	3.1	up	udp-n-acetyl-alpha-d-galactosamine:polypeptide n-acetylgalactosaminyltransferase 6 (c-t6)
Ssa#CB514915	CB514915	3.1	up	Alcohol dehydrogenase
Ssa#STIR03719	gi|209734561	3.1	up	chemokine (c-c motif) ligand 8
Ssa#S18888694	S18888694	3.1	up	immune-related, lectin-like receptor 4
Omy#KVkm2_F07	KVkm2	3.1	up	HEPC1_SALSARecName: Full=Hepcidin-1; Flags: Precursor
Ssa#STIR15975	TC75035	3.1	up	patched domain-containing protein 3 (rnd-type protein rndeu-3)
Ssa#DY695499	DY695499	3.1	up	AF156738_1decoy TNF receptor
Ssa#S35581889	S35581889	3.1	up	hypothetical protein LOC541512
Ssa#S31965464	S31965464	3.1	up	Complement C1q-like protein 4 precursor
Omy#BX082318	BX082318	3.1	up	RAB26, member RAS oncogene family
Ssa#S32000624	S32000624	3.1	up	Polypeptide N-acetylgalactosaminyltransferase 6
Ssa#S30289644	S30289644	3.1	up	Mediator of RNA polymerase II transcription subunit 16
Ssa#S35567805	S35567805	3.1	up	Rieske domain-containing protein
Ssa#KSSb2302	KSSb2302	3.1	up	Metalloproteinase inhibitor 2
Ssa#DV106832_S	DV106832	3.1	up	Tax1 binding protein 1
Ssa#S30295849	S30295849	3.1	up	similar to mCG1046517
Ssa#S31971912	S31971912	3.1	up	interferon-inducible protein IFI58
Ssa#DW565154	DW565154	3.1	up	UDP-xylose and UDP-N-acetylglucosamine transporter
Ssa#KSS2124	KSS2124	3.1	up	Interleukin-10 receptor beta chain precursor
Ssa#DY696621	DY696621	3.0	up	Kruppel-like factor 11
Ssa#S35658715	S35658715	3.0	up	ATP-binding cassette, sub-family C (CFTR/MRP), member 4
Ssa#STIR14251	TC72585	3.0	up	Zebrafish DNA sequence from clone CH211-205B11 in linkage group 3, complete sequence
Ssa#CL17Contig1	CL17Contig1	3.0	up	Matrix metalloproteinase-9
Omy#S23923283	S23923283	3.0	up	tumor necrosis factor, alpha-induced protein 2
Ssa#S31996253	S31996253	3.0	up	Myelin-associated glycoprotein
Ssa#TC77476	TC77476	3.0	up	Caspase recruitment domain-containing protein 9
Ssa#S35583267	S35583267	3.0	up	Galectin-9
Omy#CA377250	CA377250	3.0	up	danio rerio skin mucus antibacterial l-amino acid oxidase mrna
Ssa#S32004881	S32004881	3.0	up	apoptotic protease activating factor
Ssa#STIR05336	gi|209731305	3.0	up	rho gtpase activating protein 21
Omy#gi197927463	NM_001124396	3.0	up	Oncorhynchus mykiss interleukin-1 receptor antagonist (il-1ra), mRNA
Omy#BX309611	BX309611	3.0	up	: hm:gc12
Ssa#S35675802	S35675802	3.0	up	Apoptosis-associated speck-like protein containing a CARD
Ssa#S31979355	S31979355	3.0	up	Programmed cell death 1 ligand 1
Ssa#STIR22057	TC84158	3.0	up	Cell death activator CIDE-3
Ssa#S35685185	S35685185	3.0	up	similar to AGAP012527-PA
Ssa#STIR19967	TC81010	3.0	up	Salmo salar clone ssal-rgf-529-067 TRAF2-binding protein putative mRNA,
Ssa#STIR00087_3	DW555246	3.0	up	interleukin 10beta
Ssa#S35579780	S35579780	3.0	up	THUMP domain-containing protein 3
Ssa#STIR02910	gi|209736185	3.0	up	eukaryotic translation initiation factor 2-alpha kinase 2

Ssa#STIR20848	TC82297	3.0	up	g protein-coupled receptor 109a
Ssa#STIR25467	TC89277	3.0	up	Salmo salar BAC CH214-363E24) complete sequence
Ssa#STIR15853	TC74876	3.0	up	radixin
Ssa#TC111296	TC111296	3.0	up	similar to WD repeat-containing protein 26
Ssa#S35537283	S35537283	3.0	up	C2orf55
Ssa#STIR18109	TC78204	3.0	up	Zebrafish DNA sequence from clone CH73-22B20 in linkage group 8
Ssa#KSS1929	KSS1929	3.0	up	Immediate early response gene 2 protein
Ssa#TC63531	TC63531	3.0	up	transposase
Ssa#STIR11151	TC68263	3.0	up	cytochrome b-alpha polypeptide
Ssa#STIR17457	TC77243	3.0	up	unc-84 homolog a
Ssa#STIR04236	gi|209733527	3.0	up	kda protein
Omy#gi185132470	gi185132470	2.9	up	Oncorhynchus mykiss VHSV-induced protein-4 (LOC100135996), mRNA
Ssa#KSS3064	KSS3064	2.9	up	salmo salar eap30 subunit of ell complex a nuclear domain 10 protein 52 a tubulin tyrosine ligase-like
Ssa#S31962884	DY691674	2.9	up	Cytochrome c oxidase subunit 5B, mitochondrial precursor
Omy#S32040967	S32040967	2.9	up	DnaJ homolog subfamily C member 3
Ssa#STIR11905	TC69285	2.9	up	Gallus gallus BAC clone TAM31-15L4 from chromosome z, complete sequence
Ssa#CX357917	CX357917	2.9	up	DnaJ homolog subfamily C member 3
Ssa#S35483223	S35483223	2.9	up	gallus gallus carp mrna for cardiac ankyrin repeat
Ssa#STIR11974	TC69389	2.9	up	Salmo salar physical map contig 483, genomic sequence
Ssa#STIR08015	TC64260	2.9	up	high affinity immunoglobulin gamma fc receptor i precursor
Ssa#STIR13778	TC71920	2.9	up	OMY315933Oncorhynchus mykiss mRNA for type II keratin E3 (E3 gene)
Ssa#STIR22427	TC84716	2.9	up	stress 70 proteinmicrosome-60kda
Ssa#S19101960	S19101960	2.9	up	Metalloproteinase inhibitor 2 precursor
Ssa#CB512304_S	CB512304	2.9	up	similar to proline dehydrogenase (oxidase) 1
Ssa#STIR12617	TC70276	2.9	up	---NA---
Ssa#STIR04994	gi|209731999	2.9	up	adenylate kinase 3 alpha like 1 variant 1
Ssa#gi209737173	gi209737173	2.9	up	Salmo salar clone ssal-eve-562-294 Radical S-adenosyl methionine domain-containing protein 2
Ssa#S30290800	S30290800	2.9	up	5-aminolevulinate synthase, nonspecific, mitochondrial precursor
Ssa#STIR25186	TC88856	2.9	up	guanine nucleotide bindingalpha inhibiting 1
Ssa#KSS392	NM_001141766	2.9	up	Interleukin-1 receptor-associated kinase 4
Ssa#STIR08885	TC65310	2.9	up	Sequence from clone CH211-195O19 in linkage group 20 Contains the 5' end of a novel gene
Ssa#STIR25114	TC88752	2.9	up	suppressor of cytokine signaling 1
Ssa#S30241339	S30241339	2.9	up	similar to G protein-coupled receptor kinase 5 (G protein-coupled receptor kinase GRK5)
Ssa#S35518234	EG804612	2.9	up	CCAAT/enhancer binding protein delta
Ssa#S30296175	S30296175	2.9	up	rattus norvegicus potential ubiquitin ligase mrna
Ssa#TC93895	TC93895	2.8	up	danio rerio plexin d1
Ssa#S18892315	S18892315	2.8	up	Mx3 protein
Ssa#S35554999	S35554999	2.8	up	similar to LAG1 homolog, ceramide synthase 6
Ssa#S18890005	CB515535	2.8	up	Probable E3 ubiquitin-protein ligase RNF144A-A
Ssa#TC69209	TC69209	2.8	up	homo sapiens chromosome clone rp11- complete sequence
Ssa#STIR14140	TC72437	2.8	up	---NA---
Ssa#KSS2182	KSS2182	2.8	up	Ubiquitin
Ssa#KSS683	KSS683	2.8	up	Receptor-transporting protein 3
Ssa#STIR07722	TC63917	2.8	up	metalloproteinase inhibitor 2 precursor
Ssa#EG819142	EG819142	2.8	up	bos taurus glutaminyl-peptide cyclotransferase-like
Ssa#S30275870	S30275870	2.8	up	Immediate early response gene 2 protein
Ssa#S30266067	S30266067	2.8	up	gasterosteus aculeatus clone ch213- complete sequence
Ssa#S35552846	S35552846	2.8	up	Phosphatidylinositol N-acetylglucosaminyltransferase subunit H
Ssa#STIR22723	TC85163	2.8	up	gtp binding protein 2
Ssa#CB516003	CB516003	2.8	up	NF-kappa-B 1 p105 subunit
Omy#S18155933	S18155933	2.8	up	hypothetical protein LOC100148091
Ssa#S31963975	S31963975	2.8	up	asteroid homolog 1
Ssa#STIR17566	TC77403	2.8	up	---NA---
Ssa#S35025390	S35025390	2.8	up	Hypoxia up-regulated protein 1 precursor
Omy#TC161674	TC161674	2.8	up	transcription factor mafK
Ssa#S35687929	S35687929	2.8	up	Receptor-transporting protein 3
Ssa#S30282764	S30282764	2.8	up	THUMP domain-containing protein 3
Ssa#STIR07614	TC63790	2.8	up	immunoglobulin superfamily member 6 precursor
Ssa#DY694205	DY694205	2.8	up	danio rerio zgc:153020 (zgc:153020) mrna
Ssa#TC92577	TC92577	2.8	up	interferon regulatory factor 8
Ssa#EG860899_S	EG860899	2.8	up	similar to SIL1 homolog, endoplasmic reticulum chaperone (S. cerevisiae)
Omy#S18162730	S18162730	2.8	up	si:dkey-33c12.4
Ssa#KSS3660	BT059477	2.8	up	NF-kappa-B inhibitor epsilon
Ssa#S35553608	S35553608	2.8	up	interleukin-1 receptor antagonist
Ssa#S35584868	S35584868	2.8	up	Tax1bp1b
Ssa#STIR22926	TC85458	2.8	up	protein phosphatase 2 (formerly 2a)regulatory subunit b (pr 52)beta isoform
Ssa#S31963491	DY692281	2.8	up	Peroxisomal proliferator-activated receptor A-interacting complex 285 kDa protein
Ssa#STIR04091	gi|209733817	2.8	up	lymphocyte g0 g1 switch protein 2
Ssa#KSSb2731	KSSb2731	2.8	up	Rieske domain-containing protein
Ssa#KSS1976	KSS1976	2.8	up	78 kDa glucose-regulated protein
Omy#S15248653	S15248653	2.8	up	AltName: Full=Activated leukocyte cell adhesion molecule
Ssa#STIR00067_2	U66477	2.8	up	interferon inducible mx protein
Ssa#TC84807	TC84807	2.8	up	Integrin beta 3b
Ssa#DW578491	DW578491	2.8	up	suppressor of cytokine signaling 3
Ssa#DY704666	DY704666	2.8	up	Nuclear factor NF-kappa-B p100 subunit
Omy#S15970698	S15970698	2.8	up	GalNAc alpha 2,6-sialyltransferase
Ssa#TC105439	TC105439	2.8	up	6-phosphofructokinase type C
Ssa#S35706273	S35706273	2.8	up	mus musculus oxysterol binding 9 transcript variant mrna
Ssa#STIR00154_4	SSU66475	2.8	up	interferon inducible mx protein
Ssa#STIR35646	TC104574	2.8	up	similar to sclerostin
Ssa#S35591008	S35591008	2.8	up	putative novel protein
Ssa#STIR05104	gi|209731777	2.8	up	lymphocyte g0 g1 switch protein 2
Ssa#STIR08031	TC64279	2.8	up	kda protein
Omy#TC163453	TC163453	2.8	up	Hyaluronan and proteoglycan link protein 3 precursor
Omy#S15298237	S15298237	2.8	up	AF466280_1beta-actin
Ssa#STIR11784	TC69122	2.8	up	family with sequence similaritymember c
Ssa#TC101511_S	TC101511	2.8	up	---NA---
Ssa#STIR08793	TC65192	2.8	up	Oncorhynchus mykiss mRNA for suppressor of cytokine signaling 3 (socs3 gene)
Ssa#S35506742	S35506742	2.8	up	Granulocyte colony-stimulating factor receptor precursor
Ssa#STIR25885	TC89916	2.8	up	---NA---
Ssa#CA048395	CA048395	2.8	up	interleukin-1 receptor antagonist
Ssa#S18866662_S	S18866662	2.8	up	Ugt1ab protein
Ssa#STIR11242	TC68391	2.8	up	---NA---
Ssa#STIR18691	TC79066	2.8	up	---NA---
Ssa#STIR21382	TC83144	2.8	up	chemokine (c-c motif) ligand 19
Ssa#S35541903	S35541903	2.8	up	SH3 and PX domain-containing protein 2A

Ssa#EG872587	EG872587	2.7	up	DnaJ (Hsp40) homolog, subfamily B, member 9
Ssa#STIR00110_2	BM413877	2.7	up	adipose differentiation-related protein
Ssa#S48429509_S	S48429509	2.7	up	Probable E3 ubiquitin-protein ligase RNF144A-A
Ssa#KSS3657	KSS3657	2.7	up	similar to mCG1046517
Ssa#DW564916	DW564916	2.7	up	Ubr5 protein
Ssa#S30241799	S30241799	2.7	up	similar to HERC6
Ssa#S35705796	S35705796	2.7	up	hypothetical protein LOC100148091
Ssa#S35587721	EG874099	2.7	up	Apolipoprotein-L3
Ssa#TC65653	TC65653	2.7	up	Tyrosine-protein kinase Lyn
Ssa#DW567010	DW567010	2.7	up	homo sapiens neural cell adhesion molecule 1 transcript variant mrna
Ssa#STIR00087_4	DW555246	2.7	up	interleukin 10beta
Ssa#DW182863	DW182863	2.7	up	DnaJ (Hsp40) homolog, subfamily B, member 12
Ssa#STIR07786	TC63995	2.7	up	interleukin 13alpha 1
Ssa#KSS1716	KSS1716	2.7	up	zebrafish dna sequence from clone dkeyp-118h9 in linkage group complete sequence
Ssa#S30271130	S30271130	2.7	up	similar to Leukotriene B4 receptor 1 (LTB4-R 1) (P2Y purinoceptor 7) (P2Y7)
Ssa#DY694724	DY694724	2.7	up	Tc1-like transporase
Ssa#EG865277	EG865277	2.7	up	activin B
Ssa#STIR22412	TC84686	2.7	up	ubiquitin protein ligase e3a
Ssa#S35585428	S35585428	2.7	up	Interleukin-10 receptor beta chain precursor
Omy#CA368603	CA368603	2.7	up	salmo salar mab-21-like 1 mrna
Ssa#TC87833	TC87833	2.7	up	LOC566027 protein
Ssa#STIR12660	TC70335	2.7	up	nedd8 ultimate buster 1
Ssa#S35606766	S35606766	2.7	up	Tumor necrosis factor receptor superfamily member 5
Ssa#KSS1693_S	KSS1693	2.7	up	LOC100148704 protein
Ssa#STIR07029	gi|209153915	2.7	up	chemokine receptor-like 1
Omy#TC146689	TC146689	2.7	up	NF-kappa-B inhibitor epsilon
Ssa#S35684862_S	S35684862	2.7	up	unnamed protein product
Omy#S15272923	S15272923	2.7	up	endoplasmic reticulum oxidoreductin 1-Lbeta
Ssa#S35501376	S35501376	2.7	up	DnaJ homolog subfamily C member 15
Ssa#STIR04308	gi|209733383	2.7	up	ring finger protein 138
Ssa#S30283255	S30283255	2.7	up	kynurenine 3-monooxygenase
Ssa#DW559682	DW559682	2.7	up	sel-1 suppressor of lin-12-like
Ssa#S35596870	S35596870	2.7	up	salmo salar clone ssal-rgf-538-016 cytosolic non-specific dipeptidase
Ssa#S32011218	S32011218	2.7	up	FIC domain containing
Ssa#S35576226	S35576226	2.7	up	similar to laminin alpha 3
Ssa#STIR16147	TC75285	2.7	up	---NA---
Ssa#STIR20008	TC81066	2.7	up	nedd4-binding protein 1
Ssa#EG860899	EG860899	2.7	up	similar to SIL1 homolog, endoplasmic reticulum chaperone (S. cerevisiae)
Ssa#S31970040	S31970040	2.7	up	similar to skin mucus antibacterial l-amino acid oxidase
Ssa#S35545523	S35545523	2.7	up	matrix metalloproteinase 13
Ssa#STIR21754	TC83692	2.7	up	---NA---
Ssa#KSS2803	KSS2803	2.7	up	Cysteine and glycine-rich protein 1
Ssa#STIR15703	TC74651	2.7	up	protein
Ssa#S18892319	S18892319	2.7	up	interleukin-1 receptor-like protein
Ssa#S18892317	S18892317	2.7	up	myxovirus resistance 1
Ssa#S25807320	S25807320	2.7	up	matrix metalloproteinase
Ssa#STIR08064	TC64317	2.7	up	Danio rerio insulin-like growth factor binding protein 5 (igfbp5), mRNA
Ssa#S30242693	S30242693	2.7	up	similar to LOC795887 protein
Ssa#TC77948	TC77948	2.7	up	RanBP-type and C3HC4-type zinc finger-containing protein 1
Ssa#STIR11134	TC68239	2.7	up	plexin d1
Omy#S23944498	S23944498	2.7	up	homo sapiens protocadherin 7 transcript variant mrna
Ssa#S26387025	S26387025	2.6	up	cathelicidin antimicrobial peptide
Ssa#S35477274_S	S35477274	2.6	up	---NA---
Ssa#S35470708	S35470708	2.6	up	wu:fc83f10
Omy#S30132072	S30132072	2.6	up	chemokine receptor-like protein 1
Ssa#STIR02561	gi|209736883	2.6	up	lymphocyte g0 g1 switch protein 2
Omy#BX307803	BX307803	2.6	up	Ell2 protein
Ssa#STIR20800	TC82233	2.6	up	sequestosome 1
Ssa#TC105320	TC105320	2.6	up	Nuclear factor NF-kappa-B p100 subunit
Omy#TC166897	TC166897	2.6	up	Suppression of tumorigenicity 5
Omy#NP544427	NP544427	2.6	up	rapd fragment a19g19a-01
Ssa#S22712055	S22712055	2.6	up	NF-kappa-B inhibitor epsilon
Ssa#DN162389	DN162389	2.6	up	LIM domain only 7a
Ssa#S30240713	S30240713	2.6	up	TRAF interacting protein TANK
Ssa#STIR14784	TC73371	2.6	up	---NA---
Ssa#CL510Ctg1	CL510Contig1	2.6	up	neutrophil cytosolic factor 1
Ssa#S30242761	DW538822	2.6	up	glycolipid transfer protein
Ssa#TC99766	TC99766	2.6	up	similar to proton/amino acid transporter 4
Ssa#S31981521	S31981521	2.6	up	Probable E3 ubiquitin-protein ligase HERC4
Ssa#EG832032	EG832032	2.6	up	homo sapiens atp-binding sub-family c (cftr mrp) member 1 transcript variant mrna
Ssa#DW550996	DW550996	2.6	up	UDP-xylose and UDP-N-acetylglucosamine transporter
Ssa#STIR17210	TC76880	2.6	up	serine or cysteine proteinase inhibitor clade e member 1
Ssa#CL420Ctg1	CL420Contig1	2.6	up	5-aminolevulinate synthase, nonspecific, mitochondrial precursor
Ssa#STIR02141	gi|209737735	2.6	up	protein tyrosine phosphatase 4a2
Ssa#S32011572	S32011572	2.6	up	salmo salar clone ssal-rgf-002-348 collagen alpha-3vi chain precursor pseudogene cds
Ssa#STIR11644	TC68934	2.6	up	spastic ataxia of charlevoix-saguenay
Ssa#S35693508	S35693508	2.6	up	solute carrier family 37 member 1
Ssa#STIR11473	TC68707	2.6	up	nuclear factor interleukin-3-regulated protein
Ssa#STIR16962	TC76501	2.6	up	kda protein
Ssa#S35690902	S35690902	2.6	up	ARMET precursor
Omy#S18094456	S18094456	2.6	up	: hm:gc12
Ssa#STIR00154_2	SSU66475	2.6	up	interferon inducible mx protein
Ssa#KSS3086	KSS3086	2.6	up	transposase
Ssa#STIR02327	gi|209737361	2.6	up	h+lysosomal v1 subunit a
Ssa#S31984274	S31984274	2.6	up	danio rerio phosphoinositide-3-kinase adaptor protein 1 mrna
Ssa#STIR00057_3	DW568723	2.6	up	interferon regulatory factor 1
Ssa#S35669786	S35669786	2.6	up	Poly polymerase 11
Ssa#DW566454	DW566454	2.6	up	signal transducer and activator of transcription 2
Ssa#S48418520	S48418520	2.6	up	Transmembrane protein 71
Ssa#S48431263	S48431263	2.6	up	Tax1-binding protein 1 homolog
Ssa#STIR00087_2	DW555246	2.6	up	interleukin 10beta
Ssa#CL224Ctg1	CL224Contig1	2.6	up	Receptor-interacting serine/threonine-protein kinase 4
Ssa#KSS4945	KSS4945	2.6	up	Tax1-binding protein 1 homolog
Ssa#S35543442	S35543442	2.6	up	Probable E3 ubiquitin-protein ligase RNF144A-A
Ssa#S31978752	S31978752	2.6	up	Vacuolar ATP synthase catalytic subunit A
Ssa#STIR12230	TC69738	2.6	up	Lithognathus mormyrus clone lithmor1223 mRNA sequence

Ssa#S30292938	S30292938	2.6	up	novel protein similar to H.sapiens PTPN18, protein tyrosine phosphatase, non-receptor type 18
Ssa#STIR10715	TC67665	2.5	up	cd83
Ssa#S22710961	S22710961	2.5	up	hypothetical protein LOC100148091
Ssa#DY699301	DY699301	2.5	up	chaenocephalus aceratus bloodthirsty
Ssa#STIR08606	TC64968	2.5	up	kda protein
Ssa#DY733521	DY733521	2.5	up	Tax1-binding protein 1 homolog
Ssa#S18836987	S18836987	2.5	up	Ubiquitin-like protein
Ssa#S30282625	S30282625	2.5	up	G protein-coupled receptor 155
Ssa#STIR00154_3	SSU66475	2.5	up	interferon inducible mx protein
Ssa#EG811212	EG811212	2.5	up	hypothetical protein BRAFLDRAFT_123644
Ssa#TC88585	TC88585	2.5	up	sus scrofa clone: expressed in periphral blood mononuclear cell
Ssa#STIR28301	TC93562	2.5	up	SJCHGC03026 protein
Ssa#BG935149	BG935149	2.5	up	ATP synthase F0 subunit 6
Ssa#KSS214	KSS214	2.5	up	Apolipoprotein-L3
Ssa#TC93909	TC93909	2.5	up	Angiopoietin-related protein 4
Ssa#STIR16778	TC76229	2.5	up	dna-binding protein inhibitor id-1
Ssa#KSS1762	KSS1762	2.5	up	novel protein
Ssa#STIR06994	gi|209153985	2.5	up	ets variant 6
Ssa#CX356211	CX356211	2.5	up	Stress 70 protein chaperone microsome-associated 60 kDa protein precursor
Ssa#KSS4577	KSS4577	2.5	up	hypothetical protein
Ssa#KSS4410	KSS4410	2.5	up	Lysozyme g
Ssa#STIR02714	gi|209736577	2.5	up	multiple banded antigen
Ssa#TC106530	TC106530	2.5	up	similar to Kv3.3 potassium channel subunit
Ssa#DW582880	DW582880	2.5	up	similar to endoplasmic reticulum to nucleus signalling 1
Ssa#STIR17317	TC77030	2.5	up	nuclear factor of kappa light polypeptide gene enhancer in b-cellsepsilon
Ssa#S18892316	S18892316	2.5	up	myxovirus resistance 2
Ssa#S35559302	S35559302	2.5	up	Pituitary tumor-transforming gene 1 protein-interacting protein precursor
Ssa#STIR25722	TC89665	2.5	up	novel proteinvertebrate galectins
Ssa#S30259195	S30259195	2.5	up	Cytochrome P450 7B1
Ssa#STIR20983	TC82519	2.5	up	Oncorhynchus mykiss microsatellite OMM1095 sequence
Ssa#S30263129	S30263129	2.5	up	ceruloplasmin
Con_CANDS_06	SSU66475	2.5	up	S salar Mx1 protein mRNA
Ssa#S35486979	S35486979	2.5	up	Cell death activator CIDE-3
Ssa#S35594657	S35594657	2.5	up	hypothetical protein LOC100148091
Ssa#S35581771	S35581771	2.5	up	Lysozyme g
Ssa#S35664619	S35664619	2.5	up	glutaminyl-peptide cyclotransferase-like
Ssa#TC67613	TC67613	2.5	up	Tyrosine-protein phosphatase non-receptor type 7
Ssa#STIR22553	TC84903	2.5	up	Osmerus mordax clone omor-eva-507-053 ADP-ribosylation factor-like protein 5B putative mRNA,
Ssa#S30277391	S30277391	2.5	up	hypothetical protein LOC436731
Ssa#S35559267	S35559267	2.5	up	Tyrosine-protein kinase SRK2
Ssa#S35532410	S35532410	2.5	up	Ras-related protein Rab-27B
Ssa#S35477273	S35477273	2.5	up	---NA---
Ssa#EG920909	EG920909	2.5	up	similar to PPAR-alpha interacting complex protein 285
Ssa#STIR21614	TC83491	2.5	up	---NA---
Ssa#S35677866	S35677866	2.5	up	Cation-dependent mannose-6-phosphate receptor
Ssa#STIR17101	TC76712	2.5	up	heat shock 70kda protein 5 (glucose-regulated78kda)
Ssa#S35681202	S35681202	2.5	up	F5 protein
Ssa#S30235521	S30235521	2.5	up	rattus norvegicus la ribonucleoprotein domain member 1 mrna
Ssa#S35582377	S35582377	2.5	up	Calsequestrin-1
Omy#CA352451	CA352451	2.5	up	transposase
Ssa#EG831762	EG831762	2.5	up	salmo salar adipocyte plasma membrane-associated protein mrna
Ssa#S35536016	S35536016	2.5	up	Vesicle-associated membrane protein 8
Ssa#STIR15506	TC74369	2.5	up	sequestosome 1
Ssa#S31981688_S	S31981688	2.5	up	Peroxisomal proliferator-activated receptor A-interacting complex 285 kDa protein
Ssa#TC112901	TC112901	2.5	up	non-LTR retrotransposable element partially supported by GENSCAN in Oryzias latipes
Ssa#TC95429	TC95429	2.5	up	v-maf musculoaponeurotic fibrosarcoma oncogene-like
Ssa#KSS4194	KSS4194	2.5	up	Homocysteine-responsive endoplasmic reticulum-resident ubiquitin-like domain member 1 protein
Ssa#STIR03534	gi|209734931	2.5	up	kda protein
Ssa#KSS4994	KSS4994	2.5	up	similar to Leukotriene B4 receptor 1
Ssa#STIR20191	TC81336	2.5	up	---NA---
Ssa#S30290522	S30290522	2.5	up	T-cell activation Rho GTPase-activating protein
Omy#S15323125	S15323125	2.5	up	Sequestosome-1
Ssa#STIR20900	TC82376	2.5	up	phosphatidylinositol glycan anchorclass h
Ssa#DY739459	DY739459	2.5	up	angiopoietin-like 2 isoform 1
Ssa#STIR14524	TC72992	2.5	up	asteroid homolog 1
Ssa#TC93419	TC93419	2.5	up	Type-2 angiotensin II receptor
Ssa#S35555091	S35555091	2.5	up	Probable E3 ubiquitin-protein ligase RNF144A-A
Ssa#S35586274	S35586274	2.5	up	C-ets-2
Ssa#STIR03863	gi|209734273	2.5	up	kda protein
Ssa#STIR16344	TC75569	2.5	up	pre-B cell enhancing factor
Ssa#CA040629	CA040629	2.5	up	NEDD4-like E3 ubiquitin-protein ligase WWP2
Ssa#STIR02171	gi|209737673	2.5	up	ccaat enhancer-binding protein delta
Ssa#CB515386	CB515386	2.5	up	similar to development and differentiation enhancing factor 1 isoform 1
Ssa#S30289618	S30289618	2.4	up	matrix metalloproteinase 9
Ssa#S30285352	S30285352	2.4	up	tetraodon nigroviridis full-length cdna
Ssa#KSS3245	KSS3245	2.4	up	Ubiquitin-like protein
Omy#TC160926	TC160926	2.4	up	similar to dual specificity phosphatase 26
Ssa#S31990843	S31990843	2.4	up	tetraodon nigroviridis full-length cdna
Ssa#STIR05787	gi|209730401	2.4	up	lymphocyte g0 g1 switch protein 2
Ssa#STIR12737	TC70440	2.4	up	gene model
Ssa#STIR04513	gi|209732973	2.4	up	kda protein
Ssa#KSSb2332	KSSb2332	2.4	up	Transcription factor jun-B
Ssa#STIR24241	TC87437	2.4	up	---NA---
Ssa#S30242375	S30242375	2.4	up	Suppression of tumorigenicity 5
Ssa#TC86798	TC86798	2.4	up	similar to B aggressive lymphoma long
Omy#CA381502	CA381502	2.4	up	rattus norvegicus mucolipin 3 mrna
Ssa#STIR15760	TC74739	2.4	up	g protein-coupled receptor 137
Ssa#TC106357	TC106357	2.4	up	similar to 1,2-alpha-mannosidase IC
Omy#S15294190	S15294190	2.4	up	: phospholipase A2, group IVA (cytosolic, calcium-dependent)
Ssa#S30276608	S30276608	2.4	up	Legumain precursor
Omy#S20792283	S20792283	2.4	up	vascular endothelial growth factor precursor
Ssa#S31971283	DY700073	2.4	up	cell division cycle associated 4
Ssa#CL201Ctg1	CL201Contig1	2.4	up	Caspase-3
Ssa#TC111170	TC111170	2.4	up	hypothetical protein LOC327345
Ssa#STIR19452	TC80218	2.4	up	Salmo salar clone ssal-rgh-520-198 Metalloproteinase inhibitor 2 precursor putative mRNA,
Omy#CU069688	CU069688	2.4	up	DeltaC

Ssa#STIR43211	DY716967	2.4	up	similar to Vesicle transport protein SEC20 isoform 1
Ssa#S32007250	S32007250	2.4	up	Adipophilin
Omy#CA347902	CA347902	2.4	up	Splicing factor, arginine/serine-rich 4
Ssa#STIR21932	TC83965	2.4	up	---NA---
Ssa#STIR11534	TC68790	2.4	up	interferon-induced protein 44-like
Ssa#S35547363	S35547363	2.4	up	cytochrome b558 alpha-subunit
Ssa#S30294618	DW581582	2.4	up	cyclin D1
Ssa#STIR24851	TC88354	2.4	up	glycogen phosphorylase
Ssa#DW549168	DW549168	2.4	up	danio rerio serine incorporator mrna (cdna clone mgc:192824 image:100061299)
Ssa#STIR14785	TC73373	2.4	up	sorting nexin 14
Ssa#STIR19198	TC79816	2.4	up	Oncorhynchus tshawytscha clone ChinookGH2 growth hormone 2 gene
Omy#S15299642	S15299642	2.4	up	core 1 synthase, glycoprotein-N-acetylgalactosamine 3-beta-galactosyltransferase, 1b
Ssa#S31967511_S	S31967511	2.4	up	unnamed protein product
Ssa#S35685793	S35685793	2.4	up	similar to myelin associated glycoprotein
Ssa#STIR14052	TC72313	2.4	up	---NA---
Ssa#TC71742	TC71742	2.4	up	strongylocentrotus purpuratus high affinity choline transporter mrna
Ssa#S35549782	S35549782	2.4	up	Ubiquitin-like protein precursor
Ssa#S30271445	S30271445	2.4	up	: B cell RAG associated protein
Ssa#STIR23344	TC86103	2.4	up	very acidic salivary protein
Ssa#STIR09767	TC66431	2.4	up	ets variant gene 6 (tel oncogene)
Omy#CA382209	CA382209	2.4	up	danio rerio olfactomedin 2 mrna
Ssa#S35594682	S35594682	2.4	up	danio rerio plexin d1
Ssa#S48427530	S48427530	2.4	up	tetraodon nigroviridis full-length cdna
Ssa#S35555077	S35555077	2.4	up	similar to mCG1046517
Omy#S19712894	S19712894	2.4	up	homo sapiens zinc fyve domain containing 1 transcript variant mrna
Ssa#STIR19395	TC80126	2.4	up	5-aminolevulinate synthase 1
Ssa#S35562259	S35562259	2.4	up	Apoptosis-associated speck-like protein containing a CARD
Ssa#S35559601	S35559601	2.4	up	hypothetical protein
Ssa#DY699550	DY699550	2.4	up	transposase
Ssa#S35581770	S35581770	2.4	up	Lysozyme g
Ssa#CB513260	CB513260	2.4	up	hypothetical protein LOC571403
Ssa#STIR00110_3	BM413877	2.4	up	adipose differentiation-related protein
Ssa#KSS4521	KSS4521	2.4	up	salmo salar clone ssal-rgf-512-220 macrosialin precursor
Ssa#S31991729	S31991729	2.4	up	similar to thymic stromal cotransporter
Ssa#CL300Ctg1	CL300Contig1	2.4	up	Proteasome subunit beta type-9-B like protein
Omy#S18092378	S18092378	2.4	up	novel protein similar to vertebrate coenzyme Q4 homolog (S. cerevisiae) (COQ4)
Ssa#STIR20203	TC81355	2.4	up	hypothetical loc792613
Ssa#S35494312	S35494312	2.4	up	Zinc finger protein SLUG
Ssa#S30239782	S30239782	2.4	up	G protein-coupled receptor 137bb
Ssa#TC65444	TC65444	2.4	up	tetraodon nigroviridis full-length cdna
Ssa#S35574286	S35574286	2.3	up	Interferon-induced GTP-binding protein Mx
Ssa#STIR24044	TC87162	2.3	up	Salmo salar clone ssal-rgf-534-241 NF-kappa-B inhibitor epsilon putative mRNA,
Ssa#S32007633	S32007633	2.3	up	C16orf14 homolog
Ssa#S35590616	S35590616	2.3	up	hydroxyprostaglandin dehydrogenase 15-(NAD)
Ssa#S30241432	S30241432	2.3	up	hypothetical protein LOC100141487
Omy#BX081765	BX081765	2.3	up	zebrafish dna sequence from clone dkey-228g21 in linkage group complete sequence
Ssa#STIR17713	TC77606	2.3	up	erythrocyte membrane protein band
Ssa#S35671378	S35671378	2.3	up	rattus norvegicus sacsin mrna
Ssa#EG833198	EG833198	2.3	up	Adipocyte plasma membrane-associated protein
Ssa#STIR14530	TC73000	2.3	up	g protein-coupled receptor 132
Ssa#CX357575	CX357575	2.3	up	hypothetical protein LOC402960
Ssa#KSS4965	KSS4965	2.3	up	proteasome subunit beta type 7b
Ssa#STIR03295	gi|209735411	2.3	up	kda protein
Ssa#STIR22314	TC84530	2.3	up	Salmo salar clone ssal-rgf-524-264 unknown large open reading frame mRNA, novel cds
Ssa#STIR00067_4	U66477	2.3	up	interferon inducible mx protein
Ssa#TC110963	TC110963	2.3	up	GTP-binding protein 1
Ssa#S30279916	S30279916	2.3	up	novel protein similar to vertebrate phosphoglycerate mutase family member 4 (PGAM4, zgc:63722)
Ssa#CB516074	CB516074	2.3	up	similar to dipeptidase 3
Ssa#S30284825	S30284825	2.3	up	Dehydrogenase/reductase SDR family member 13 precursor
Ssa#CX354422	CX354422	2.3	up	pre-B-cell colony-enhancing factor
Con_CANDS_07	SSU66476	2.3	up	S salar Mx2 protein mRNA
Ssa#STIR16259	TC75448	2.3	up	yy1 transcription factor
Ssa#STIR00027_4	DQ008069	2.3	up	---NA---
Omy#gi185132491	gi185132491	2.3	up	Oncorhynchus mykiss VHSV-induced protein-5 (LOC100135997), mRNA
Ssa#S32012431	DY741028	2.3	up	StAR-related lipid transfer protein 3
Ssa#STIR00057_4	DW568723	2.3	up	interferon regulatory factor 1
Ssa#STIR25663	TC89576	2.3	up	heat shock protein 8
Ssa#TC103631	TC103631	2.3	up	Transmembrane protein C1orf78 homolog
Ssa#TC101698	TC101698	2.3	up	AF465280_1coagulation factor V precursor
Ssa#S31974262	S31974262	2.3	up	Damage-regulated autophagy modulator
Ssa#TC94547	TC94547	2.3	up	serum/glucocorticoid regulated kinase 3
Ssa#STIR13769	TC71910	2.3	up	Protein disulfide isomerase associated 4
Ssa#S35531339	S35531339	2.3	up	macaca mulatta lysyl oxidase-like transcript variant 3 mrna
Ssa#STIR20840	TC82288	2.3	up	synovialx breakpoint 2 interacting protein
Ssa#CL509Ctg1	CL509Contig1	2.3	up	ARMET precursor
Ssa#KSSb2709	KSSb2709	2.3	up	RNA-binding protein 24-B
Ssa#S35597250	S35597250	2.3	up	caspase-9
Ssa#STIR15489	TC74347	2.3	up	Zebrafish DNA sequence from clone DKEY-222K23 in linkage group 21, complete sequence
Ssa#STIR22403	TC84672	2.3	up	nuclear factor of kappa light polypeptide gene enhancer in b-cellsepsilon
Ssa#S31991804	S31991804	2.3	up	interferon-inducible protein Gig2-like
Ssa#STIR09459	TC66044	2.3	up	family with sequence similaritymember a
Ssa#STIR24941	TC88487	2.3	up	Danio rerio zgc:114107, mRNA (cDNA clone MGC:114107 IMAGE:7448987),
Omy#gi20270898	gi20270898	2.3	up	Oncorhynchus mykiss VHSV-induced protein-7 mRNA,
Ssa#STIR18043	TC78105	2.3	up	---NA---
Ssa#STIR19148	TC79744	2.3	up	mgc79118 protein
Ssa#S19102665	S19102665	2.3	up	Ras-related protein Rab-3D
Ssa#EG914592_S	EG914592	2.3	up	transposase
Ssa#S31984359	S31984359	2.3	up	Calcium/calmodulin-dependent protein kinase type 1
Ssa#S31980174_S	S31980174	2.3	up	Matrix-remodeling-associated protein 8 precursor
Ssa#S32008049	S32008049	2.3	up	Interferon-induced protein 44
Ssa#STIR12117	TC69580	2.3	up	Transcription factor jun-B
Ssa#STIR17354	TC77088	2.3	up	Salmo salar clone ssal-rgf-516-020 Translocation-associated membrane protein 1-like 1
Ssa#CL50Contig2	CL50Contig2	2.3	up	Fructose-bisphosphate aldolase A
Ssa#S35569866	S35569866	2.3	up	KIAA0174 homolog
Ssa#CB499612	CB499612	2.3	up	activating transcription factor 6
Ssa#CL185Ctg1	CL185Contig1	2.3	up	cytochrome b-245, beta polypeptide

Ssa#TC104138	TC104138		2.3	up	danio rerio im:7157189 (im:7157189) mrna
Ssa#S35523686	S35523686		2.3	up	smoothelin, like
Ssa#S35553414	S35553414		2.3	up	Serine/threonine-protein phosphatase 2B catalytic subunit alpha isoform
Ssa#S30242582	S30242582		2.3	up	78 kDa glucose-regulated protein
Ssa#CX356561	CX356561		2.3	up	lysyl hydroxylase 2
Omy#CA352575	CA352575		2.3	up	similar to B aggressive lymphoma long
Ssa#KSS3977_S	KSS3977		2.3	up	similar to NEDD4 binding protein 2-like 1
Ssa#STIR24729_S	TC88167		2.3	up	similar to zinc finger protein 227
Ssa#STIR20698	TC82078		2.3	up	glutaminase kidneymitochondrial precursor(l-glutamine amidohydrolase) (k-glutaminase)
Ssa#STIR07654	TC63835		2.3	up	---NA---
Ssa#tpSSTN_BARB		0	2.3	up	salartransposon_SSTN_BARB
Ssa#STIR17281	TC76974		2.3	up	tsku protein
Ssa#DY728444	DY728444		2.3	up	Integrin, alpha V
Ssa#S30295519	S30295519		2.3	up	Probable ATP-dependent RNA helicase DHX58
Ssa#DY723182	DY723182		2.3	up	---NA---
Ssa#S48394116_S	S48394116		2.3	up	pellino protein
Ssa#S35472384	S35472384		2.3	up	sortin nexin member
Ssa#S35552102	S35552102		2.3	up	DnaJ-like subfamily B member 11
Ssa#S30294878	S30294878		2.3	up	hypothetical protein LOC549553
Ssa#STIR14689	TC73235		2.2	up	heat shock protein 70
Ssa#STIR04015	BT048053		2.2	up	proteasomebeta type 8
Ssa#STIR19413	TC80156		2.2	up	---NA---
Ssa#S30239684	S30239684		2.2	up	cytochrome b-245, beta polypeptide
Ssa#DW544960	DW544960		2.2	up	Intraflagellar transport 20 homolog
Ssa#S35584663	S35584663		2.2	up	mab-21-like 1
Omy#S34309291	S34309291		2.2	up	novel protein similar to vertebrate hect domain and RLD 3 (HERC3)
Ssa#S35676149	S35676149		2.2	up	Cysteine-rich protein 1
Ssa#DY720811	DY720811		2.2	up	xenopus tropicalis ring finger protein 11 mrna
Ssa#S48419676	S48419676		2.2	up	F-box/LRR-repeat protein 5
Ssa#TC71753_S	TC71753		2.2	up	FAM114A2
Ssa#STIR04367	gi|209733265		2.2	up	chemokine (c-c motif) ligand 19
Ssa#STIR40534	TC111752		2.2	up	lipoprotein lipase
Ssa#S30239539	S30239539		2.2	up	Alpha-galactosidase A precursor
Ssa#STIR24555	TC87903		2.2	up	---NA---
Ssa#S35529611	S35529611		2.2	up	BTB (POZ) domain containing 10a
Ssa#S30295323	DW582287		2.2	up	Proteasome subunit alpha type-6
Ssa#S31993707	S31993707		2.2	up	Heparin-binding EGF-like growth factor precursor
Ssa#S30296847	S30296847		2.2	up	novel protein similar to H.sapiens PRKX, protein kinase, X-linked (PRKX)
Ssa#S48404486	S48404486		2.2	up	Translocation-associated membrane protein 1-like 1
Omy#S18534184	S18534184		2.2	up	similar to MGC80104 protein, partial
Ssa#CL189Ctg1	CL189Contig1		2.2	up	PLAC8-like protein 1
Ssa#STIR14783	TC73370		2.2	up	rna binding motif protein 24
Ssa#STIR17899	TC77882		2.2	up	---NA---
Ssa#STIR15045_S	TC73724		2.2	up	activating transcription factor 6
Ssa#S18875066	S18875066		2.2	up	Twisted gastrulation protein homolog 1-A
Ssa#STIR15343	TC74150		2.2	up	---NA---
Ssa#STIR20418	TC81675		2.2	up	ras-related protein rab-11a
Ssa#S30258568	S30258568		2.2	up	interleukin-1 receptor-associated kinase 4
Ssa#S30242512	S30242512		2.2	up	similar to Leukotriene B4 receptor 1
Ssa#NP12953959	NP12953959		2.2	up	diacylglycerol O-acyltransferase-like protein 1
Omy#CA360702	CA360702		2.2	up	S-adenosylmethionine synthetase isoform type-2
Ssa#S30270545	S30270545		2.2	up	homo sapiens genomic chromosome 11q clone:rp11- complete sequences
Ssa#AM402542	AM402542		2.2	up	vertebrate type 1 tumor necrosis factor receptor shedding aminopeptidase regulator (ARTS-1)
Ssa#S30283965	S30283965		2.2	up	similar to RING finger protein 122
Ssa#S35582636	S35582636		2.2	up	Actin-related protein 2/3 complex subunit 5
Ssa#S32007571	S32007571		2.2	up	Syntaxin-binding protein 2
Omy#CA373578	CA373578		2.2	up	RNA-binding motif, single-stranded-interacting protein 1
Ssa#S31987442	S31987442		2.2	up	Si:ch211-197g15.7 protein
Ssa#S31978703	S31978703		2.2	up	component of oligomeric golgi complex 2
Ssa#KSS1939	KSS1939		2.2	up	AF483541_1VHSV-induced protein
Ssa#KSSb2629	KSSb2629		2.2	up	helicase MOV-10
Ssa#S31977582	S31977582		2.2	up	similar to Ras association domain-containing protein 8 (Carcinoma-associated protein HOJ-1)
Ssa#S30262545	S30262545		2.2	up	Prolyl endopeptidase
Ssa#S35538062_S	S35538062		2.2	up	monoacylglycerol O-acyltransferase 1
Ssa#S35599664	S35599664		2.2	up	gallus gallus protein phosphatase regulatory subunit 3c mrna
Ssa#S35482017	S35482017		2.2	up	: 6-phosphofructo-2-kinase/fructose-2,6-biphosphatase 1
Ssa#S48394398_S	S48394398		2.2	up	tetraspanin 13
Ssa#S48372410_S	S48372410		2.2	up	unnamed protein product
Ssa#TC111637	TC111637		2.2	up	lysyl hydroxylase 2
Ssa#STIR18432	TC78682		2.2	up	Salmo salar clone ssal-rgf-519-251 Apolipoprotein-L3 putative mRNA,
Ssa#STIR17975	TC77996		2.2	up	---NA---
Ssa#CL366Ctg1	CL366Contig1		2.2	up	Tumor necrosis factor receptor superfamily member 5
Ssa#STIR12525	TC70144		2.2	up	---NA---
Ssa#KSS4206	KSS4206		2.2	up	similar to poly (ADP-ribose) polymerase family, member 14
Ssa#S34550848	S34550848		2.2	up	opioid growth factor receptor
Ssa#S48420713	S48420713		2.2	up	Interferon regulatory factor 3
Omy#S32321336	S32321336		2.2	up	inwardly-rectifying channel, subfamily J, member 2
Ssa#STIR25451	TC89255		2.2	up	metalloproteinase inhibitor 2 precursor
Ssa#CL509Ctg1_S	CL509Contig1		2.2	up	ARMET precursor
Ssa#STIR42904	EG758722		2.2	up	Cytochrome b5
Ssa#S31964109	S31964109		2.2	up	XIAP-associated factor 1
Ssa#S48399056	S48399056		2.2	up	Salmo salar clone ssal-rgf-511-070 unknown large open reading frame mRNA, novel cds
Ssa#CK885821	CK885821		2.2	up	salmo salar bifunctional 3-phosphoadenosine 5-phosphosulfate synthetase 2 mrna
Ssa#DY734032	DY734032		2.2	up	I50116N-cadherin precursor - zebra fish
Ssa#S35678943	S35678943		2.2	up	Signal transducer and activator of transcription 1
Ssa#STIR11723	TC69043		2.2	up	ccaat enhancer-binding protein delta
Ssa#STIR00104_4	CK874054		2.2	up	septin 9
Ssa#STIR17286	TC76983		2.2	up	traf-type zinc finger domain containing 1
Ssa#S35549130	EG835508		2.2	up	cyclin G2
Ssa#S35568707_S	S35568707		2.2	up	DnaJ (Hsp40) homolog, subfamily B, member 9
Ssa#STIR11466	TC68697		2.2	up	deltex 3-like
Ssa#TC104626_S	TC104626		2.2	up	unnamed protein product
Ssa#S35551941	S35551941		2.2	up	Arrestin domain-containing protein 2
Ssa#S29048819	S29048819		2.2	up	Fructose-bisphosphate aldolase A
Ssa#KSS621	KSS621		2.2	up	Tapasin precursor
Ssa#S30281676	S30281676		2.2	up	danio rerio zgc:112183 (zgc:112183) mrna

Ssa#STIR23701	TC86643	2.2	up	---NA---
Ssa#S30293409	S30293409	2.2	up	similar to interferon-inducible protein Gig1
Ssa#STIR17126	TC76745	2.2	up	Salmo salar clone ssal-rgf-526-361 Suppressor of IKK-epsilon putative mRNA,
Ssa#S30280974	S30280974	2.2	up	Probable ATP-dependent RNA helicase DHX58
Omy#S34424631	S34424631	2.2	up	similar to SH2B adapter protein 2 (SH2 and PH domain-containing adapter protein APS)
Ssa#STIR25693	TC89621	2.2	up	switch-associated protein 70
Ssa#STIR18760	TC79175	2.2	up	15-hydroxyprostaglandin dehydrogenase
Ssa#KSS4158	KSS4158	2.2	up	ubiquitin (ribosomal protein L40), putative
Ssa#KSS618_S	KSS618	2.2	up	novel protein (zgc:153654)
Ssa#TC99504	TC99504	2.2	up	Cation-dependent mannose-6-phosphate receptor
Ssa#TC97908	TC97908	2.2	up	danio rerio caspase recruitment domain member 11 mrna
Ssa#STIR25195	TC88869	2.2	up	---NA---
Ssa#S30291889	S30291889	2.2	up	Ras-related and estrogen-regulated growth inhibitor
Ssa#STIR16064	TC75161	2.2	up	6-phosphofructo-2-kinase/fructose-2,6-biphosphatase 4
Ssa#S35685952	S35685952	2.2	up	Deoxycytidine kinase
Ssa#S30295133	S30295133	2.2	up	: novel protein similar to mucosa associated lymphoid tissue lymphoma translocation gene 1 (malt1)
Ssa#STIR03375	gi|209735251	2.2	up	digestive-organ expansion factor
Ssa#STIR10385	TC67231	2.1	up	vig-2 protein
Ssa#DW546415	DW546415	2.1	up	S-adenosylmethionine synthetase isoform type-2
Ssa#STIR10480	TC67353	2.1	up	pdz and lim domain 1
Omy#CA351852	CA351852	2.1	up	Type-2 angiotensin II receptor
Omy#CA374893	CA374893	2.1	up	danio rerio zgc:65781 (zgc:65781) mrna
Ssa#S30281943	S30281943	2.1	up	Transcription factor jun-B
Ssa#KSS1565	KSS1565	2.1	up	Proteasome subunit alpha type-6
Ssa#STIR16669	TC76058	2.1	up	cd209 antigen-like protein d
Omy#S15279041	S15279041	2.1	up	hypothetical protein LOC327345
Ssa#STIR18500	TC78791	2.1	up	poly (adp-ribose) polymerasemember 11
Omy#S34424804	S34424804	2.1	up	heteropneustes fossilis wdr13 protein complete alternatively spliced
Omy#TC139878	TC139878	2.1	up	danio rerio nuclear prelamin a recognition factor mrna
Ssa#S35591225	S35591225	2.1	up	FXYD domain containing ion transport regulator 5b
Ssa#S30269828	S30269828	2.1	up	similar to interferon-induced protein 44-like
Ssa#KSS4744	KSS4744	2.1	up	mRNA-decapping enzyme 2
Ssa#STIR22513	TC84848	2.1	up	phospholipase dmember 3
Omy#gi185132669	gi185132669	2.1	up	Oncorhynchus mykiss VHSV-induced protein-9 (lgals9), mRNA
Ssa#S18887549	S18887549	2.1	up	K+ channel tetramerization protein
Ssa#S35545968	S35545968	2.1	up	Cysteine-rich protein 1
Ssa#KSS816	KSS816	2.1	up	HLA class II histocompatibility antigen gamma chain
Ssa#STIR26129	TC90272	2.1	up	atph+ transporting mitochondrial f1beta subunit
Ssa#S31987661	S31987661	2.1	up	Sodium/glucose cotransporter 2
Ssa#TC102663	TC102663	2.1	up	similar to ubiquitin-activating enzyme E1, partial
Ssa#STIR17381	TC77139	2.1	up	---NA---
Omy#CA388200	CA388200	2.1	up	similar to microtubule-associated protein 1 A
Ssa#STIR07231	gi|209149706	2.1	up	myeloid cell leukemia sequence 1 (bcl2-related)
Ssa#STIR20522	TC81813	2.1	up	galectin like protein
Ssa#TC87300	TC87300	2.1	up	solute carrier family 35, member A2
Ssa#EG647908	EG647908	2.1	up	dihydropyrimidinase-like 2
Ssa#S30283313	S30283313	2.1	up	nicotinic acetylcholine receptor beta 7 subunit
Ssa#DW006091	DW006091	2.1	up	AF504024_1MHC class I
Ssa#KSS3463	KSS3463	2.1	up	Cation-dependent mannose-6-phosphate receptor
Ssa#S31992822	S31992822	2.1	up	Alba-like protein C9orf23
Ssa#S30274770	S30274770	2.1	up	Similar to Tetraodon protein product CAG00085
Ssa#S35569655	S35569655	2.1	up	Digestive organ expansion factor
Ssa#TC65895	TC65895	2.1	up	novel zinc finger protein
Ssa#STIR18597	TC78936	2.1	up	anti-apoptotic protein nr13
Ssa#S35561456	S35561456	2.1	up	tetraodon nigroviridis full-length cdna
Ssa#S35687702	S35687702	2.1	up	Ras-related GTP binding C
Ssa#CK897847_S	CK897847	2.1	up	similar to pol polyprotein
Ssa#S35567746	S35567746	2.1	up	: si:ch211-207m11.1
Ssa#STIR17644	TC77512	2.1	up	tripartite motif-containing 39
Ssa#TC92817_S	TC92817	2.1	up	methyl-CpG binding domain protein 2
Ssa#S35663915	S35663915	2.1	up	RanBP-type and C3HC4-type zinc finger-containing protein 1
Ssa#S31978702	S31978702	2.1	up	similar to interferon-induced protein 44-like
Ssa#KSS2990	KSS2990	2.1	up	tetraodon nigroviridis full-length cdna
Ssa#TC69065	TC69065	2.1	up	Signal transducer and activator of transcription 1
Ssa#DW553532	DW553532	2.1	up	6-phosphofructo-2-kinase
Omy#S19712893	S19712893	2.1	up	similar to chloride channel CLC-5
Ssa#S35570074	S35570074	2.1	up	Ras-related GTP-binding protein C
Ssa#STIR00063_2	DW537370	2.1	up	interferon regulatory factor 8
Ssa#STIR09349	TC65907	2.1	up	h-2 class ii histocompatibility antigen gamma chain
Ssa#S30265122	S30265122	2.1	up	danio rerio phosphatidylinositol transfer cytoplasmic 1 partial mrna
Ssa#STIR06452	gi|209155081	2.1	up	proline-rich nuclear receptor coactivator 2
Ssa#STIR07334	gi|209147284	2.1	up	ras (rad and gem)-like gtp-binding 1
Ssa#S35550152	S35550152	2.1	up	transmembrane protein 41B
Ssa#STIR01500	gi|197632104	2.1	up	fk506 binding protein 1b
Omy#S15301725	S15301725	2.1	up	UDP-glucose 4-epimerase
Ssa#STIR10312	TC67130	2.1	up	calcium binding and coiled-coil domain 2
Ssa#STIR12657	TC70332	2.1	up	methionine adenosyltransferasealpha
Ssa#TC105009	TC105009	2.1	up	G-protein-coupled receptor GPR34 type 2
Ssa#STIR04422	gi|209733155	2.1	up	proteasome subunit beta type-6 precursor
Ssa#S35559076	S35559076	2.1	up	Angiopoietin-related protein 4
Ssa#STIR03912	gi|209734175	2.1	up	kda protein
Ssa#KSS1286	KSS1286	2.1	up	similar to MOB1, Mps One Binder kinase activator-like 2B
Ssa#S35702407	S35702407	2.1	up	similar to B aggressive lymphoma long
Omy#S34424403	S34424403	2.1	up	Syntaxin-binding protein 2
Ssa#STIR15323	TC74120	2.1	up	swim zinc finger protein domain-containing
Ssa#STIR23616	TC86511	2.1	up	wd repeat domain 52
Omy#S18157537	BX883008	2.1	up	Interleukin-6 receptor subunit alpha precursor
Ssa#CL372Ctg1	CL372Contig1	2.1	up	Alpha-enolase
Ssa#STIR09282	TC65819	2.1	up	wd repeat domain 13
Ssa#TC87519	TC87519	2.1	up	tribbles 3
Ssa#S35684862	S35684862	2.1	up	homo sapiens diacylglycerol o-acyltransferase homolog 1 mrna
Ssa#S30269655	S30269655	2.1	up	Dehydrogenase/reductase SDR family member 7B
Ssa#TC87067	TC87067	2.1	up	Arginyl-tRNA synthetase, cytoplasmic
Ssa#S35553627	S35553627	2.1	up	gasterosteus aculeatus clone cfw236-h09 mrna sequence
Ssa#DW539342	DW539342	2.1	up	hypothetical protein LOC777623
Ssa#S35555864	S35555864	2.1	up	Transmembrane protein 179

Ssa#S32007249	DY735846	2.1	up	adipose differentiation-related protein
Omy#gi42627816	gi42627816	2.1	up	TPA_inf: Oncorhynchus mykiss mRNA for putative ISG12(1) protein (isg12(1) gene)
Ssa#DW550329	DW550329	2.1	up	equus caballus uncharacterized protein kiaa0892 mrna
Ssa#DY735824	DY735824	2.1	up	gasterosteus aculeatus clone cec35-e11 mrna sequence
Ssa#STIR26171	TC90333	2.1	up	deltex 3-like
Ssa#STIR25716	TC89658	2.1	up	xerodermacomplementation group a
Ssa#S30263395	S30263395	2.1	up	C3orf54
Ssa#S31976062	S31976062	2.1	up	ariadne homolog, ubiquitin-conjugating enzyme E2 binding protein, 1 like
Ssa#STIR12115	TC69576	2.0	up	Danio rerio si:ch211-107o23.1, mRNA (cDNA clone MGC:162374 IMAGE:7141973),
Ssa#DW178917	DW178917	2.0	up	Glycogen phosphorylase, muscle form
Omy#S15292679	S15292679	2.0	up	Cyclic AMP-dependent transcription factor ATF-5
Ssa#STIR23037	TC85639	2.0	up	arginine-mutated in early stage tumors
Ssa#S31963999	S31963999	2.0	up	H-2 class II histocompatibility antigen gamma chain
Omy#CA376742	CA376742	2.0	up	homo sapiens poly (adp-ribose) polymerase member 15 transcript variant mrna
Ssa#STIR09454	TC66037	2.0	up	n-myc (and stat) interactor
Ssa#S35682089	S35682089	2.0	up	Ubiquitin-like protein 1
Omy#S15312750	S15312750	2.0	up	UDP-glucuronosyltransferase
Ssa#DW470659	DW470659	2.0	up	danio rerio ags3
Ssa#S31983950	S31983950	2.0	up	fish virus induced TRIM protein
Ssa#S30239910	S30239910	2.0	up	NF-kappa-B 1 p105 subunit
Ssa#STIR04266	gi|209733467	2.0	up	ring finger protein 182
Ssa#S35548062	S35548062	2.0	up	Ubiquitin-like protein 1
Ssa#S35694019	S35694019	2.0	up	gallus gallus protein phosphatase regulatory subunit 3c mrna
Ssa#S30281775	S30281775	2.0	up	tetraodon nigroviridis full-length cdna
Ssa#STIR10783	TC67755	2.0	up	---NA---
Ssa#EG882135	EG882135	2.0	up	Poly polymerase 12
Ssa#DY734412	DY734412	2.0	up	osmerus mordax clone omor-rgc-505-015 ring finger protein 11
Omy#S18533898	S18533898	2.0	up	PDZ and LIM domain protein 4
Ssa#S30241113	S30241113	2.0	up	ADP-ribosylation factor-like protein 5B
Ssa#TC110559	TC110559	2.0	up	salmo salar clone bac chori214- complete sequence
Ssa#STIR19621	TC80485	2.0	up	serglycin precursor
Ssa#KSS1693	KSS1693	2.0	up	LOC100148704 protein
Ssa#S31971631	S31971631	2.0	up	danio rerio zgc:175142 (zgc:175142) mrna
Ssa#CL109Ctg1	CL109Contig1	2.0	up	H-2 class II histocompatibility antigen gamma chain
Ssa#S18857994	S18857994	2.0	up	matrix metalloproteinase
Ssa#CA046385	CA046385	2.0	up	phosphoinositide-3-kinase, regulatory subunit, polypeptide 3 (p55, gamma)
Ssa#DY714827	DY714827	2.0	up	: DEAD (Asp-Glu-Ala-Asp) box polypeptide 58
Ssa#STIR25176	TC88843	2.0	up	arginine-mutated in early stage tumors
Ssa#S35509463	EG795841	2.0	up	E3 ubiquitin-protein ligase CHFR
Ssa#DY735106	DY735106	2.0	up	caspase 10
Ssa#CB506335	CB506335	2.0	up	monodelphis domestica mgc84000 protein mrna
Ssa#CL365Ctg1	CL365Contig1	2.0	up	Transmembrane protein 66
Ssa#STIR22447	TC84745	2.0	up	translocase of outer mitochondrial membrane 5 homolog
Ssa#STIR10540	TC67430	2.0	up	Salmo salar physical map contig 483, genomic sequence
Ssa#DY706533	DY706533	2.0	up	similar to vertebrate type 1 tumor necrosis factor receptor shedding aminopeptidase regulator (ARTS-1)
Ssa#STIR12487	TC70092	2.0	up	transmembrane protein 30a
Ssa#KSS3416	KSS3416	2.0	up	6-phosphofructokinase type C
Ssa#STIR26172	TC90334	2.0	up	Salmo salar clone ssal-rgf-513-007 Nicotinamide phosphoribosyltransferase putative mRNA
Ssa#S35666023	S35666023	2.0	up	Serine/threonine-protein kinase PLK2
Ssa#STIR23443	TC86246	2.0	up	member ras oncogene family
Ssa#STIR25272	TC88985	2.0	up	poly (adp-ribose) polymerasemember 14
Omy#S34313882	S34313882	2.0	up	fra2 protein
Ssa#TC71753	TC71753	2.0	up	FAM114A2
Ssa#S32009517	S32009517	2.0	up	6-phosphofructokinase type C
Ssa#S35547567	S35547567	2.0	up	transport-associated protein
Ssa#STIR08509	TC64855	2.0	up	novel protein
Ssa#KSSb2728	KSSb2728	2.0	up	erlectin
Ssa#STIR10164	TC66931	2.0	up	serglycin precursor
Ssa#STIR23204	TC85885	2.0	up	protein tyrosine kinase src
Ssa#STIR11388	TC68587	2.0	up	Salmo salar clone HM4_1986 v-maf musculoaponeurotic fibrosarcoma oncogene-like (maffl) mRNA,
Ssa#STIR12058	TC69496	2.0	up	Salmo salar clone ssal-rgf-503-195 SAM and SH3 domain-containing protein 1 putative mRNA
Ssa#S35563017	S35563017	2.0	up	interferon-gamma receptor 1
Ssa#STIR12573	TC70220	2.0	up	galectin like protein
Ssa#STIR09117	TC65614	2.0	up	---NA---
Ssa#S35693907	S35693907	2.0	up	danio rerio ppar-alpha interacting complex protein 285 mrna
Ssa#STIR40927_S	TC112306	-2.0	down	novel protein similar to vertebrate monooxygenase, DBH-like 1 (MOXD1)
Ssa#STIR00078_3	DW580904	-2.0	down	interleukin 15
Ssa#S35491323	S35491323	-2.0	down	Pterin-4-alpha-carbinolamine dehydratase 2
Ssa#S35491193	S35491193	-2.0	down	fin bud initiation factor
Ssa#S30276496	S30276496	-2.0	down	Copine-3
Ssa#S35504873	S35504873	-2.0	down	C-factor
Ssa#STIR10787	TC67759	-2.0	down	Salmo salar clone HM5_2305 eukaryotic translation initiation factor 4E binding protein 2 (eif4ebp2) mRNA,
Ssa#DY740567	DY740567	-2.0	down	similar to Ras and Rab interactor 2
Ssa#STIR17136	TC76758	-2.0	down	---NA---
Ssa#KSS5021	KSS5021	-2.0	down	kinesin family member C1/zinc finger protein
Ssa#CL67Contig2	CL67Contig2	-2.0	down	Coronin-1A
Ssa#S18889731	S18889731	-2.0	down	salmo salar n-sulphoglucosamine sulphohydrolase mrna
Ssa#STIR08258	TC64551	-2.0	down	Plasmodium falciparum 3D7 chromosome 13
Ssa#S35704124	S35704124	-2.0	down	rad51 homolog C
Ssa#STIR15222	TC73973	-2.0	down	thimet oligopeptidase 1
Ssa#KSSb2232	KSSb2232	-2.0	down	main olfactory receptor-like protein
Ssa#STIR21971	TC84027	-2.0	down	cysteine-rich protein 1
Ssa#STIR11325	TC68511	-2.0	down	cytochromefamilysubfamilypolypeptide 1
Ssa#S30291070	DW578034	-2.0	down	Cell division protein kinase 2
Ssa#STIR38014	TC108084	-2.0	down	hypothetical protein NEMVEDRAFT_v1g143109
Ssa#S48439048_S	S48439048	-2.0	down	Acidic leucine-rich nuclear phosphoprotein 32 family member B
Ssa#S31965526	S31965526	-2.0	down	hypothetical protein LOC559398
Ssa#S35582906	S35582906	-2.0	down	Gastrotropin
Ssa#STIR18378	TC78603	-2.0	down	Oryza sativa (japonica cultivar-group) genomic DNA, chromosome 12
Ssa#CB516021	CB516021	-2.0	down	rattus norvegicus protein of bilaterial origin mrna
Omy#S34422869	S34422869	-2.0	down	hypothetical protein LOC567536
Ssa#STIR19655	TC80530	-2.0	down	CNS09SBSTetraodon nigroviridis BAC sequence
Ssa#S30244533	S30244533	-2.0	down	hypothetical protein LOC100002393
Ssa#STIR19300	TC79976	-2.0	down	---NA---
Ssa#S35501075	S35501075	-2.0	down	homeobox protein HoxA2aa
Ssa#STIR09126	TC65624	-2.0	down	---NA---

Ssa#S35585522	S35585522	-2.0	down	Regulator of G-protein signaling 18
Ssa#STIR21879	TC83885	-2.0	down	---NA---
Ssa#STIR23178	TC85843	-2.0	down	pdz binding kinase
Omy#S22914189	S22914189	-2.0	down	novel protein similar to vertebrate RAP1 interacting factor homolog (yeast) (RIF1)
Ssa#STIR13627	TC71700	-2.0	down	cox18 cytochrome c oxidase assembly homolog
Ssa#STIR05215	gi|209731551	-2.0	down	centrosomal protein of 27 kda
Ssa#STIR03123	gi|209735757	-2.0	down	centrosomal protein of 27 kda
Ssa#S48418472	S48418472	-2.0	down	canopy 4 precursor
Ssa#STIR15135	TC73841	-2.0	down	mu
Ssa#STIR08399	TC64732	-2.0	down	---NA---
Ssa#S30262999	S30262999	-2.0	down	similar to GTPase cRac1B
Ssa#S35501116	S35501116	-2.0	down	FACT complex subunit SSRP1
Ssa#KSS308	KSS308	-2.0	down	DNA polymerase subunit delta-2
Ssa#S31987373	S31987373	-2.0	down	Calponin-3
Ssa#S35605422	S35605422	-2.0	down	Kinetochore protein Spc25
Ssa#STIR42027	DY711324	-2.0	down	danio rerio im:7138629 (im:7138629) mrna
Ssa#S19096344	S19096344	-2.0	down	collagen a3(I)
Ssa#DW552356	DW552356	-2.0	down	integrin, alpha 6
Omy#CA351287	CA351287	-2.0	down	complement C4-1
Ssa#TC106546	TC106546	-2.0	down	danio rerio desmoplakin a mrna
Ssa#STIR13965	TC72184	-2.0	down	riken cdna d630039a03 gene
Ssa#STIR26184	TC90355	-2.0	down	solute carrier familymember 34
Ssa#S18891845	S18891845	-2.0	down	salmo salar clone ssal-rgf-509-117 beta- -galactosyltransferase 2 pseudogene cds
Ssa#STIR15413	TC74241	-2.0	down	---NA---
Ssa#DY737585_S	DY737585	-2.0	down	Replication factor C subunit 2
Ssa#S35528873	S35528873	-2.0	down	Eukaryotic translation initiation factor 4E type 2
Ssa#S35533557	EG819935	-2.1	down	Ubiquitin-conjugating enzyme E2 T
Ssa#STIR24278	TC87487	-2.1	down	chromosome 19 open reading frame 39
Omy#CU072135	CU072135	-2.1	down	enoyl Coenzyme A hydratase domain containing 1
Ssa#S30280749	S30280749	-2.1	down	similar to Potassium/sodium hyperpolarization-activated cyclic nucleotide-gated channel 4
Ssa#STIR22165	TC84316	-2.1	down	dehydrodolichyl diphosphate synthase
Ssa#STIR13443	TC71432	-2.1	down	---NA---
Ssa#S35514618_S	S35514618	-2.1	down	Deoxyuridine 5-triphosphate nucleotidohydrolase, mitochondrial precursor
Ssa#DW471278	DW471278	-2.1	down	similar to latent transforming growth factor beta binding protein 4,
Ssa#DW564686	DW564686	-2.1	down	Mitochondrial uncoupling protein 2
Ssa#STIR12840	TC70583	-2.1	down	---NA---
Ssa#STIR11067	TC68142	-2.1	down	peptidase m20 domain containing 1
Omy#BX859167	BX859167	-2.1	down	: wu:fa96e12
Omy#CX260733	CX260733	-2.1	down	novel protein similar to vertebrate neuroepithelial cell transforming gene 1 (NET1)
Omy#S19711047	CR367942	-2.1	down	cyclin B2
Ssa#EG847367	EG847367	-2.1	down	taeniopygia guttata adam metallopeptidase with thrombospondin type 1 8 preproprotein mrna
Ssa#S32005165	S32005165	-2.1	down	DNA topoisomerase 2-alpha
Ssa#S30239126	S30239126	-2.1	down	novel protein (zgc:154087)
Ssa#S35699279	S35699279	-2.1	down	Interactor protein for cytohesin exchange factors 1
Omy#S15278499	S15278499	-2.1	down	BTG3 protein
Ssa#STIR06431	gi|209155125	-2.1	down	copper metabolismdomain containing 1
Ssa#CK885403	CK885403	-2.1	down	glycosyltransferase 25 domain containing 2
Ssa#S31980390	S31980390	-2.1	down	DNA primase small subunit
Ssa#S30264478	S30264478	-2.1	down	LOC446284 protein
Ssa#STIR23402	TC86189	-2.1	down	Zebrafish DNA sequence from clone BUSM1-94E17 similar to vertebrate G protein-coupled receptor 12
Ssa#STIR20590	TC81907	-2.1	down	transgelin
Ssa#STIR08315	TC64630	-2.1	down	chromobox protein homolog 1
Ssa#STIR16188	TC75342	-2.1	down	homeobox protein
Ssa#S48399353	S48399353	-2.1	down	Si:dkeyp-35b8.5 protein
Ssa#S35497044	S35497044	-2.1	down	similar to K01C8.1
Ssa#STIR08188	TC64460	-2.1	down	pdz binding kinase
Ssa#STIR14240	TC72570	-2.1	down	mgc85526 protein
Ssa#DW583933	DW583933	-2.1	down	Interactor protein for cytohesin exchange factors 1
Ssa#S35675475	S35675475	-2.1	down	---NA---
Ssa#TC68821	TC68821	-2.1	down	Core histone macro-H2A.2
Ssa#STIR25227	TC88915	-2.1	down	---NA---
Ssa#STIR22433	TC84722	-2.1	down	inositol-trisphosphate 3-kinase c
Ssa#STIR25759	TC89711	-2.1	down	pigment epithelium-derived factor
Ssa#STIR13018	TC70831	-2.1	down	---NA---
Ssa#STIR21388	TC83155	-2.1	down	polymerase (dna-directed)delta 4
Ssa#DN047880	DN047880	-2.1	down	DNA polymerase delta catalytic subunit
Ssa#STIR10082	TC66821	-2.1	down	chromobox protein homolog 3
Ssa#S35547571	S35547571	-2.1	down	Krueppel-like factor 2
Ssa#STIR05090	gi|209731805	-2.1	down	transgelin
Ssa#CA058675	CA058675	-2.1	down	similar to potassium voltage-gated channel, shaker-related subfamily, beta member 2
Ssa#S35496324	S35496324	-2.1	down	Thrombospondin 4b
Ssa#STIR11752	TC69080	-2.1	down	rgm domainmember b
Ssa#STIR22312	TC84527	-2.1	down	ndt80 like dna-binding domain-containing protein
Ssa#DW578616	DW578616	-2.1	down	: Fanconi anemia, complementation group I
Ssa#S18842801	S18842801	-2.1	down	Securin
Omy#S15278173	S15278173	-2.1	down	Nitrilase homolog 1
Ssa#STIR22491	TC84818	-2.1	down	plasma retinol-binding protein 1
Ssa#S31964325_S	S31964325	-2.1	down	serine protease-like protein precursor
Ssa#S18892279	S18892279	-2.1	down	cytochrome P450 1A
Ssa#S35688015	S35688015	-2.1	down	Replication factor C subunit 2
Ssa#STIR16441	TC75714	-2.1	down	TSA: Hippoglossus hippoglossus all_halibut.147.C1 mRNA sequence
Ssa#S31984916	S31984916	-2.1	down	homo sapiens histone deacetylase 7 transcript variant mrna
Ssa#S35530555	S35530555	-2.1	down	similar to LOC515082 protein
Ssa#STIR26254	TC90457	-2.1	down	annexin a1
Omy#S34425219	S34425219	-2.1	down	ZN503_DANRERecName: Full=Zinc finger protein 503; AltName: Full=NocA-like zinc finger protein 2
Ssa#S18892282	S18892282	-2.1	down	salmo salar aryl hydrocarbon receptor 2 delta mrna
Ssa#STIR21577	TC83439	-2.1	down	---NA---
Ssa#STIR14323	TC72692	-2.1	down	acyl-synthetase family member 2
Ssa#STIR17778	TC77703	-2.1	down	Salmo salar clone ssal-evd-521-233 DAZ-associated protein 1 putative mRNA,
Ssa#STIR13201	TC71072	-2.1	down	gtp cyclohydrolase i feedback regulator
Ssa#STIR25997	TC90074	-2.1	down	Salmo salar clone ssal-rgf-503-337, novel cds
Ssa#S30286632	S30286632	-2.1	down	Sodium- and chloride-dependent GABA transporter 2
Ssa#CA043659	CA043659	-2.1	down	---NA---
Ssa#S48429157_S	S48429157	-2.1	down	CU051 protein
Ssa#S35507555	EG793933	-2.1	down	Calpain small subunit 1
Ssa#STIR08361	TC64687	-2.1	down	Zebrafish DNA sequence from clone CH73-46J18 in linkage group 10, complete sequence

Ssa#CL230Ctg1	CL230Contig1	-2.1	down	Vimentin
Omy#CR368123	CR368123	-2.1	down	hypothetical protein LOC557909
Ssa#S21512939	S21512939	-2.1	down	C type lectin receptor C
Ssa#S35489947	S35489947	-2.1	down	protein
Omy#S15290909	S15290909	-2.1	down	tropomodulin 4
Ssa#S18892477	S18892477	-2.1	down	aldolase B, fructose-bisphosphate
Omy#S34423313	S34423313	-2.1	down	methylenetetrahydrofolate dehydrogenase (NADP+ dependent) 1,
Ssa#STIR18143	TC78246	-2.1	down	glycerol-3-phosphate dehydrogenase 1-like
Ssa#STIR18340	TC78544	-2.1	down	G2/mitotic-specific cyclin-B1
Ssa#CA062362	CA062362	-2.1	down	Sister chromatid cohesion protein DCC1
Ssa#S35530808	EG817186	-2.1	down	Cystatin-B
Ssa#DY700052	DY700052	-2.1	down	danio rerio si:ch211- (si:ch211-) mrna
Ssa#EG862523	EG862523	-2.1	down	DNA polymerase subunit delta-2
Ssa#STIR03019	BT049051	-2.1	down	regulator of g-protein signaling 18
Ssa#STIR09350	TC65908	-2.1	down	eh domain-containing protein 4
Ssa#S18860876	S18860876	-2.1	down	H-2 class II histocompatibility antigen, A-Q alpha chain
Ssa#STIR15140	TC73846	-2.1	down	ectonucleotide pyrophosphatase phosphodiesterase 2
Ssa#KSS1763	KSS1763	-2.1	down	lymphocyte cytosolic plastin 1
Ssa#STIR15937	TC74981	-2.1	down	Zebrafish DNA sequence from clone CH211-155K24 in linkage group 4, complete sequence
Omy#S27588350	S27588350	-2.1	down	similar to matrix metalloproteinase 19
Ssa#STIR03201	gi|209735601	-2.1	down	fatty acid-bindingheart
Ssa#STIR23599	TC86488	-2.1	down	: Danio rerio wu:fd16g01 (wu:fd16g01), mRNA
Ssa#STIR04615	gi|209732761	-2.1	down	-dienoylreductase 2
Ssa#STIR20436	TC81696	-2.1	down	cd200 molecule
Ssa#DW543000	DW543000	-2.1	down	Enc1 protein
Omy#S22164185	S22164185	-2.1	down	Fibroleukin
Ssa#S35663677	S35663677	-2.1	down	Hypoxanthine-guanine phosphoribosyltransferase
Ssa#S32006933	S32006933	-2.1	down	phosphofructokinase, muscle b
Ssa#TC112747	TC112747	-2.1	down	: bridging integrator 1 isoform 1
Ssa#STIR12521	TC70140	-2.1	down	---NA---
Ssa#S31964102	S31964102	-2.1	down	T-cell immunoglobulin and mucin domain-containing protein 4 precursor
Omy#S15341130	S15341130	-2.1	down	oncorhynchus mykiss rtsox23 mrna
Ssa#S18888523	S18888523	-2.1	down	similar to ring finger protein 130
Ssa#S18867312	S18867312	-2.1	down	Ribonuclease UK114
Ssa#S30258747	S30258747	-2.1	down	Lymphocyte cytosolic protein 2
Ssa#STIR16248	TC75431	-2.1	down	dis3 mitotic control homolog
Ssa#STIR24501	TC87823	-2.2	down	Salmo salar physical map contig 483, genomic sequence
Ssa#S35700890	S35700890	-2.2	down	Inositol-trisphosphate 3-kinase A
Ssa#S48441015	S48441015	-2.2	down	contractile ring component anillin
Ssa#STIR15350	TC74161	-2.2	down	grb2-associated binding protein 3
Ssa#STIR21880	TC83886	-2.2	down	transient receptor potential cationsubfamilymember 1
Ssa#TC112827	TC112827	-2.2	down	RAD21 homolog
Ssa#STIR19215	TC79842	-2.2	down	thrombospondin 4
Ssa#S30284377	S30284377	-2.2	down	S100-B
Ssa#S35547210	EG833588	-2.2	down	Mediator of RNA polymerase II transcription subunit 22
Ssa#STIR09786	TC66455	-2.2	down	c-type lectin domain family 4 member e
Ssa#STIR05195	gi|209731591	-2.2	down	nuclear transport factor 2
Ssa#S35605165	S35605165	-2.2	down	C-reactive protein
Ssa#S35661746	EG896464	-2.2	down	Cell cycle progression protein 1
SsaHomCont3_080	SsaHomContl3	-2.2	down	beta-actin
Ssa#STIR11736	TC69061	-2.2	down	Takifugu rubripes DNA, highly conserved vertebrate non-coding sequence, element ID CNE335
Ssa#S37437536	S37437536	-2.2	down	fructose-1,6-bisphosphatase
Ssa#STIR17972	TC77993	-2.2	down	euchromatic histone-lysine n-methyltransferase 2
Ssa#STIR26143	TC90294	-2.2	down	---NA---
Ssa#STIR21414	TC83188	-2.2	down	atp-sensitive inward rectifier potassium channel 8
Ssa#STIR20456	TC81724	-2.2	down	t-box 15
Ssa#S30277425	S30277425	-2.2	down	danio rerio hypothetical loc100002747 mrna
Ssa#S35550599	S35550599	-2.2	down	novel protein similar to vertebrate syntaxin 12 (STX12)
Omy#TC133478	TC133478	-2.2	down	gasterosteus aculeatus clone cnb225-c04 mrna sequence
Omy#S34423610	S34423610	-2.2	down	Iroquois homeobox protein 1, a
Ssa#S35541554	S35541554	-2.2	down	Transmembrane protein 80
Omy#S15292139	S15292139	-2.2	down	phosphoribosyl transferase domain containing 1
Ssa#DY706488	DY706488	-2.2	down	LOC402831 protein
Ssa#STIR15079	TC73766	-2.2	down	largehomolog-associated protein 5
Ssa#S18849801	S18849801	-2.2	down	actinin alpha 3
Ssa#STIR24077	TC87210	-2.2	down	cytohesin 3
Ssa#S35516903	S35516903	-2.2	down	tetraodon nigroviridis full-length cdna
Ssa#S31964465	S31964465	-2.2	down	AF412833_1mismatch repair protein Msh2
Ssa#S35661538	S35661538	-2.2	down	hypothetical protein LOC379515
Ssa#S32001062	S32001062	-2.2	down	Phosphatidylinositol-binding clathrin assembly protein
Ssa#S18892418	S18892418	-2.2	down	glycerol-3-phosphate dehydrogenase
Ssa#S31971022	S31971022	-2.2	down	hypothetical protein LOC559560
Ssa#S35506545	S35506545	-2.2	down	interleukin-15 precursor
Ssa#STIR05716	gi|209730545	-2.2	down	fatty acid-bindingheart
Omy#TC165263	TC165263	-2.2	down	mus musculus radixin transcript variant mrna
Ssa#STIR23575	TC86457	-2.2	down	CNS0G45CTetraodon nigroviridis full-length cDNA
Ssa#S30295090	S30295090	-2.2	down	kinesin family member 20A
Ssa#STIR18270	TC78424	-2.2	down	---NA---
Ssa#STIR21920	TC83948	-2.2	down	---NA---
Omy#TC153278	TC153278	-2.2	down	similar to MLF1 interacting protein
Ssa#S48802141	S48802141	-2.2	down	homeobox protein HoxD10aa
Ssa#CX727458	CX727458	-2.2	down	similar to meningioma expressed antigen 5 (hyaluronidase)
Ssa#STIR02196	gi|209737623	-2.2	down	mannose-binding protein c precursor
Ssa#STIR16334	TC75555	-2.2	down	Salmo salar clone ssal-rgf-517-362 unknown large open reading frame mRNA, novel cds
Ssa#S47729178	S47729178	-2.2	down	Ependymin precursor
Omy#S22929208	S22929208	-2.2	down	solute carrier family 44, member 1
Ssa#STIR01236	gi|197632634	-2.2	down	parvalbumin
Ssa#S18890973	S18890973	-2.2	down	Glucosamine-6-phosphate isomerase
Omy#S34308973	S34308973	-2.2	down	similar to CG6428 CG6428-PA
Ssa#S35673660	S35673660	-2.2	down	4F2 cell-surface antigen heavy chain
Ssa#S31978957	S31978957	-2.2	down	danio rerio amyloid beta precursor protein- family member 1 interacting protein mrna
Omy#S15288506	S15288506	-2.2	down	Collagen alpha-1I chain precursor
Ssa#STIR19380	TC80097	-2.2	down	---NA---
Ssa#S18889843	S18889843	-2.2	down	ceroid-lipofuscinosis, neuronal 6, late infantile, variant
Ssa#S30244934	S30244934	-2.2	down	integrin, beta 5
Ssa#STIR17204	TC76871	-2.2	down	typealpha 1

Ssa#S48429157	S48429157	-2.2	down	CU051 protein
Ssa#S35678863	S35678863	-2.2	down	Complement factor D precursor
Ssa#STIR23563	TC86442	-2.2	down	---NA---
Ssa#S35663749	S35663749	-2.2	down	Si:ch211-200o3.2 protein
Omy#S34426159	S34426159	-2.2	down	Transient receptor potential cation channel subfamily V member 1
Ssa#TC102141	TC102141	-2.2	down	LOC402831 protein
Ssa#STIR09659	TC66303	-2.2	down	regulator of g-protein signaling 1
Ssa#STIR22640	TC85035	-2.2	down	Lithognathus mormyrus clone lmos8p04c12 mRNA sequence
Omy#S34422216	S34422216	-2.2	down	NUF2, NDC80 kinetochore complex component
Ssa#S35660972	S35660972	-2.2	down	Complement factor D precursor
Omy#S34308813	S34308813	-2.2	down	: si:dkey-231a18.1
Ssa#STIR07999	TC64239	-2.2	down	---NA---
Omy#S15320906	S15320906	-2.2	down	Homeobox protein Dlx5a
Ssa#CB518090	CB518090	-2.2	down	homo sapiens leprecan-like 1 transcript variant mrna
Ssa#S18845671	S18845671	-2.2	down	C6orf125
Ssa#STIR18549	TC78862	-2.2	down	myocilin
Ssa#STIR20204	TC81357	-2.2	down	subfamilymember 6
Ssa#S35552426	S35552426	-2.2	down	C-X-C motif chemokine 14
Ssa#S35538406	S35538406	-2.2	down	Vimentin
Ssa#S35579591	S35579591	-2.2	down	bone morphogenetic protein 8
Ssa#S32010860	S32010860	-2.2	down	hypothetical protein LOC569254
Ssa#S30278631	DW565599	-2.2	down	: collagen, type VI, alpha 2
Ssa#S18892468	S18892468	-2.2	down	troponin I
Ssa#S35559271	S35559271	-2.2	down	Glutathione S-transferase P
Ssa#S48414611	S48414611	-2.2	down	non-SMC condensin I complex, subunit H
Ssa#CL381Ctg1	CL381Contig1	-2.2	down	: Rac GTPase activating protein 1
Ssa#STIR29588_S	TC95506	-2.2	down	Tissue factor pathway inhibitor 2 precursor
Ssa#EG848604	EG848604	-2.2	down	Peptidylprolyl isomerase-like 5
Ssa#STIR04348	gi|209733303	-2.2	down	phosphoserine phosphatase
Con_CANDS_13	gi|209155391|gb|BT045	-2.2	down	391|gb|BT045666.1| Salmo salar clone ssal-rgf-528-084 Cytochrome P450 1A1 putative mRNA,
Ssa#S35552627	S35552627	-2.2	down	Glycophorin-C
Ssa#STIR16319	TC75538	-2.2	down	alcoholiron1
Ssa#STIR10659	TC67591	-2.2	down	interleukin 15
Ssa#S18849636	S18849636	-2.2	down	complement C4-1
Ssa#STIR37133	TC106804	-2.2	down	Profilin-1
Ssa#KSS3203	KSS3203	-2.2	down	Pigment epithelium-derived factor
Ssa#STIR03265	gi|209735473	-2.2	down	chromosome 12 open reading frame 32
Ssa#STIR21893	TC83911	-2.2	down	creatine kinase b-type
Ssa#STIR14857	TC73467	-2.2	down	Salmo salar clone 31E09 TCR-alpha/delta locus, genomic sequence
Omy#S15319979	S15319979	-2.2	down	similar to epimorphin
Ssa#TC86360	TC86360	-2.2	down	NUF2, NDC80 kinetochore complex component
Ssa#STIR21471	TC83275	-2.2	down	Salmo salar neurogranin, TIP41-like protein (TIP41), MHC class II antigen beta chain (Sasa-DBB)
Ssa#DW555778	DW555778	-2.2	down	Acid phosphatase-like protein 2
Ssa#S30247095	S30247095	-2.2	down	similar to adenosine deaminase
Ssa#S35661887	S35661887	-2.2	down	SH3 domain-binding glutamic acid-rich-like protein
Ssa#TC82076	TC82076	-2.3	down	DNA replication licensing factor mcm2
Ssa#STIR36498	TC105855	-2.3	down	tetraodon nigroviridis full-length cdna
Omy#TC146371	TC146371	-2.3	down	1-acyl-sn-glycerol-3-phosphate acyltransferase theta-B
Ssa#STIR15393	TC74219	-2.3	down	Salmo salar clone ssal-rgf-511-028 Repressor of RNA polymerase III transcription MAF1 homolog
Ssa#STIR18615	TC78966	-2.3	down	similar to vertebrate phosphatidylserine synthase 1 (PTDSS1),
Ssa#S18871989	S18871989	-2.3	down	UDP-GlcNAc:betaGal beta-1,3-N-acetylglucosaminyltransferase 5
Ssa#S30289193	S30289193	-2.3	down	Glycogen phosphorylase, muscle form
Ssa#STIR40482	TC111676	-2.3	down	tetraodon nigroviridis full-length cdna
Ssa#STIR08912	TC65341	-2.3	down	thbs3a protein
Ssa#S30292184	S30292184	-2.3	down	novel protein similar to vertebrate integrin, beta-like 1 (with EGF-like repeat domains) (ITGBL1, zgc:112304)
Omy#TC143379	TC143379	-2.3	down	Tenomodulin
Omy#CX252908	CX252908	-2.3	down	: si:dkey-217m5.5
Ssa#STIR24955	TC88511	-2.3	down	Salmo salar clone BAC CHORI214-424M17, complete sequence
Ssa#S35507967	S35507967	-2.3	down	aspartic acid-rich protein aspolin2
Ssa#S35503902	S35503902	-2.3	down	ATP synthase subunit e, mitochondrial
Ssa#S35486376	S35486376	-2.3	down	Glucosamine-6-phosphate isomerase
Ssa#STIR22391	TC84649	-2.3	down	bcl2-like 1
Ssa#S30240850	S30240850	-2.3	down	Transcription elongation factor 1 homolog
Ssa#CA051429	CA051429	-2.3	down	similar to Kinesin-like protein KIF9
Ssa#STIR22886	TC85401	-2.3	down	-dienoylreductase 2
Ssa#S35690206	S35690206	-2.3	down	Histone H2A.Z
Ssa#TC92565	TC92565	-2.3	down	proprotein convertase subtilisin/kexin type 5b
Ssa#STIR04672	gi|209732647	-2.3	down	allograft inflammatory factor 1
Ssa#STIR19442	TC80208	-2.3	down	fbp32 precursor
Ssa#S35559013	S35559013	-2.3	down	Inner centromere protein B
Ssa#STIR08721	TC65103	-2.3	down	glutamate-rich 1
Ssa#STIR25685	TC89609	-2.3	down	fa65b_danreame: full=protein fam65b
Ssa#STIR00161_2	gi|209155391	-2.3	down	cytochrome p450 1a
Ssa#STIR25177	TC88844	-2.3	down	---NA---
Ssa#S31993629	S31993629	-2.3	down	rattus norvegicus nei like 3 (coli) mrna
Ssa#STIR26104	TC90240	-2.3	down	---NA---
Ssa#S30270134	S30270134	-2.3	down	danio rerio si:dkey- mrna
Ssa#S35664580	S35664580	-2.3	down	hypothetical protein LOC792608
Omy#S34425130	S34425130	-2.3	down	G-2 and S-phase expressed 1
Ssa#TC99740	TC99740	-2.3	down	DNA replication licensing factor mcm2
Ssa#S31974794	S31974794	-2.3	down	Lipid phosphate phosphohydrolase 2
Ssa#STIR21472	TC83276	-2.3	down	6-phosphofructo-2-kinase fructose--biphosphatase 2
Omy#S34423612	S34423612	-2.3	down	danio rerio zgc:64166 (zgc:64166) mrna
Ssa#S35590781	S35590781	-2.3	down	Epithelial membrane protein 3
Ssa#STIR19027	TC79570	-2.3	down	solute carrier familymember 34
Ssa#CX354070	CX354070	-2.3	down	ATPase, Na+/K+ transporting, beta 2b polypeptide
Ssa#CA062184	CA062184	-2.3	down	danio rerio zgc: mrna (cdna clone mgc:173632 image:5409713)
Ssa#TC98512	TC98512	-2.3	down	homo sapiens leucine rich repeat containing 17 transcript variant mrna
Ssa#S35557680	S35557680	-2.3	down	zebrafish dna sequence from clone ch211-197n1 in linkage group 1
Ssa#S35521470	S35521470	-2.3	down	Metallothionein
Ssa#STIR02608	gi|209736789	-2.3	down	mannose-binding protein c precursor
Ssa#STIR08784	TC65183	-2.3	down	Oncorhynchus mykiss carbonic anhydrase 1 (LOC100135826), mRNA
Ssa#STIR02103	gi|209737811	-2.3	down	riken cdna 2610036l11 gene
Ssa#S35698682	S35698682	-2.3	down	Fibronectin
Omy#CA363999	CA363999	-2.3	down	zebrafish dna sequence from clone ch1073-98k8 in linkage group complete sequence
Ssa#S35601168	S35601168	-2.3	down	Fumarylacetoacetase

Ssa#S35706248	S35706248	-2.3	down	: wu:fa96e12
Ssa#S31978033	S31978033	-2.3	down	danio rerio hypothetical loc572418 mrna
Ssa#STIR18682	TC79055	-2.3	down	CNS0FZZMTetraodon nigroviridis full-length cDNA
Ssa#S18866519	S18866519	-2.3	down	BTB (POZ) domain containing 6
Ssa#TC95364	TC95364	-2.3	down	xenopus tropicalis smad family member 9 mrna
Ssa#S30241518	S30241518	-2.3	down	Lipocalin precursor
Omy#S34308651	S34308651	-2.3	down	polo-like kinase 4
Ssa#EG875527	EG875527	-2.3	down	periplakin
Ssa#STIR04359	gi|209733281	-2.3	down	fatty acid-bindingheart
Omy#S15332379	S15332379	-2.3	down	minichromosome maintenance complex component 7
Ssa#S35564035	S35564035	-2.3	down	Glutathione peroxidase 7 precursor
Ssa#STIR26154	TC90307	-2.3	down	codanin 1
Ssa#STIR08140	TC64408	-2.3	down	zinc finger protein 665
Ssa#STIR21278	TC82979	-2.3	down	bub1 budding uninhibited by benzimidazoles 1 homolog beta
Ssa#S32009407	S32009407	-2.3	down	zic family member 3 heterotaxy 1 (odd-paired homolog, Drosophila)
Ssa#STIR18924	TC79416	-2.3	down	Zebrafish DNA sequence from clone CH211-127M24 in linkage group 20, complete sequence
Ssa#S32005026	S32005026	-2.3	down	Serum paraoxonase/arylesterase 2
Ssa#DY728112	DY728112	-2.3	down	similar to olfactomedin-like 2A
Omy#S34422225	S34422225	-2.3	down	similar to Pde7a protein
Ssa#S30289341	S30289341	-2.3	down	Clusterin precursor
Ssa#CK876940	CK876940	-2.3	down	hypothetical protein LOC723999
Ssa#S35676032	S35676032	-2.3	down	homeobox protein HoxC9aa
Omy#CA376021	CA376021	-2.3	down	Transforming growth factor-beta-induced protein ig-h3
Omy#S15286687	S15286687	-2.3	down	cyclin E
Ssa#STIR22785	TC85255	-2.3	down	interleukin 15
Ssa#S18848091	S18848091	-2.3	down	Complement factor D precursor
Ssa#S35578870	S35578870	-2.3	down	danio rerio manic fringe mrna (cdna clone mgc:191355 image:100059664)
Ssa#EG855840	EG855840	-2.3	down	similar to MAM domain containing 2
Ssa#CL171Ctg1	CL171Contig1	-2.3	down	Proliferating cell nuclear antigen
Ssa#STIR16220	TC75394	-2.3	down	---NA---
Ssa#S18892278	S18892278	-2.3	down	cytochrome P450 1A
Ssa#STIR25761	TC89715	-2.3	down	n-terminal ef-hand calcium binding protein 1
Ssa#STIR24329	TC87560	-2.3	down	---NA---
Ssa#S35664069	S35664069	-2.3	down	EF-hand domain (C-terminal) containing 1
Omy#S34315216	S34315216	-2.3	down	eptatretus burgeri variable lymphocyte receptor b
Ssa#STIR06658	gi|209154663	-2.3	down	src-likeisoform cra_a
Ssa#CX353371	CX353371	-2.3	down	danio rerio zgc:66125 (zgc:66125) mrna
Ssa#DW542634	DW542634	-2.3	down	Neuropilin 1a
Ssa#S30284612	S30284612	-2.3	down	novel protein (zgc:153922)
Ssa#KSS1424	KSS1424	-2.3	down	Histone H2A.Z
Ssa#S18849203	S18849203	-2.3	down	small heat shock protein
Ssa#STIR43400_S	CA060333	-2.3	down	Hypoxanthine-guanine phosphoribosyltransferase
Ssa#S35530866	S35530866	-2.3	down	Cyclin-dependent kinases regulatory subunit 1
Omy#CU065468	CU065468	-2.3	down	Werner helicase interacting protein
Ssa#STIR22793	TC85267	-2.3	down	---NA---
Ssa#S30241991	S30241991	-2.4	down	Mitotic spindle assembly checkpoint protein MAD2A
Omy#CX254853	CX254853	-2.4	down	Histone-binding protein RBBP4
Ssa#TC83409	TC83409	-2.4	down	zebrafish dna sequence from clone dkeyp-85e10 in linkage group 19 sequence
Ssa#STIR18326	TC78523	-2.4	down	Salmo salar clone ssal-rgf-523-325 Flotillin-1 putative mRNA,
Ssa#S30274793	S30274793	-2.4	down	Platelet-derived growth factor receptor-like
Ssa#STIR08108	TC64371	-2.4	down	lysyl oxidase
Ssa#STIR13244	TC71135	-2.4	down	---NA---
Ssa#KSSb2771	KSSb2771	-2.4	down	Nuclear ubiquitous casein and cyclin-dependent kinases substrate
Ssa#STIR09173	TC65682	-2.4	down	Salmo salar clone ssal-rgf-522-035 EBV-induced G-protein coupled receptor 2 putative mRNA,
Omy#S19710761	S19710761	-2.4	down	hypothetical protein BRAFLDRAFT_104497
Ssa#STIR04905	gi|209732177	-2.4	down	heme-binding protein 2
Omy#CA366758	CA366758	-2.4	down	Acid phosphatase-like protein 2
Ssa#CL479Ctg1	CL479Contig1	-2.4	down	tetraodon nigroviridis full-length cdna
Ssa#STIR15827	TC74841	-2.4	down	Salmo salar clone ssal-rgf-527-019 Mitochondrial folate transporter/carrier putative mRNA,
Ssa#STIR26180	TC90346	-2.4	down	Salmo salar clone ssal-rgf-541-268 unknown large open reading frame mRNA, novel cds
Ssa#S31997908	S31997908	-2.4	down	Methylmalonate-semialdehyde dehydrogenase
Ssa#S35604333	S35604333	-2.4	down	eukaryotic translation initiation factor 4E binding protein 3-2
Ssa#DW549449	DW549449	-2.4	down	novel protein similar to vertebrate ATP-binding cassette sub-family A ABC1 member 2 (ABCA2)
Ssa#STIR25549	TC89398	-2.4	down	Lymphokine-activated killer T-cell-originated protein kinase homolog
Ssa#S35498243	S35498243	-2.4	down	: si:dkey-221h15.4
Omy#BX860134	BX860134	-2.4	down	similar to acyl-CoA synthetase family member 3
Ssa#S35492134	S35492134	-2.4	down	Membrane-spanning 4-domains subfamily A member 12
Ssa#S35495861	S35495861	-2.4	down	danio rerio mox-2 protein mrna
Ssa#STIR19943	TC80973	-2.4	down	---NA---
Ssa#STIR10006	TC66729	-2.4	down	---NA---
Omy#S34424145	S34424145	-2.4	down	2,4-dienoyl CoA reductase 2
Ssa#STIR21127	TC82745	-2.4	down	Mouse DNA sequence from clone RP23-321B23 on chromosome 2 Contains the 3' end of the Bcas1 gene
Ssa#EG896300	EG896300	-2.4	down	: sc:d0347
Ssa#S35488278	S35488278	-2.4	down	hypothetical protein LOC100158493
Ssa#STIR22281	TC84483	-2.4	down	---NA---
Ssa#STIR09533	TC66141	-2.4	down	---NA---
Ssa#STIR10121	TC66873	-2.4	down	---NA---
Ssa#STIR13967	TC72187	-2.4	down	---NA---
Ssa#STIR15526	TC74395	-2.4	down	family with sequence similaritymember a
Ssa#S35695545	S35695545	-2.4	down	Regulator of G-protein signaling 18
Ssa#DY693967	DY693967	-2.4	down	serum deprivation response protein
Ssa#DY740456	DY740456	-2.4	down	Probable E3 ubiquitin-protein ligase HECTD2
Ssa#DW541190	DW541190	-2.4	down	ZWILC_DANRERecName: Full=Protein zwilch homolog
Ssa#STIR06506	gi|209154971	-2.4	down	ebv-induced g-protein coupled receptor 2
Ssa#S18861347	S18861347	-2.4	down	thiosulfate sulfurtransferase KAT
Ssa#S35552642	S35552642	-2.4	down	Si:dkey-8l13.4 protein
Omy#TC161061	TC161061	-2.4	down	Protein-lysine 6-oxidase
Ssa#STIR21842	TC83828	-2.4	down	complement component 6
Ssa#S31974010	S31974010	-2.4	down	mus musculus high mobility group at-hook 1 transcript variant mrna
Ssa#STIR22397	TC84660	-2.4	down	Zebrafish DNA sequence from clone DKEY-31M21 in linkage
Ssa#S18848194	S18848194	-2.4	down	Complement factor D precursor
Ssa#STIR13253	TC71146	-2.4	down	protein kinase c and casein kinase substrate in neurons 1
Ssa#S48405112_S	S48405112	-2.4	down	hypothetical LOC558477
Ssa#DW571754	DW571754	-2.4	down	tetraodon nigroviridis full-length cdna
Ssa#STIR22285	TC84489	-2.4	down	Zebrafish DNA sequence from clone CH211-168O15 in linkage group 7, complete sequence
Ssa#S35534009	S35534009	-2.4	down	Membrane-spanning 4-domains subfamily A member 15

Omy#TC165689	TC165689	-2.4	down	danio rerio type alpha 2-like mrna
Ssa#KSS3351	KSS3351	-2.4	down	DNA replication licensing factor mcm2
Ssa#S35539178	S35539178	-2.4	down	Otoraplin
Ssa#S35561882	S35561882	-2.4	down	homo sapiens anoctamin 6 transcript variant mrna
Ssa#S35697623	S35697623	-2.4	down	similar to Helicase-like transcription factor (TNF-response element-binding protein) (P113)
Ssa#STIR14171	TC72474	-2.4	down	---NA---
Ssa#STIR20462	TC81732	-2.4	down	galectin like protein
Ssa#S47729029	S47729029	-2.4	down	Cytochrome c oxidase subunit 4 isoform 2, mitochondrial precursor
Omy#S34424554	S34424554	-2.4	down	Squamous cell carcinoma antigen recognised by T cells 3
Ssa#S32007313	S32007313	-2.4	down	Pigment epithelium-derived factor
Omy#S34309870	S34309870	-2.4	down	nuclear oncoprotein skia
Ssa#STIR12319	TC69860	-2.4	down	annexin a2
Ssa#STIR12252	TC69769	-2.4	down	rho family gtpase 3
Omy#CA379879	CA379879	-2.4	down	TPA: leukemia inhibitory factor receptor.a
Omy#S23941956	S23941956	-2.4	down	similar to sarcalumenin
Ssa#DY716769	DY716769	-2.4	down	: wu:fi38h09
Ssa#KSSb2334	KSSb2334	-2.4	down	SJCHGC09227 protein
Ssa#S35709147	S35709147	-2.4	down	parvalbumin beta
Ssa#TC106540	TC106540	-2.4	down	TNF receptor-associated factor 6
Ssa#S18891260	CB515159	-2.4	down	type I collagen alpha 2 chain
Ssa#STIR12970	TC70769	-2.5	down	collagen triple helix repeat containing 1
Ssa#STIR24855	TC88359	-2.5	down	---NA---
Ssa#DW181724	DW181724	-2.5	down	oncorhynchus mykiss na k atpase alpha subunit isoform 2 mrna
Ssa#CK878774	CK878774	-2.5	down	switch-associated protein 70
Ssa#S31974193	S31974193	-2.5	down	Cyclin-A2
Ssa#S30257488	S30257488	-2.5	down	Vat1 protein
Ssa#STIR13771	TC71912	-2.5	down	heterogeneous nuclear ribonucleoprotein l
Ssa#STIR26261	TC90467	-2.5	down	---NA---
Omy#CB490524	CB490524	-2.5	down	DNA
Ssa#STIR21226	TC82889	-2.5	down	Gallus gallus finished cDNA, clone ChEST786g11
Ssa#S32005649	S32005649	-2.5	down	DNA primase large subunit
Omy#TC144408	TC144408	-2.5	down	CN130 protein
Ssa#CL477Ctg1	CL477Contig1	-2.5	down	tetraodon nigroviridis full-length cdna
Ssa#STIR02464	gi|209737083	-2.5	down	mannose-binding protein c precursor
Ssa#CX357979	CX357979	-2.5	down	fish virus induced TRIM protein
Ssa#S18886064	S18886064	-2.5	down	: putative nuclear receptor subfamily 2 group F member 2
Omy#CU073092	CU073092	-2.5	down	Tenascin precursor
Ssa#STIR19197	TC79815	-2.5	down	---NA---
Ssa#STIR20074	TC81161	-2.5	down	typealpha 1
Ssa#STIR34677	TC103201	-2.5	down	collectin sub-family member 12
Ssa#STIR10872	TC67876	-2.5	down	dek oncogene (dna binding)
Ssa#STIR13335	TC71265	-2.5	down	nuclear transport factor 2
Ssa#STIR19840	TC80808	-2.5	down	Zebrafish DNA sequence from clone DKEY-29J9 in linkage group 21, complete sequence
Ssa#TC78681	TC78681	-2.5	down	similar to TBC1 domain family member 12
Ssa#S19100003	S19100003	-2.5	down	mus musculus autophagy-related 4c transcript variant mrna
Ssa#S35604398	S35604398	-2.5	down	similar to NY-REN-58 antigen
Omy#S34308906	S34308906	-2.5	down	Wu:fd18f09 protein
Ssa#S35580189	EG866567	-2.5	down	im:7038599
Ssa#S35661535	S35661535	-2.5	down	XTP3-transactivated gene A protein homolog
Ssa#S26643985	DQ163908	-2.5	down	growth hormone receptor isoform 2 precursor
Ssa#STIR22824	TC85311	-2.5	down	---NA---
Omy#S34315092	S34315092	-2.5	down	NG,NG-dimethylarginine dimethylaminohydrolase 2
Ssa#S18841766	S18841766	-2.5	down	Ictacalcin
Ssa#STIR14324	TC72693	-2.5	down	spindle pole body component 24 homolog
Ssa#DW575444	DW575444	-2.5	down	Arachidonate 5-lipoxygenase
Ssa#TC106671	TC106671	-2.5	down	Krueppel-like factor 6
Ssa#S30294406	S30294406	-2.5	down	Replication factor C subunit 2
Omy#S15339533	S15339533	-2.5	down	RUN and FYVE domain containing 2
Ssa#STIR10531	TC67418	-2.5	down	: Danio rerio im:7151384 (im:7151384), mRNA
Ssa#KSS2706	KSS2706	-2.5	down	---NA---
Ssa#S32008512	S32008512	-2.5	down	Transcription cofactor HES-6
Ssa#STIR00161_4	gi|209155391	-2.5	down	cytochrome p450 1a
Ssa#S35552719	S35552719	-2.5	down	LGN-like
Ssa#STIR21729	TC83656	-2.5	down	short chain dehydrogenase reductase familymember 5
Ssa#TC92882	TC92882	-2.5	down	Si:dkey-202b22.2
Ssa#DW537368	DW537368	-2.5	down	: novel protein similar to vertebrate phosphatidic acid phosphatase type 2B (PPAP2B)
Ssa#STIR02966	gi|209736071	-2.5	down	glutathione s-transferase pi
Ssa#EG904636	EG904636	-2.5	down	danio rerio leucine-rich repeats and wd repeat domain containing 1 mrna
Ssa#STIR06626	gi|209154727	-2.5	down	coiled-coil domain containing 104
Omy#CA357316	CA357316	-2.5	down	Sushi repeat-containing protein SRPX2 precursor
Ssa#STIR16038	TC75123	-2.5	down	Oncorhynchus mykiss carbonic anhydrase 1 (LOC100135826), mRNA
Omy#S34422375	S34422375	-2.5	down	latimeria clone vmrc4- complete sequence
Ssa#STIR17882	TC77860	-2.5	down	---NA---
Ssa#STIR22661	TC85069	-2.5	down	nuclear ubiquitous casein and cyclin-dependent kinases substrate
Ssa#S35679641	EG914359	-2.5	down	Lipid phosphate phosphohydrolase 1
Ssa#NP1610644	NP1610644	-2.5	down	beta-1,3-galactosyltransferase
Ssa#STIR17712	TC77605	-2.5	down	protein o-mannosyltransferase
Ssa#DW570850	DW570850	-2.5	down	Fermitin family homolog 3
Ssa#STIR15074	TC73760	-2.5	down	Salmo salar clone ssal-rgf-524-163 Rho-related GTP-binding protein RhoE precursor putative mRNA,
Ssa#KSS840	KSS840	-2.5	down	Galactonate dehydratase
Omy#S18158224	S18158224	-2.5	down	troponin I, skeletal, fast 2b.1 isoform 1
Ssa#STIR17509	TC77319	-2.5	down	truncated hyaluronidase
Ssa#S31963313	S31963313	-2.6	down	tetraodon nigroviridis full-length cdna
Omy#CA383936	CA383936	-2.6	down	similar to periostin
Ssa#STIR22405	TC84675	-2.6	down	lipase a
Ssa#EG882331	EG882331	-2.6	down	novel protein
Ssa#STIR08927	TC65361	-2.6	down	---NA---
Ssa#S45783609	S45783609	-2.6	down	homeobox protein HoxA13aa
Ssa#STIR08969	TC65414	-2.6	down	cxc chemokine d1
Omy#S34424773	S34424773	-2.6	down	similar to MGC115669 protein
Ssa#STIR11460	TC68690	-2.6	down	smt3 suppressor of mif two 3 homolog 2
Ssa#STIR25397	TC89168	-2.6	down	Zebrafish DNA sequence from clone DKEY-54K13 in linkage group 3, complete sequence
Ssa#S31995510	S31995510	-2.6	down	tsa: hippoglossus hippoglossus mrna sequence
Ssa#S30271957	S30271957	-2.6	down	Peroxisomal trans-2-enoyl-CoA reductase
Ssa#STIR03711	gi|209734577	-2.6	down	tescalcin
Omy#S15315701	S15315701	-2.6	down	hypothetical protein LOC559398

Ssa#S35605514	S35605514	-2.6	down	zinc finger protein 423-like
Ssa#S35590794	S35590794	-2.6	down	Transcription factor HES-1
Ssa#S35602677	S35602677	-2.6	down	S-formylglutathione hydrolase
Ssa#STIR15056	TC73738	-2.6	down	---NA---
Ssa#TC109012	TC109012	-2.6	down	cyclin E
Omy#BX081049	BX081049	-2.6	down	gasterosteus aculeatus clone cgx58-c04 mrna sequence
Ssa#EG879084	EG879084	-2.6	down	Histone H1-beta, late embryonic
Ssa#S30270686	S30270686	-2.6	down	Zinc finger protein ZIC 2
Ssa#S30285553	DW572521	-2.6	down	Lipid phosphate phosphohydrolase 2
Ssa#STIR21603	TC83478	-2.6	down	mannose-binding protein c precursor
Ssa#STIR23587	TC86471	-2.6	down	solute carrier family 12 (potassium chloride transporters)member 9
Ssa#STIR17309	TC77016	-2.6	down	dek oncogene (dna binding)
Ssa#TC72531	TC72531	-2.6	down	Butyrate response factor 1
Ssa#S30295611	S30295611	-2.6	down	similar to H.sapiens EFEMP1, EGF-containing fibulin-like extracellular matrix protein 1 (EFEMP1)
Ssa#S31963747	S31963747	-2.6	down	Cytosolic sulfotransferase 2
Ssa#STIR25924	TC89977	-2.6	down	structural maintenance of chromosomes 2
Ssa#STIR15063	TC73748	-2.6	down	Takifugu rubripes HoxAa gene cluster, complete sequence
Ssa#S35506441	S35506441	-2.6	down	Nicotinamide riboside kinase 2
Ssa#STIR20109	TC81209	-2.6	down	Zebrafish DNA sequence from clone CH211-208I16 in linkage group 18, complete sequence
Ssa#STIR12150	TC69624	-2.6	down	---NA---
Ssa#S32011142	S32011142	-2.6	down	homo sapiens solute carrier organic anion transporter member 2b1 transcript variant mrna
Ssa#S18849636_S	BT071912.1	-2.6	down	complement C4-1
Ssa#STIR11725	TC69045	-2.6	down	replication protein32kda
Ssa#S30246228	S30246228	-2.6	down	carbamoyl-phosphate synthetase 2, aspartate transcarbamylase, and dihydroorotase
Omy#S19712179	S19712179	-2.6	down	DNA polymerase subunit delta-2
Ssa#STIR12069	TC69509	-2.6	down	---NA---
Ssa#STIR11481	TC68720	-2.6	down	chromosome 10 open reading frame 33
Omy#S34310890	S34310890	-2.6	down	strongylocentrotus purpuratus dna topoisomeraseii_beta mrna
Ssa#STIR20779	TC82201	-2.6	down	---NA---
Ssa#STIR17006	TC76573	-2.6	down	growth arrest-specific 1
Omy#CU068949	CU068949	-2.6	down	strongylocentrotus purpuratus hypothetical loc577602 mrna
Ssa#STIR21939	TC83973	-2.6	down	Zebrafish DNA sequence from clone DKEY-38P8 in linkage group 7, complete sequence
Ssa#STIR24256	TC87456	-2.6	down	Nautilia profundicola AmH, complete genome
Ssa#S35482990	S35482990	-2.6	down	hucho taimen clone za-35 microsatellite sequence
Ssa#CL47Contig1	CL47Contig1	-2.6	down	Regulator of G-protein signaling 1
Ssa#STIR35406_S	TC104251	-2.6	down	CXC chemokine d1
Ssa#S30281918	S30281918	-2.6	down	Zinc finger protein ZIC 2
Ssa#STIR18459	TC78725	-2.6	down	protein
Ssa#S35594270	S35594270	-2.6	down	chordin-like
Omy#S15290943	S15290943	-2.6	down	zebrafish dna sequence from clone ch211-154p8 in linkage group complete sequence
Ssa#S18887488	S18887488	-2.6	down	danio rerio galectin-3-binding mrna
Ssa#S22713494	S22713494	-2.6	down	type I keratin S8
Ssa#S35555901	S35555901	-2.6	down	transforming, acidic coiled-coil containing protein 3
Ssa#STIR16864	TC76361	-2.6	down	hemicentin 1
Ssa#STIR08149	TC64420	-2.6	down	c-c motif chemokine 25 precursor
Omy#S34310188	S34310188	-2.6	down	homogentisate 1, 2-dioxygenase, isoform CRA_b
Ssa#STIR08822	TC65229	-2.6	down	interleukin 15
Ssa#S35585495	S35585495	-2.6	down	Filamin-binding LIM protein 1
Ssa#S35678957	S35678957	-2.6	down	: butyrophilin, subfamily 2, member A2
Ssa#STIR23656	TC86583	-2.6	down	Rhesus Macaque BAC CH250-166C17 () complete sequence
Ssa#STIR12975	TC70774	-2.6	down	Gallus gallus finished cDNA, clone ChEST474g11
Omy#S18095701	S18095701	-2.7	down	Col2a1a protein
Ssa#S18870968	S18870968	-2.7	down	Glycogen phosphorylase, muscle form
Omy#BX304263	BX304263	-2.7	down	1-acyl-sn-glycerol-3-phosphate acyltransferase theta-B
Ssa#STIR25732	TC89676	-2.7	down	---NA---
Ssa#STIR20772	TC82191	-2.7	down	Salmo salar clone ssal-rgf-529-095 G2/mitotic-specific cyclin-B1 putative mRNA, pseudogene cds
Ssa#S32005938	S32005938	-2.7	down	DEP domain containing 1a
Ssa#S30239794	S30239794	-2.7	down	Synaptophysin-like protein 1
Ssa#S35559328	S35559328	-2.7	down	Phosphotriesterase-related protein
Ssa#S35577848	S35577848	-2.7	down	selenoprotein H
Ssa#S31979006	S31979006	-2.7	down	homo sapiens peptidase domain containing associated with muscle regeneration 1 transcript variant mrna
Ssa#DY716184	DY716184	-2.7	down	ADP-ribosylation factor domain protein 1
Ssa#S35594269	S35594269	-2.7	down	Butyrate response factor 2
Omy#CA388099	CA388099	-2.7	down	periostin isoform 1
Ssa#TC97712	TC97712	-2.7	down	rattus norvegicus mrna for type ii brain minor
Ssa#S35515248	S35515248	-2.7	down	Tenomodulin
Ssa#TC102265	TC102265	-2.7	down	Band 4.1-like protein 2
Ssa#STIR04369	gi|209733261	-2.7	down	mannose-binding protein c precursor
Ssa#S31985622_S	S31985622	-2.7	down	FK506-binding protein 10 precursor
Ssa#STIR17002	TC76567	-2.7	down	Salmo salar retinoic acid receptor gamma a (Rarga), coiled-coil transcriptional coactivator a (Kiaa1536),
Ssa#S35582954	S35582954	-2.7	down	tetraodon nigroviridis full-length cdna
Ssa#STIR09537	TC66145	-2.7	down	annexin a2a
Ssa#STIR14574	TC73065	-2.7	down	ebv-induced g-protein coupled receptor 2
Ssa#S35492331	S35492331	-2.7	down	similar to Protein phosphatase 1 regulatory subunit 3D (Protein phosphatase 1, regulatory subunit 6)
Ssa#STIR16244	TC75427	-2.7	down	outer membranefamily
Ssa#STIR16674	TC76064	-2.7	down	Delta3,5-delta2,4-dienoyl-CoA isomerase, mitochondrial precursor
Ssa#EG900061	EG900061	-2.7	down	Ribonucleoside-diphosphate reductase subunit M2
Ssa#S18888849	S18888849	-2.7	down	ATPase family AAA domain-containing protein 2
Ssa#TC108644	TC108644	-2.7	down	tetraodon nigroviridis full-length cdna
Ssa#S35532957	S35532957	-2.7	down	similar to coactivator-associated arginine methyltransferase 1
Ssa#STIR23102	TC85737	-2.7	down	Zebrafish DNA sequence from clone DKEY-54K13 in linkage group 3, complete sequence
Ssa#TC87042	TC87042	-2.7	down	Lumican
Ssa#S30292394_S	S30292394	-2.7	down	similar to uracil-DNA-glycosylase, UNG2
Ssa#S30242757	S30242757	-2.7	down	gasterosteus aculeatus clone cnb45-f04 mrna sequence
Ssa#TC111132	TC111132	-2.7	down	Bmp1a protein
Ssa#CX358351	CX358351	-2.7	down	Integrin beta 3b
Ssa#DY732310	DY732310	-2.7	down	equus caballus histone-lysine n-methyltransferase suv39h1 (suppressor of variegation 3-9 homolog 1)
Ssa#S35484722	S35484722	-2.7	down	C8orf4 homolog
Ssa#S35564035_S	S35564035	-2.7	down	Glutathione peroxidase 7 precursor
Ssa#STIR13879	TC72065	-2.7	down	Zebrafish DNA sequence from clone DKEY-88N24 in linkage group 7 Contains part of the clock3 gene
Ssa#S31964325	S31964325	-2.7	down	serine protease-like protein precursor
Ssa#S32010446	S32010446	-2.7	down	similar to drebrin 1, partial
Ssa#STIR26170	TC90332	-2.7	down	phosphatidylinositol binding clathrin assembly protein
Ssa#STIR14684	TC73227	-2.7	down	---NA---
Ssa#S18888681	S18888681	-2.7	down	Replication factor C subunit 5
Ssa#S30284283	S30284283	-2.7	down	LOC559236 protein

Ssa#STIR13480	TC71487	-2.7	down	acetyl-coenzyme a carboxylase alpha
Ssa#STIR42494	DY719403	-2.7	down	allograft inflammatory factor 1-like
Ssa#STIR40066	TC111065	-2.7	down	novel protein similar to sirtuin (silent mating type information regulation 2 homolog) family
Ssa#STIR18129	TC78228	-2.7	down	matrix-remodelling associated 8
Ssa#STIR02018	gi|209737981	-2.7	down	spindle pole body component 24 homolog
Ssa#STIR20722	TC82113	-2.7	down	transgelin
Ssa#STIR16295	TC75502	-2.7	down	Danio rerio K(lysine) acetyltransferase 2B (kat2b), mRNA
Ssa#STIR19243	TC79886	-2.8	down	tumor necrosis factor receptormember 11a
Ssa#S30295808	S30295808	-2.8	down	gasterosteus aculeatus clone cfw169-h06 mrna sequence
Ssa#S35558804	S35558804	-2.8	down	Plasma retinol-binding protein 1
Ssa#STIR15337	TC74141	-2.8	down	yippee-like 2
Ssa#S35582672	S35582672	-2.8	down	Phosphoserine phosphatase
Ssa#STIR11833	TC69188	-2.8	down	erythroblast membrane-associated protein
Ssa#DW558445	DW558445	-2.8	down	novel protein (wu:fd18f09)
Ssa#STIR22932	TC85467	-2.8	down	CNS0GFGOTetraodon nigroviridis full-length cDNA
Ssa#STIR06520	gi|209154943	-2.8	down	dna replication complex gins protein psf2
Ssa#STIR24719	TC88157	-2.8	down	thymocyte nuclear protein 1
Ssa#KSS4439	KSS4439	-2.8	down	tetraodon nigroviridis full-length cdna
Ssa#S31979774	S31979774	-2.8	down	tetraodon nigroviridis full-length cdna
Ssa#CA041082	CA041082	-2.8	down	similar to Transforming growth factor, beta receptor III
Ssa#CK887115	CK887115	-2.8	down	danio rerio heparan sulfate 3-o-sulfotransferase 1- mrna (cdna clone mgc:194892 image:9038636)
Ssa#STIR11726	TC69048	-2.8	down	---NA---
Ssa#TC112457	TC112457	-2.8	down	scavenger receptor class A, member 5
Ssa#S30264776	S30264776	-2.8	down	DNA polymerase subunit alpha B
Ssa#CA037592	CA037592	-2.8	down	mus musculus myosin ib mrna
Ssa#S30294427	S30294427	-2.8	down	danio rerio leucine-rich repeats and immunoglobulin-like domains 3 mrna
Ssa#S30293322	S30293322	-2.8	down	Perlwapin
Ssa#S35601674	S35601674	-2.8	down	Peroxisomal 3,2-trans-enoyl-CoA isomerase
Ssa#TC110687	TC110687	-2.8	down	macrophage mannose receptor 2
Ssa#KSS3754	NM_001173741	-2.8	down	DNA replication licensing factor mcm4
Omy#TC147205	TC147205	-2.8	down	DNA topoisomerase 2-alpha
Ssa#S35580593	S35580593	-2.8	down	tetraodon nigroviridis full-length cdna
Ssa#S32001187	S32001187	-2.8	down	C-C motif chemokine 25
Ssa#S35473212	S35473212	-2.8	down	Dihydropyrimidinase-related protein 5
Ssa#S31998683	DY727280	-2.8	down	laminin, beta 1
Ssa#S35671757	S35671757	-2.8	down	Extracellular superoxide dismutase
Omy#BX911866	BX911866	-2.8	down	Itgb5 protein
Ssa#S31984154	S31984154	-2.8	down	AF152342_1DNA-(cytosine-5)-methyltransferase
Ssa#S35582886	S35582886	-2.8	down	4-hydroxyphenylpyruvate dioxygenase
Ssa#EG930234	EG930234	-2.8	down	Arachidonate 5-lipoxygenase
Ssa#S35580645	EG867023	-2.8	down	Vascular endothelial growth factor D
Ssa#S18891150	S18891150	-2.8	down	Lymphokine-activated killer T-cell-originated protein kinase homolog
Ssa#CL399Ctg1	CL399Contig1	-2.8	down	Ribonucleoside-diphosphate reductase subunit M2
Ssa#STIR20483	TC81760	-2.8	down	: Gallus gallus fibrillin 1 (FBN1), mRNA
Ssa#STIR08185	TC64457	-2.8	down	Salmo salar EAP30 subunit of ELL complex a (Eap30a), nuclear domain 10 protein 52 a (Ndp52a)
Ssa#S30296276	S30296276	-2.8	down	MOSC domain-containing protein 1, mitochondrial precursor
Ssa#TC104365	TC104365	-2.8	down	Importin subunit alpha-2
Ssa#TC104075	TC104075	-2.8	down	salmo salar clone ssal-rgf-503-195 sam and sh3 domain-containing protein 1 partial cds
Ssa#STIR09261	TC65792	-2.8	down	collagen alpha-2chain precursor
Ssa#STIR14199	TC72515	-2.8	down	---NA---
Ssa#STIR15249	TC74009	-2.8	down	fascin homologactin-bundling protein (strongylocentrotus purpuratus)
Ssa#S30276463	S30276463	-2.8	down	Serine protease 23 precursor
Ssa#TC97955	TC97955	-2.9	down	coiled-coil domain containing 80
Ssa#S35600309	S35600309	-2.9	down	brugia malayi transglutaminase partial cds
Ssa#S35532958	S35532958	-2.9	down	similar to coactivator-associated arginine methyltransferase 1
Ssa#DW565190	DW565190	-2.9	down	homo sapiens sodium voltage- type beta transcript variant mrna
Ssa#STIR25700	TC89640	-2.9	down	Salmo salar clone ssal-rgf-538-026 Band 4.1-like protein 2 putative mRNA
Ssa#STIR13500	TC71516	-2.9	down	---NA---
Ssa#S18889767	S18889767	-2.9	down	NADP-dependent leukotriene B4 12-hydroxydehydrogenase
Ssa#S35584669	S35584669	-2.9	down	Centromere protein H
Ssa#S35563089	EG849467	-2.9	down	similar to alpha 1 (V) collagen
Ssa#CX356053	CX356053	-2.9	down	DNA replication licensing factor mcm5
Ssa#STIR14511	TC72971	-2.9	down	---NA---
Ssa#STIR14183	TC72491	-2.9	down	angiopoietin-like 2
Ssa#S35554287	S35554287	-2.9	down	Extracellular superoxide dismutase
Ssa#DY724176	DY724176	-2.9	down	sestrin 1
Ssa#S30263554	S30263554	-2.9	down	Golgi reassembly-stacking protein 2
Ssa#STIR20337	TC81550	-2.9	down	plasma retinol-binding protein 1
Ssa#STIR21883	TC83891	-2.9	down	pdz and lim domain 2
Ssa#STIR25295	TC89018	-2.9	down	---NA---
Ssa#DY736742	DY736742	-2.9	down	transposase
Ssa#STIR21829	TC83809	-2.9	down	cholinesterase 1
Ssa#TC110206	TC110206	-2.9	down	similar to adipocyte enhancer binding protein 1
Ssa#EG833298	EG833298	-2.9	down	GRB2-related adaptor protein 2
Ssa#KSS1623	KSS1623	-2.9	down	Replication protein A 32 kDa subunit
Ssa#STIR04578	gi|209732839	-2.9	down	fatty acid-bindingheart
Ssa#S35493732	S35493732	-2.9	down	rattus norvegicus matrilin 2 mrna
Omy#S34421663	S34421663	-2.9	down	ligand of numb-protein X 1
Omy#S34312706	S34312706	-2.9	down	odd-skipped related 1
Ssa#STIR12354	TC69901	-2.9	down	mannose-binding protein c precursor
Ssa#KSS4352	KSS4352	-2.9	down	Replication factor C subunit 2
Ssa#STIR23663	TC86591	-2.9	down	---NA---
Ssa#STIR21748	TC83681	-2.9	down	---NA---
Ssa#STIR22203	TC84369	-2.9	down	coiled-coil domain containing 104
Ssa#S35596673	S35596673	-2.9	down	LOC402831 protein
Ssa#STIR12218	TC69715	-2.9	down	Tetraspanin-4
Ssa#S35552129_S	S35552129	-2.9	down	novel protein (zgc:153086)
Ssa#STIR13706	TC71815	-2.9	down	Salmo salar clone ssal-rgf-524-264 unknown large open reading frame mRNA, novel cds
Ssa#S35565631	S35565631	-2.9	down	Amidophosphoribosyltransferase
Ssa#S30246050	DW542111	-2.9	down	glyceraldehyde-3-phosphate dehydrogenase-2
Omy#S15281391	S15281391	-2.9	down	Adenylosuccinate synthetase isozyme 2
Ssa#STIR18648	TC79015	-2.9	down	transmembrane protein 77
Ssa#TC90520	TC90520	-2.9	down	sestrin 1
Ssa#STIR11981	TC69396	-2.9	down	Salmo salar clone ssal-rgf-509-303 Interferon-induced guanylate-binding protein 1 putative mRNA,
Ssa#DY704594	DY704594	-2.9	down	exonuclease 1
Ssa#S30285768	S30285768	-2.9	down	Calcium/calmodulin-dependent protein kinase II inhibitor 2

Ssa#DY693975	DY693975	-2.9	down	Olfactomedin-like protein 2A precursor
Ssa#STIR04649	gi|209732693	-2.9	down	glutathione peroxidase 7
Ssa#S35559341	S35559341	-3.0	down	periostin isoform 1
Ssa#S48397792	S48397792	-3.0	down	HGV2
Ssa#S31984473	S31984473	-3.0	down	Segment polarity protein dishevelled homolog DVL-3
Ssa#S30242093	S30242093	-3.0	down	---NA---
Ssa#STIR04709	gi|209732573	-3.0	down	n-acetyltransferase 8-like protein
Ssa#STIR03729	gi|209734541	-3.0	down	fk506-binding protein 11 precursor
Ssa#STIR35406	TC104251	-3.0	down	CXC chemokine d1
Ssa#S31987995	S31987995	-3.0	down	tetraodon nigroviridis full-length cdna
Ssa#STIR23127	TC85769	-3.0	down	---NA---
Ssa#S32012003	S32012003	-3.0	down	60 kDa lysophospholipase
Ssa#TC103697_S	TC103697	-3.0	down	DNA replication licensing factor mcm2
Ssa#S35705996	S35705996	-3.0	down	ADP-ribosylation factor-like protein 4C
Ssa#DY729436	DY729436	-3.0	down	similar to Pde7a protein
Ssa#S30263209	S30263209	-3.0	down	: HtrA serine peptidase 3
Ssa#STIR21650	TC83542	-3.0	down	Salmo salar clone ssal-rgf-501-010 unknown large open reading frame mRNA, novel cds
Omy#S34424010	S34424010	-3.0	down	novel protein similar to vertebrate integrin, beta-like 1 (with EGF-like repeat domains) (ITGBL1, zgc:112304)
Ssa#S18869280	S18869280	-3.0	down	Cytosolic sulfotransferase 2
Ssa#STIR10049	TC66782	-3.0	down	---NA---
Ssa#STIR21410	TC83184	-3.0	down	excision repair cross-complementing rodent repaircomplementation group 6-like
Ssa#S35701995	S35701995	-3.0	down	Replication protein A 32 kDa subunit
Ssa#S30284087	S30284087	-3.0	down	Short-chain dehydrogenase/reductase 3
Ssa#S30292394	S30292394	-3.0	down	similar to uracil-DNA-glycosylase, UNG2
Ssa#S35563409	S35563409	-3.0	down	similar to integrin, alpha E (antigen CD103, human mucosal lymphocyte antigen 1; alpha polypeptide)
Ssa#S31985622	S31985622	-3.0	down	FK506-binding protein 10 precursor
Ssa#STIR00115_4	gi|209155893	-3.0	down	tropomyosin-1 alpha chain
Ssa#STIR18918	TC79407	-3.0	down	proline arginine-rich end leucine-rich repeat protein
Ssa#STIR04788	gi|209732413	-3.0	down	cxc chemokine d1
Ssa#S18844587	S18844587	-3.0	down	Metallothionein
Ssa#S31988940	S31988940	-3.0	down	Transforming growth factor beta-1 precursor
Ssa#S48441015_S	S48441015	-3.0	down	contractile ring component anillin
Ssa#TC103697	TC103697	-3.0	down	DNA replication licensing factor mcm2
Ssa#STIR18045	TC78109	-3.0	down	pt repeat family protein
Ssa#STIR14265	TC72601	-3.0	down	CNS0GEULTetraodon nigroviridis full-length cDNA
Ssa#STIR11579	TC68852	-3.0	down	Salmo salar clone ssal-rgf-531-250 Circumsporozoite protein precursor putative mRNA, pseudogene cds
Ssa#STIR11224	TC68370	-3.0	down	cyclin-dependent kinase 4
Ssa#STIR16362	TC75597	-3.0	down	Salmo salar retinoic acid receptor gamma a (Rarga), coiled-coil transcriptional coactivator a (Kiaa1536),
Omy#S15322001	S15322001	-3.0	down	tetraodon nigroviridis full-length cdna
Ssa#CK890148	CK890148	-3.0	down	salmo salar clone ssal-rgf-523-177 mcm10 homolog pseudogene cds
Ssa#STIR12008	TC69433	-3.0	down	cell division cycle 2
Ssa#S30283213	S30283213	-3.0	down	leprecan 1
Ssa#S31987829	S31987829	-3.0	down	Wu:fc51g12 protein
Ssa#STIR24952	TC88507	-3.0	down	: Danio rerio wu:fa99c11 (wu:fa99c11), partial mRNA
Ssa#STIR43195_S	CB510634	-3.0	down	unnamed protein product
Ssa#EG898520	EG898520	-3.0	down	zebrafish dna sequence from clone dkey-204a24 in linkage group 19 contains aquaporin 3
Ssa#S18891161	S18891161	-3.0	down	DIX domain containing 1
Ssa#S35501264	S35501264	-3.0	down	DNA replication licensing factor mcm4
Ssa#CA056364	CA056364	-3.0	down	ATPase, Na+/K+ transporting, beta 2b polypeptide
Ssa#S35708455	S35708455	-3.0	down	Catalase
Ssa#S35585751	S35585751	-3.1	down	salmo salar s100 calcium binding protein v2-like mrna
Ssa#STIR21980	TC84039	-3.1	down	replication protein32kda
Omy#S15338110	S15338110	-3.1	down	---NA---
Ssa#STIR14931	TC73568	-3.1	down	n-acetyltransferase 8-like protein
Ssa#S18852573	S18852573	-3.1	down	HIRA-interacting protein 3
Ssa#S35552704	S35552704	-3.1	down	Heme-binding protein 2
Ssa#TC103621	TC103621	-3.1	down	potassium voltage-gated channel subfamily H member 2
Ssa#S31977813	DY706603	-3.1	down	similar to myosin phosphatase-Rho interacting protein
Ssa#S35549506	S35549506	-3.1	down	Hypoxanthine-guanine phosphoribosyltransferase
Ssa#S35590423	S35590423	-3.1	down	takifugu rubripes a gene complete sequence
Ssa#STIR17500	TC77306	-3.1	down	Danio rerio cell adhesion molecule-related/down-regulated by oncogenes,
Ssa#S30281464	S30281464	-3.1	down	origin recognition complex subunit 5
Ssa#STIR39152_S	TC109730	-3.1	down	: novel protein similar to vertebrate phosphatidic acid phosphatase type 2B (PPAP2B)
Ssa#S48402144_S	S48402144	-3.1	down	similar to TFP250
Ssa#STIR23837	TC86839	-3.1	down	---NA---
Ssa#S35550197	S35550197	-3.1	down	rattus norvegicus huntingtin interacting protein 1 related transcript variant mrna
Ssa#STIR25907	TC89949	-3.1	down	Salmo salar clone BAC CHORI214-424M17, complete sequence
Omy#CA360668	CA360668	-3.1	down	similar to platelet endothelial aggregation receptor 1
Ssa#STIR26185	TC90356	-3.1	down	polyglutamine-containing protein
Ssa#STIR25506	TC89337	-3.1	down	type i keratin s8
Ssa#DW181344_S	DW181344	-3.1	down	Insulin-like growth factor-binding protein 7 precursor
Ssa#S35668599	S35668599	-3.1	down	Coronin-1A
Ssa#STIR25879	TC89906	-3.1	down	rad51 homolog
Ssa#STIR43670	DW560870	-3.1	down	Histone chaperone asf1-A
Omy#S15286726	S15286726	-3.1	down	hypothetical protein BRAFLDRAFT_112737
Ssa#S30271914	S30271914	-3.1	down	danio rerio zgc:73290 (zgc:73290) mrna
Ssa#STIR07483	TC63641	-3.1	down	M.truncatula DNA sequence from clone MTH2-59B18 on chromosome 3, complete sequence
Ssa#S32001395	S32001395	-3.1	down	hypothetical protein LOC379996
Ssa#STIR00070_4	CK888080	-3.1	down	interferon-induced transmembrane protein
Ssa#S35546719	S35546719	-3.1	down	Cyclin-A2
Ssa#STIR04006	gi|209733987	-3.1	down	heterogeneous nuclear ribonucleoprotein l
Ssa#EG757865	EG757865	-3.1	down	salmo salar tetraspanin-18 mrna
Ssa#STIR18008	TC78045	-3.1	down	fatty acid-bindingheart
Ssa#S30283070	S30283070	-3.1	down	Tropomyosin-1 alpha chain
Ssa#S35507591	S35507591	-3.1	down	similar to ADAMTS-8 precursor (A disintegrin and metalloproteinase with thrombospondin)
Omy#S18155141	S18155141	-3.1	down	novel protein similar to H.sapiens EFEMP1, EGF-containing fibulin-like extracellular matrix protein 1
Ssa#STIR16357	TC75591	-3.1	down	Mus musculus transcription factor AP4 (Tcfap4), mRNA
Ssa#STIR15515	TC74382	-3.1	down	replication protein32kda
Ssa#STIR08890	TC65315	-3.1	down	novel protein
Ssa#S33389632	S33389632	-3.1	down	tyrosinase
Ssa#STIR15271	TC74039	-3.2	down	fumarylacetoacetate hydrolase
Ssa#S32007565	S32007565	-3.2	down	Ribonucleoside-diphosphate reductase large subunit
Ssa#S18849150	S18849150	-3.2	down	Histone H2A.x
Ssa#STIR07270	gi|209148650	-3.2	down	dimethylarginine dimethylaminohydrolase 2
Ssa#DY709425	DY709425	-3.2	down	Zygotic DNA replication licensing factor mcm6-B
Ssa#S35590559	S35590559	-3.2	down	tetraodon nigroviridis full-length cdna

Ssa#STIR13615	TC71682	-3.2	down	fk506-binding protein 11 precursor
Ssa#STIR17042	TC76626	-3.2	down	phosphoserine phosphatase
Ssa#S30260329	S30260329	-3.2	down	similar to Ras GTPase-activating-like protein IQGAP3
Ssa#STIR21066	TC82653	-3.2	down	Salmo salar clone ssal-eve-535-325 Centromere protein M putative mRNA,
Ssa#S30290620	DW577584	-3.2	down	cyclin-dependent kinase 4
Ssa#S35554388	S35554388	-3.2	down	Tropomyosin-1 alpha chain
Ssa#STIR22192	TC84357	-3.2	down	---NA---
Ssa#STIR00070_3	CK888080	-3.2	down	interferon-induced transmembrane protein
Ssa#STIR09911	TC66604	-3.2	down	chromosome 9 open reading frame 119
Ssa#S35535473_S	S35535473	-3.2	down	Neuropilin-2
Ssa#STIR13732	TC71853	-3.2	down	Salmo salar clone ssal-rgf-524-155 C20orf27 putative mRNA,
Ssa#STIR23334	TC86090	-3.2	down	Salmo salar EAP30 subunit of ELL complex a (Eap30a),HoxB5ba (HoxB5ba),
Ssa#STIR21075	TC82663	-3.2	down	complement c1q tumor necrosis factor-related protein 5 precursor
Ssa#CA056843_S	CA056843	-3.2	down	similar to Probable E3 ubiquitin-protein ligase HECTD2 (HECT domain-containing protein 2)
Ssa#STIR02236	gi|209737543	-3.2	down	histone
Ssa#STIR17524	TC77340	-3.2	down	fatty acid-bindingheart
Ssa#STIR36175	TC105375	-3.2	down	similar to RAB38
Omy#S22932617	S22932617	-3.2	down	fructose-1,6-bisphosphatase
Ssa#S35490761_S	S35490761	-3.2	down	Transcription factor HES-1
Ssa#EG774127	EG774127	-3.2	down	sus scrofa clone: expressed in ovary
Ssa#S31973974	S31973974	-3.2	down	novel protein similar to vertebrate sestrin 1 (SESN1)
Ssa#S35659755	S35659755	-3.2	down	kinesin family member 18A
Ssa#DW544962	DW544962	-3.2	down	DNA replication licensing factor mcm2
Ssa#STIR23811	TC86800	-3.2	down	---NA---
Ssa#STIR20087	TC81177	-3.2	down	Plasmodium falciparum 3D7 chromosome 2, complete sequence
Ssa#TC110815	TC110815	-3.2	down	similar to Podocan
Omy#TC141710	TC141710	-3.2	down	Protein-glutamine gamma-glutamyltransferase K
Ssa#STIR12856	TC70610	-3.2	down	activated rna polymerase ii transcriptional coactivator p15
Ssa#STIR15662	TC74588	-3.2	down	Salmo salar clone ssal-rgf-540-055 ERBB receptor feedback inhibitor 1 putative mRNA,
Ssa#DW574440	DW574440	-3.3	down	similar to Peroxisomal sarcosine oxidase (PSO) (L-pipecolate oxidase) (L-pipecolic acid oxidase)
Ssa#STIR11632	TC68920	-3.3	down	---NA---
Ssa#STIR31340	TC98204	-3.3	down	Adenosine deaminase
Ssa#S35693513	EG928231	-3.3	down	Complement component C6 precursor
Omy#TC137323	TC137323	-3.3	down	gasterosteus aculeatus clone cnb110-b08 mrna sequence
Ssa#STIR07847	TC64064	-3.3	down	proliferating cell nuclear antigen
Ssa#TC65490	TC65490	-3.3	down	F11 receptor
Ssa#S31985316	S31985316	-3.3	down	hyaluronan mediated motility receptor
Ssa#STIR15525	TC74394	-3.3	down	CNS0FSUVTetraodon nigroviridis full-length cDNA
Ssa#STIR09222	TC65741	-3.3	down	cytokine-like protein 1 precursor
Ssa#S35535870	S35535870	-3.3	down	hypothetical protein LOC566969
Ssa#KSS396	KSS396	-3.3	down	Nicotinamide riboside kinase 2
Ssa#EG900061_S	EG900061	-3.3	down	Ribonucleoside-diphosphate reductase subunit M2
Ssa#TC111124	TC111124	-3.3	down	integrin beta 3b
Ssa#STIR18535	TC78843	-3.3	down	sh2 domain containing 3c
Ssa#STIR22903	TC85424	-3.3	down	phosphoinositide-3-kinase interacting protein 1
Ssa#S35478387	S35478387	-3.3	down	meiosis-specific nuclear structural protein 1
Ssa#S32004381	S32004381	-3.3	down	DNA ligase I
Ssa#STIR21835	TC83818	-3.3	down	serine peptidasekazal type 2 (acrosin-trypsin inhibitor)
Ssa#DY699849	DY699849	-3.3	down	ankyrin repeat domain 29
Omy#S18157661	S18157661	-3.3	down	Pterin-4-alpha-carbinolamine dehydratase
Ssa#S35659383	EG894101	-3.3	down	Cyclin-A2
Ssa#S35657190	S35657190	-3.3	down	Dihydrofolate reductase
Ssa#STIR22294	TC84501	-3.3	down	Salmo salar clone ssal-rgf-541-083 unknown large open reading frame mRNA, novel cds
Omy#CB498037	CB498037	-3.3	down	Alpha-1,6-mannosyl-glycoprotein 2-beta-N-acetylglucosaminyltransferase
Ssa#S35581420	S35581420	-3.3	down	SWI/SNF related, matrix associated, actin dependent regulator of chromatin, subfamily a, member 1
Ssa#STIR05587	gi|209730803	-3.3	down	chromosome 9 open reading frame 119
Ssa#S35589555	S35589555	-3.3	down	Dnmt1 protein
Ssa#STIR06929	gi|209154119	-3.3	down	heterochromatin proteinbinding protein 3
Ssa#TC104791	TC104791	-3.3	down	Ras association domain-containing protein 4
Ssa#TC105363_S	TC105363	-3.3	down	Transposable element Tcb1 transposase
Omy#CA385715	CA385715	-3.4	down	Zygotic DNA replication licensing factor mcm6-B
Ssa#STIR16777	TC76228	-3.4	down	---NA---
Ssa#DW536138	DW536138	-3.4	down	Zygotic DNA replication licensing factor mcm6-B
Ssa#S32012724	S32012724	-3.4	down	Fibroleukin
Omy#S18162346	S18162346	-3.4	down	hypothetical protein LOC559560
Ssa#S35532747	S35532747	-3.4	down	: si:ch211-198b3.2
Ssa#EG880400	EG880400	-3.4	down	envelope polyprotein
Ssa#S35693115	S35693115	-3.4	down	type I keratin S8
Omy#BX862125	BX862125	-3.4	down	similar to Williams-Beuren syndrome chromosome region 24 homolog
Omy#S18098286	S18098286	-3.4	down	K0841 protein
Ssa#STIR23107	TC85742	-3.4	down	Oncorhynchus mykiss collectin sub-family member 12 mRNA
Ssa#STIR13784	TC71927	-3.4	down	novel protein
Ssa#S46924879	EU861009.1	-3.4	down	insulin-like growth factor binding protein 5
Ssa#STIR19460	TC80227	-3.4	down	Danio rerio aryl hydrocarbon receptor nuclear translocator-like 2,
Ssa#STIR19758	TC80684	-3.4	down	Oncorhynchus mykiss cyp19b-I gene for P450aromB-I, exons 1-10
Ssa#STIR09196	TC65708	-3.4	down	: Danio rerio misc_RNA (LOC556254), miscRNA
Ssa#CB502155	CB502155	-3.4	down	hypothetical protein LOC100002993
Ssa#S35606171	S35606171	-3.4	down	HGV2
Ssa#STIR20591	TC81908	-3.4	down	flj00269 protein
Ssa#STIR18253	TC78388	-3.4	down	---NA---
Ssa#STIR24071	TC87204	-3.4	down	Salmo salar clone ssal-rgf-508-383 Histone deacetylase 3 putative mRNA,
Ssa#TC69118	TC69118	-3.4	down	zebrafish dna sequence from clone dkey-204a24 in linkage group 19 contains aquaporin 3
Ssa#STIR15762	TC74742	-3.4	down	Zebrafish DNA sequence from clone DKEYP-69H1 in linkage group 17, complete sequence
Ssa#STIR25385	TC89151	-3.4	down	Pan troglodytes BAC clone RP43-31J11 from chromosome 7, complete sequence
Ssa#CA040320	CA040320	-3.4	down	tetraodon nigroviridis full-length cdna
Ssa#EG826789	EG826789	-3.4	down	F11 receptor
Ssa#S35702782	S35702782	-3.4	down	DNA replication complex GINS protein SLD5
Ssa#S31991586	S31991586	-3.4	down	novel protein similar to vertebrate retinol dehydrogenase 13 (all-trans and 9-cis) (RDH13)
Ssa#S35585629	S35585629	-3.5	down	similar to Epiplakin
Ssa#S35582070	S35582070	-3.5	down	tetraodon nigroviridis full-length cdna
Ssa#S35574435	S35574435	-3.5	down	tetraodon nigroviridis full-length cdna
Ssa#STIR25087	TC88711	-3.5	down	budding uninhibited by benzimidazoles 1beta
Ssa#STIR10369	TC67207	-3.5	down	gins complex subunit 4 (sld5 homolog)
Ssa#TC87898	TC87898	-3.5	down	sestrin 3
Ssa#STIR36692	TC106159	-3.5	down	danio rerio adipocyte enhancer binding protein 1 mrna
Ssa#S31991594	S31991594	-3.5	down	chromatin assembly factor 1, subunit A

Ssa#S32004569	DY733166	-3.5	down	corticotropin releasing factor precursor
Ssa#STIR20652	TC82002	-3.5	down	---NA---
Ssa#STIR08311	TC64625	-3.5	down	complement factor d
Ssa#S35670029	S35670029	-3.5	down	CI116 protein
Ssa#STIR18906	TC79386	-3.5	down	Salmo salar clone Alu354 microsatellite sequence
Ssa#S18891023	S18891023	-3.5	down	mutS homolog 6 (E. coli)
Ssa#STIR21734	TC83662	-3.5	down	agmatine ureohydrolase
Ssa#STIR05529	BT046528	-3.5	down	collagen triple helix repeat containing 1
Ssa#S35492090_S	S35492090	-3.5	down	Transcription factor HES-1
Ssa#S35679988	S35679988	-3.5	down	Activated RNA polymerase II transcriptional coactivator p15
Ssa#S35485236	S35485236	-3.5	down	mus musculus titin transcript variant n2- mrna
Ssa#STIR02008	gi|209738001	-3.5	down	gins complex subunit 4 (sld5 homolog)
Ssa#STIR24654	TC88061	-3.5	down	---NA---
Ssa#STIR08660	TC65031	-3.5	down	---NA---
Ssa#S18836920	S18836920	-3.6	down	RAD51-associated protein 1
Omy#S34422238	S34422238	-3.6	down	protein-O-mannosyltransferase 2
Ssa#STIR10061	TC66797	-3.6	down	Salmo salar clone ssal-rgf-519-117 Catalase putative mRNA,
Ssa#STIR11086	TC68169	-3.6	down	lunatic fringe
Ssa#S31973823	S31973823	-3.6	down	danio rerio mrna for f-
Ssa#S35591248	S35591248	-3.6	down	salmo salar clone 272p16 chaperonin myosin 1 partial cds and tcr-gamma partial sequence
Ssa#S35559039	S35559039	-3.6	down	Transforming growth factor-beta-induced protein ig-h3
Ssa#STIR16805	TC76268	-3.6	down	---NA---
Ssa#S31979451	S31979451	-3.6	down	MAL2
Ssa#S30272774	S30272774	-3.6	down	glycosyltransferase
Ssa#S35604393	S35604393	-3.6	down	salmo salar clone ssal-rgf-510-110 histone h1- late embryonic
Omy#S34315176	S34315176	-3.6	down	Fumarylacetoacetase
Ssa#STIR19131	TC79718	-3.6	down	coiled-coil domain containing 80
Ssa#S35702222	S35702222	-3.6	down	72 kDa type IV collagenase precursor
Omy#S18159333	S18159333	-3.6	down	osmerus mordax clone omor-eva-514-090 microsomal glutathione s-transferase 1
Ssa#STIR16123	TC75248	-3.6	down	---NA---
Ssa#TC112853	TC112853	-3.6	down	Helicase, lymphoid-specific
Ssa#S31987273	S31987273	-3.6	down	Proliferating cell nuclear antigen
Ssa#S18850563	S18850563	-3.6	down	similar to fucokinase
Ssa#STIR20648	TC81993	-3.7	down	eph receptor b4a
Ssa#STIR07259	gi|209148870	-3.7	down	agmatine ureohydrolase
Ssa#S18888702	S18888702	-3.7	down	novel protein similar to vertebrate proline rich region 18 (PRR18)
Ssa#STIR03625	gi|209734749	-3.7	down	fatty acid-bindingheart
Ssa#CA040972	CA040972	-3.7	down	---NA---
Ssa#STIR13509	TC71530	-3.7	down	Salmo salar clone ssal-rgf-503-023 Spindlin-Z putative mRNA,
Omy#S18536654	S18536654	-3.7	down	DNA topoisomerase 2-alpha
Ssa#S35505093	S35505093	-3.7	down	geminin, DNA replication inhibitor
Ssa#DW579184	DW579184	-3.7	down	Antigen KI-67
Ssa#S30258629	S30258629	-3.7	down	novel protein similar to vertebrate protein tyrosine phosphatase, receptor-type,
Omy#S18142577	S18142577	-3.7	down	Microsomal glutathione S-transferase 1
Ssa#KSS3361	KSS3361	-3.7	down	60 kDa lysophospholipase
Ssa#S35545872	S35545872	-3.7	down	Development and differentiation-enhancing factor-like 1
Ssa#STIR13241	TC71131	-3.7	down	: Rattus norvegicus similar to ARG99 homolog () (RGD1564868_), mRNA
Ssa#STIR24966	TC88526	-3.7	down	ubiquitin-containing phd and ring finger1
Ssa#STIR09058	TC65530	-3.7	down	collagen-like surface-anchored protein
Ssa#TC69403	TC69403	-3.7	down	Selenoprotein P, plasma, 1a
Ssa#STIR19939	TC80967	-3.7	down	adipocyte enhancer binding protein 1
Ssa#STIR21682	TC83592	-3.7	down	Salmo salar clone ssal-rgf-528-098 Pigment epithelium-derived factor precursor putative mRNA,
Ssa#STIR13835	TC72003	-3.7	down	---NA---
Ssa#TC108704	TC108704	-3.8	down	Lipocalin precursor
Ssa#STIR00161_3	gi|209155391	-3.8	down	cytochrome p450 1a
Ssa#S35668716	S35668716	-3.8	down	Fibroleukin
Ssa#STIR22647	TC85048	-3.8	down	spindle pole body component 24 homolog
Ssa#STIR19681	TC80561	-3.8	down	---NA---
Ssa#S31992074	DY720671	-3.8	down	NP95
Ssa#STIR12399	TC69964	-3.8	down	: Macaca mulatta similar to collagen, type XXIV, alpha 1, transcript variant 1 (LOC711713), mRNA
Ssa#S35665613	S35665613	-3.8	down	PCNA-associated factor
Ssa#TC85331	TC85331	-3.8	down	Zygotic DNA replication licensing factor mcm6-B
Omy#S19715372	S19715372	-3.8	down	glycinamide ribonucleotide synthetase-aminoimidazole
Ssa#STIR17370	TC77120	-3.8	down	Salmo salar clone ssal-rgf-540-094 Follistatin-related protein 1 precursor putative mRNA,
Ssa#S31995578	S31995578	-3.8	down	similar to sestrin 1
Ssa#STIR09299	TC65840	-3.8	down	---NA---
Ssa#S32009403	S32009403	-3.8	down	Regulating synaptic membrane exocytosis 2
Ssa#S35475017	S35475017	-3.9	down	DNA ligase I
Ssa#STIR10639	TC67563	-3.9	down	Zebrafish DNA sequence from clone CH211-195H14 in linkage group 15, complete sequence
Ssa#STIR15968	TC75028	-3.9	down	novel protein
Ssa#S35697231	S35697231	-3.9	down	Thymidylate synthase
Ssa#S30242447	NM_001141717	-3.9	down	Serine protease HTRA1
Ssa#STIR13906	TC72101	-3.9	down	Plasmodium yoelii yoelii str. 17XNL hypothetical protein (PY03955) partial mRNA
Ssa#STIR25620	TC89513	-3.9	down	microsomal glutathione s-transferase 1
Ssa#S30235658	S30235658	-3.9	down	Cysteine dioxygenase type 1
Ssa#STIR16274	TC75470	-3.9	down	Danio rerio ladybird homeobox homolog 2 (Drosophila), mRNA (cDNA clone MGC:92170 IMAGE:7050746),
Ssa#STIR22386	TC84641	-3.9	down	transcription factor 7-like 1b (t-cellhmg-box)
Ssa#STIR17711	TC77603	-3.9	down	Salmo salar clone ssal-rgf-534-154 Serine protease 23 precursor putative mRNA,
Ssa#STIR13580	TC71633	-3.9	down	acid phosphatase-like 2
Ssa#STIR11340	TC68529	-4.0	down	---NA---
Ssa#STIR26181	TC90347	-4.0	down	---NA---
Ssa#S30286799	S30286799	-4.0	down	epithelial calcium channel
Ssa#S18889554	S18889554	-4.0	down	Ribonucleoside-diphosphate reductase subunit M2
Ssa#TC84312	TC84312	-4.0	down	Tescalcin
Ssa#STIR25996	TC90072	-4.0	down	Salmo salar clone ssal-rgf-515-125 unc-119 homolog putative mRNA,
Ssa#CK891865	CK891865	-4.0	down	similar to Integrin alpha-10
Ssa#STIR07065	gi|209153291	-4.0	down	phosphoinositide-3-kinase interacting protein 1
Ssa#EG855574	EG855574	-4.0	down	retinal pigment epithelium-specific protein 65kDa
Ssa#DW536090	DW536090	-4.0	down	mus musculus olfactomedin 1 transcript variant mrna
Ssa#S35657852	S35657852	-4.0	down	Homologous-pairing protein 2 homolog
Ssa#STIR22306	TC84520	-4.0	down	---NA---
Ssa#KSS5001	KSS5001	-4.0	down	DNA primase large subunit
Ssa#STIR03040	gi|209735923	-4.0	down	microfibrillar-associated protein 2
Ssa#STIR12857	TC70611	-4.0	down	Salmo salar clone ssal-rgf-522-131 unknown large open reading frame mRNA, novel cds
Ssa#S35588603	S35588603	-4.0	down	zebrafish dna sequence from clone ch211-22c18 in linkage group complete sequence
Ssa#STIR24338	TC87581	-4.0	down	Salmo salar clone ssal-rgf-519-327 THUMP domain-containing protein 1 putative mRNA

Ssa#STIR19409	TC80149	-4.0	down	---NA---
Ssa#STIR22352	TC84586	-4.1	down	f-box protein 5
Ssa#STIR15340_S	TC74146	-4.1	down	Exostosin-1c
Ssa#STIR21339	TC83079	-4.1	down	---NA---
Ssa#S35554395	S35554395	-4.1	down	Reprimo-like protein
Ssa#S30268201	S30268201	-4.1	down	NF-kappa-B inhibitor-like protein 2
Omy#TC139844	TC139844	-4.1	down	Ribonucleoside-diphosphate reductase large subunit
Ssa#STIR13365	TC71308	-4.1	down	Zebrafish DNA sequence from clone DKEY-250M6 in linkage group
Ssa#S35698783	S35698783	-4.1	down	CD80-like protein
Con_FLINER_02	gi|209155893|gb|BT045	-4.1	down	---NA---
Ssa#S42501031	S42501031	-4.1	down	paired-like homeodomain transcription factor 3alpha
Ssa#S31963152	S31963152	-4.1	down	: centromere protein I
Ssa#STIR20907	TC82391	-4.1	down	c-c motif chemokine 25 precursor
Ssa#STIR22324	TC84543	-4.1	down	Salmo salar clone ssal-rgf-536-260 Tropomyosin-1 alpha chain putative mRNA,
Ssa#S35570017	S35570017	-4.1	down	mab-21-like 2
Ssa#S35674899	S35674899	-4.1	down	Centromere protein M
Ssa#S35661017	S35661017	-4.2	down	Xanthine dehydrogenase/oxidase
Ssa#S30281512	S30281512	-4.2	down	mannan-binding lectin H3
Ssa#STIR00115_2	gi|209155893	-4.2	down	tropomyosin-1 alpha chain
Ssa#STIR13917	TC72116	-4.2	down	glutathione peroxidase 7
Ssa#STIR15094	TC73786	-4.2	down	zinc finger protein 521
Omy#S15239282	S15239282	-4.2	down	: sc:d0375
Ssa#STIR13690	TC71791	-4.2	down	Salmo salar clone ssal-rgf-506-281 Phosphoinositide-3-kinase-interacting protein 1 precursor putative mRNA,
Ssa#STIR02135	gi|209737747	-4.2	down	novel protein
Ssa#S35675880	S35675880	-4.2	down	Cysteine and glycine-rich protein 2
Ssa#S42501030	S42501030	-4.2	down	paired-like homeodomain transcription factor 3beta
Ssa#TC91867	TC91867	-4.2	down	Col11a1 protein
Ssa#S30268668	S30268668	-4.2	down	MIS12 homolog
Ssa#S18888540	CB514505	-4.2	down	DNA replication licensing factor mcm2
Ssa#STIR24435	TC87723	-4.2	down	transmembrane channel-like 6
Ssa#STIR13687	TC71788	-4.2	down	fk506 binding protein 10
Ssa#S18887181	S18887181	-4.2	down	LOC560459 protein
Ssa#EG893280	EG893280	-4.2	down	S-phase kinase-associated protein 2 (p45)
Ssa#STIR00070_2	CK888080	-4.2	down	interferon-induced transmembrane protein
Ssa#STIR09192	TC65704	-4.3	down	microfibrillar-associated protein 2
Ssa#STIR05187	gi|209731607	-4.3	down	microfibrillar-associated protein 2
Ssa#S35605437	S35605437	-4.3	down	N-acetyltransferase ESCO1
Ssa#STIR12045	TC69481	-4.3	down	secreted frizzled-related protein 1
Ssa#STIR20556	TC81852	-4.3	down	---NA---
Ssa#S35490606	S35490606	-4.3	down	adiponectin, C1Q and collagen domain containing, like
Ssa#STIR14198	TC72513	-4.3	down	---NA---
Ssa#TC81118	TC81118	-4.3	down	glycosyltransferase 8 domain containing 4
Ssa#STIR12248	TC69762	-4.3	down	---NA---
Ssa#TC90430	TC90430	-4.3	down	Helicase, lymphoid-specific
Ssa#STIR18859	TC79313	-4.3	down	TSA: Hippoglossus hippoglossus all_halibut.1645.C1 mRNA sequence
Ssa#CN181393	CN181393	-4.3	down	novel protein
Ssa#STIR16894	TC76410	-4.3	down	kinesin family member 23
Ssa#STIR12785	TC70506	-4.3	down	---NA---
Ssa#DW562425	DW562425	-4.3	down	Type III iodothyronine deiodinase
Ssa#STIR05565	gi|209730847	-4.3	down	fatty acid-bindingheart
Ssa#STIR02869	gi|209736267	-4.3	down	membrane-spanning 4-subfamilymember
Ssa#S24663744	S24663744	-4.3	down	prolactin-releasing peptide C-RFamide precursor
Ssa#STIR05491	gi|209730995	-4.3	down	fatty acid-bindingheart
Ssa#S18883101	S18883101	-4.4	down	Short-chain dehydrogenase/reductase 3
Ssa#STIR19285	TC79951	-4.4	down	---NA---
Ssa#S35708477	S35708477	-4.4	down	similar to galactosidase, beta 1-like 2
Ssa#STIR17576	TC77416	-4.4	down	fMet-Leu-Phe receptor
Ssa#STIR16517	TC75830	-4.4	down	---NA---
Ssa#S35699881	EG934599	-4.4	down	DNA replication licensing factor MCM3
Omy#S34424459	S34424459	-4.4	down	Lumican
Ssa#STIR25269	TC88981	-4.4	down	Salmo salar clone ssal-eve-531-071 Non-histone chromosomal protein H6 putative mRNA,
Ssa#STIR11720	TC69038	-4.4	down	---NA---
Ssa#DY720414	DY720414	-4.4	down	fascin 1
Ssa#S35585716	S35585716	-4.4	down	Secretagogin
Ssa#STIR12238	TC69748	-4.4	down	---NA---
Ssa#S35705881	S35705881	-4.4	down	DNA-directed DNA polymerase epsilon 4
Ssa#S35500885	S35500885	-4.4	down	Transcription factor 15
Ssa#DW472553	DW472553	-4.4	down	similar to alkB, alkylation repair homolog 2
Ssa#STIR10219	TC67006	-4.4	down	claudin 5
Ssa#S35575847	S35575847	-4.5	down	---NA---
Ssa#STIR15009	TC73675	-4.5	down	Mouse DNA sequence from clone DN-383O10 Contains the Zp3r gene for zona pellucida 3 receptor
Ssa#STIR14837	TC73438	-4.5	down	---NA---
Ssa#STIR03173	gi|209735657	-4.5	down	fatty acid-bindingheart
Ssa#STIR12464	TC70061	-4.5	down	Salmo salar clone ssal-rgf-530-028 Phosphoinositide-3-kinase-interacting protein 1 precursor putative mRNA,
Ssa#S32371367	S32371367	-4.5	down	myogenic regulatory factor 5
Ssa#S30281240	S30281240	-4.5	down	Neuroblastoma suppressor of tumorigenicity 1
Ssa#S30296277_S	S30296277	-4.5	down	MOSC domain-containing protein 1, mitochondrial precursor
Ssa#STIR06230	gi|209155529	-4.5	down	phosphoinositide-3-kinase interacting protein 1
Ssa#STIR22226	TC84403	-4.6	down	uracil-dna glycosylase
Ssa#STIR21541	TC83385	-4.6	down	eg667929 protein
Omy#S34309742	S34309742	-4.6	down	tetraodon nigroviridis full-length cdna
Ssa#S35504964	EG791342	-4.6	down	Troponin I, slow skeletal muscle
Ssa#STIR14649	TC73174	-4.6	down	osteocalcin
Ssa#TC86888	TC86888	-4.6	down	homo sapiens regulating synaptic membrane exocytosis 2 transcript variant mrna
Ssa#S32009116	S32009116	-4.6	down	DNA-damage-inducible transcript 4-like
Ssa#STIR23101	TC85736	-4.7	down	AC005181Homo sapiens chromosome 17, clone hRPK.1003_J_3, complete sequence
Ssa#S35687715_S	S35687715	-4.7	down	Complement factor D precursor
Ssa#DY723243	DY723243	-4.7	down	homo sapiens adducin 2 transcript variant beta- mrna
Ssa#S31973744	S31973744	-4.7	down	: wu:fi38h09
Ssa#STIR21270	TC82965	-4.7	down	dna-damage-inducible transcript 4-like
Ssa#S30278680	S30278680	-4.7	down	novel protein similar to vertebrate cell division cycle associated 7 (CDCA7, zgc:110113)
Ssa#STIR14645	TC73170	-4.7	down	Zebrafish DNA sequence from clone DKEY-30I6 in linkage group 25, complete sequence
Ssa#S35532608	S35532608	-4.7	down	similar to RecQ protein-like 4
Ssa#DY736739	DY736739	-4.7	down	Proliferating cell nuclear antigen
Ssa#DW178930	DW178930	-4.7	down	sus scrofa breed tongcheng fascin
Ssa#STIR03416	gi|209735169	-4.7	down	fatty acid-bindingheart

Ssa#TC102152	TC102152	-4.7	down	insulin-like growth factor binding protein 4
Ssa#S30278507	S30278507	-4.7	down	CDC45-related protein
Ssa#STIR22117	TC84248	-4.8	down	interferon-induced transmembrane protein
Ssa#STIR05956	gi|209156079	-4.8	down	secreted frizzled-related protein 1
Ssa#CA061990	CA061990	-4.8	down	hypothetical protein LOC556012
Ssa#S35664683	EG899401	-4.8	down	DNA replication complex GINS protein PSF1
Ssa#TC113097	TC113097	-4.8	down	Transposable element Tcb1 transposase
Ssa#S35685879	S35685879	-4.8	down	PCNA-associated factor
Ssa#S35674060	S35674060	-4.8	down	oncorhynchus keta it-i gene for
Ssa#STIR00115_3	BT045917	-4.8	down	tropomyosin-1 alpha chain
Ssa#S35507110	S35507110	-4.8	down	Membrane-spanning 4-domains subfamily A member 4A
Ssa#STIR25856	TC89874	-4.8	down	---NA---
Omy#S15239165	S15239165	-4.8	down	UCMA_DICLARecName: Full=Unique cartilage matrix-associated protein;
Ssa#STIR10234	TC67023	-4.9	down	transmembrane protein 79
Ssa#S30239686	S30239686	-4.9	down	danio rerio zgc:194596 (zgc:194596) mrna
Ssa#STIR20782	TC82205	-4.9	down	Zebrafish DNA sequence from clone RP71-71M17 in linkage group 8
Ssa#TC84748	TC84748	-4.9	down	similar to CG8312-PA, isoform A
Ssa#STIR24268	TC87474	-4.9	down	hairy and enhancer of split 6
Ssa#STIR16537	TC75861	-4.9	down	c1q and tumor necrosis factor related protein 2
Ssa#STIR21285	TC82989	-4.9	down	glutamine synthetase
Ssa#S35592699	S35592699	-4.9	down	gasterosteus aculeatus clone cgx83-b03 mrna sequence
Ssa#S31997096	S31997096	-5.0	down	hypothetical protein LOC336057
Ssa#S30240822	S30240822	-5.0	down	minichromosome maintenance complex component 7
Ssa#STIR13279	TC71183	-5.0	down	origin recognitionsubunit 1-like
Ssa#STIR22644	TC85040	-5.0	down	---NA---
Ssa#STIR12350	TC69895	-5.0	down	---NA---
Ssa#S35582821	S35582821	-5.0	down	Serine protease HTRA1
Ssa#S27138402	S27138402	-5.0	down	rattus norvegicus atp-binding sub-family a member 12 mrna
Ssa#S35582068	S35582068	-5.1	down	Mimecan precursor
Omy#S34313259	S34313259	-5.1	down	hypothetical protein LOC573995
Omy#S34421918	S34421918	-5.1	down	similar to platelet endothelial aggregation receptor 1
Ssa#STIR18463	TC78730	-5.1	down	Oncorhynchus tshawytscha clone Ots.u6.23.15 genomic sequence
Ssa#S35663823	S35663823	-5.1	down	similar to c1q-like protein
Omy#BX864186	BX864186	-5.1	down	homo sapiens rad54-like (cerevisiae) transcript variant mrna
Ssa#S30295467	DW582431	-5.1	down	Kinetochore protein Spc24
Ssa#STIR15540	TC74416	-5.1	down	ribonucleotide reductase m1
Ssa#STIR16699	TC76108	-5.1	down	---NA---
Ssa#STIR25417	TC89205	-5.2	down	minichromosome maintenance complex component 7
Ssa#S35578853	S35578853	-5.2	down	Microfibrillar-associated protein 2 precursor
Ssa#S18834573	S18834573	-5.2	down	High choriolytic enzyme 1 precursor
Omy#S15296654	S15296654	-5.2	down	Proliferating cell nuclear antigen
Omy#S34425779	S34425779	-5.2	down	DNA polymerase subunit alpha B
Ssa#S35523399	EG809777	-5.2	down	cholesteryl ester transfer protein, plasma
Ssa#STIR21814	TC83788	-5.2	down	rab11 family interacting protein 1
Ssa#STIR22650	TC85053	-5.2	down	apolipoprotein a-i binding protein
Ssa#STIR11900	TC69277	-5.2	down	myosin ic
Ssa#STIR19276	TC79936	-5.2	down	---NA---
Ssa#S35564378	S35564378	-5.2	down	Myeloperoxidase precursor
Ssa#S30262006	S30262006	-5.2	down	novel protein similar to vertebrate cell division cycle associated 7 (CDCA7, zgc:110113)
Ssa#STIR23881	TC86901	-5.2	down	---NA---
Omy#TC168670	TC168670	-5.2	down	hypothetical protein LOC557997
Ssa#STIR06367	gi|209155253	-5.2	down	proliferating cell nuclear antigen
Ssa#EG917926	EG917926	-5.3	down	novel protein
Ssa#STIR15543	TC74419	-5.3	down	minichromosome maintenance complex component 7
Ssa#S35582042	S35582042	-5.3	down	Secreted frizzled-related protein 1
Ssa#STIR25794	TC89765	-5.3	down	sickle tail protein homolog
Ssa#S30277129	S30277129	-5.3	down	novel protein similar to vertebrate cell division cycle associated 7 (CDCA7, zgc:110113)
Ssa#STIR24935	TC88480	-5.3	down	---NA---
Ssa#TC108438	TC108438	-5.3	down	eph receptor B4a
Ssa#S18891168	S18891168	-5.4	down	5'-nucleotidase domain containing 2
Ssa#S35544695	S35544695	-5.4	down	Complement C1q tumor necrosis factor-related protein 5
Ssa#STIR14193	TC72506	-5.4	down	---NA---
Ssa#S31990975	S31990975	-5.4	down	multidrug resistance associated protein 2
Ssa#CA062730	CA062730	-5.4	down	N-acetyltransferase ESCO1
Ssa#S37580916	EF432866	-5.4	down	Insulin-like growth factor-binding protein 7 precursor
Ssa#STIR06184	gi|209155621	-5.4	down	dihydropyrimidinase-like 3
Ssa#STIR25320	TC89056	-5.4	down	dna-damage-inducible transcript 4-like
Ssa#STIR17618	TC77475	-5.4	down	---NA---
Ssa#STIR22458	TC84764	-5.4	down	---NA---
Omy#BX912760	BX912760	-5.5	down	: thrombospondin 2
Ssa#S30287399	S30287399	-5.5	down	Meiotic nuclear division protein 1 homolog
Omy#S15328778	S15328778	-5.5	down	mus musculus thrombospondin 1 mrna
Ssa#STIR23189	TC85862	-5.5	down	microfibrillar-associated protein 2
Ssa#CL175Ctg1	CL175Contig1	-5.6	down	Myeloperoxidase precursor
Ssa#TC70045	TC70045	-5.6	down	origin recognition complex subunit 5
Ssa#TC66657	TC66657	-5.6	down	Glycine cleavage system H protein, mitochondrial precursor
Ssa#S35659322	S35659322	-5.6	down	Pyridoxal kinase
Ssa#TC106165	TC106165	-5.7	down	AF510992_1lunatic fringe protein
Omy#S15297739	S15297739	-5.7	down	synthetic construct homo sapiens clone angiopoietin-like factor
Ssa#STIR13642	TC71724	-5.7	down	Zebrafish DNA sequence from clone CH211-88D2 in linkage group 22 Contains nuclear factor I/A (NFIA)
Ssa#KSS1055	KSS1055	-5.7	down	Annexin A4
Ssa#STIR24924	TC88463	-5.8	down	actin related protein 2 3subunit41kda
Ssa#DY694816	DY694816	-5.8	down	similar to carboxypeptidase X 1 (M14 family)
Ssa#STIR15320	TC74116	-5.9	down	betaine-homocysteine methyltransferase
Omy#S34424883	S34424883	-5.9	down	similar to p120-PI3K
Ssa#STIR20713	TC82100	-6.0	down	CNS0G5B8Tetraodon nigroviridis full-length cDNA
Ssa#S32011733	S32011733	-6.0	down	UDP-glucuronosyltransferase 2A2
Omy#S34425687	S34425687	-6.0	down	myocilin
Ssa#S31978672	S31978672	-6.1	down	similar to Adenosine deaminase domain-containing protein 2 (Testis nuclear RNA-binding protein-like)
Ssa#S31982108	S31982108	-6.1	down	carbonic anhydrase Xa
Ssa#AM402736	AM402736	-6.1	down	phosphatase and tensin-like protein B long splice variant
Ssa#STIR23969	TC87043	-6.1	down	---NA---
Ssa#S35553471	S35553471	-6.2	down	Guanine nucleotide-binding protein GI/GS/GO subunit gamma-2
Omy#S18157277	S18157277	-6.2	down	Ribonucleoside-diphosphate reductase large subunit
Ssa#STIR26075	TC90193	-6.3	down	---NA---
Ssa#STIR10992	TC68038	-6.3	down	chromosome transmission fidelity factor 8 homolog

Ssa#EG855563	EG855563	-6.3	down	retinal pigment epithelium-specific protein 65kDa
Ssa#STIR09291	TC65831	-6.3	down	---NA---
Ssa#STIR09701	TC66351	-6.3	down	sulfotransferase familycytosolic sulfotransferase 3
Ssa#STIR20402	TC81647	-6.3	down	---NA---
Ssa#TC74766	TC74766	-6.4	down	N-acetyltransferase ESCO1
Ssa#S31999264	S31999264	-6.4	down	mus musculus cdna clone image: containing frame-shift errors
Ssa#STIR02816	gi|209736373	-6.4	down	sulfotransferase familycytosolic sulfotransferase 3
Ssa#S18887170	S18887170	-6.4	down	salmo salar clone ssal-rgf-517-345 tripartite motif-containing protein 39 pseudogene cds
Ssa#STIR20000	TC81055	-6.4	down	Salmo salar clone ssal-rgf-531-250 Circumsporozoite protein precursor putative mRNA, pseudogene cds
Ssa#S31996507_S	S31996507	-6.4	down	Tartrate-resistant acid phosphatase type 5
Ssa#S35582944	S35582944	-6.4	down	Mimecan
Ssa#S31987867	S31987867	-6.5	down	danio rerio zgc:172167 (zgc:172167) mrna
Ssa#S35515107	S35515107	-6.5	down	Death-associated protein-like 1-B
Ssa#STIR19049	TC79604	-6.5	down	: Taeniopygia guttata mesenchyme homeobox 2 (LOC100219741), mRNA
Omy#CR363263	CR363263	-6.5	down	danio rerio immunoglobulin member 10 mrna
Ssa#STIR09623	TC66260	-6.5	down	seroreactive antigen bmn1-5
Ssa#STIR15986	TC75050	-6.5	down	Salmo salar clone ssal-rgf-525-167 Dehydrogenase/reductase SDR family member 13
Ssa#S35548045	S35548045	-6.6	down	Cellular retinoic acid-binding protein
Ssa#STIR14202	TC72520	-6.7	down	---NA---
Ssa#STIR22803	TC85283	-6.7	down	Salmo salar zonadhesin-like gene, and 3' UTR
Ssa#S18890448	CB516667	-6.7	down	DNA replication licensing factor mcm5
Ssa#S35472370	S35472370	-6.8	down	suppressor of variegation 3-9 homolog 1
Ssa#STIR15477	TC74330	-6.8	down	Salmo salar clone ssal-rgf-537-145 unknown large open reading frame mRNA, novel cds
Ssa#STIR12768	TC70477	-6.8	down	---NA---
Ssa#STIR20981	TC82517	-6.9	down	asparagine-rich protein
Ssa#S18891166	S18891166	-6.9	down	novel protein similar to vertebrate DNA replication helicase 2 homolog (yeast) (DNA2)
Omy#CX247666	CX247666	-6.9	down	salmo salar vesicle transport protein got1a mrna
Ssa#S31986243	S31986243	-7.0	down	danio rerio si:ch211- (si:ch211-) mrna
Ssa#S35500292	S35500292	-7.0	down	DNA-directed DNA polymerase epsilon
Ssa#S30289790	S30289790	-7.2	down	amine oxidase copper containing 3
Ssa#STIR24255	TC87455	-7.2	down	proliferating cell nuclear antigen
Ssa#STIR20131	TC81238	-7.4	down	placenta-specific 9
Ssa#S35532148	S35532148	-7.4	down	: sc:d0375
Omy#BX315869	BX315869	-7.4	down	bos taurus secreted frizzled-related protein 2 mrna
Ssa#STIR05353	gi|209731271	-7.5	down	thymidine kinasesoluble
Ssa#STIR21317	TC83046	-7.5	down	Zebrafish DNA sequence from clone CH211-88D2 Contains vertebrate nuclear factor I/A (NFIA),
Ssa#S30242995	S30242995	-7.5	down	CDC45-related protein
Ssa#S35661792	S35661792	-7.5	down	Complement factor D precursor
Ssa#S35564287	S35564287	-7.7	down	Thymidine kinase, cytosolic
Ssa#S35507200	S35507200	-7.7	down	High choriolytic enzyme 1
Ssa#STIR21004	TC82549	-7.8	down	cyclin e2
Ssa#TC107655	TC107655	-7.8	down	aldolase a, fructose-bisphosphate 1
Ssa#S35576104	S35576104	-7.9	down	Transcription factor Sp5
Ssa#STIR06110	gi|209155769	-7.9	down	cyclin e2
Ssa#S37580919	EF432861	-7.9	down	insulin-like growth factor binding protein 4
Ssa#STIR07485	TC63644	-8.0	down	chemokine (c-c motif) ligand 13
Ssa#STIR25838	TC89843	-8.1	down	fat tumor suppressor homolog 4
Ssa#STIR24236	TC87431	-8.2	down	Takifugu rubripes clone 214J14, complete sequence
Ssa#STIR20516	TC81806	-8.2	down	selenoprotein p precursor
Ssa#STIR13904	TC72099	-8.4	down	membrane-spanning 4-subfamilymember
Ssa#STIR15372	TC74187	-8.5	down	Salmo salar clone ssal-rgf-534-240 Collagen alpha-1I chain precursor putative mRNA
Ssa#TC101760	TC101760	-8.5	down	homo sapiens myoferlin transcript variant mrna
Ssa#DY734730	DY734730	-8.7	down	danio rerio misc_rna miscrna
Ssa#STIR14588	TC73084	-8.8	down	egf-containing fibulin-like extracellular matrix proteinisoform cra_a
Ssa#STIR21610	TC83486	-8.8	down	---NA---
Ssa#S35509181	S35509181	-8.9	down	similar to thrombospondin
Ssa#STIR11947	TC69348	-8.9	down	aquaporin 1
Ssa#S35480963	S35480963	-9.0	down	similar to adlican
Omy#S18532030	S18532030	-9.1	down	: X-ray radiation resistance associated 1
Ssa#S31981482	S31981482	-9.2	down	leucine rich repeat protein 1, neuronal
Ssa#STIR17548	TC77373	-9.6	down	Danio rerio lunatic fringe protein (lfng) mRNA,
Ssa#S35675030	S35675030	-9.6	down	AF510992_1lunatic fringe protein
Ssa#STIR16369	TC75609	-10.1	down	pigment epithelium-derived factor
Ssa#STIR20543	TC81835	-10.2	down	---NA---
Ssa#STIR40990	TC112395	-10.3	down	seraf-like protein
Ssa#STIR17937	TC77941	-10.3	down	Salmo salar clone 249L01 TCR-alpha/delta locus, genomic sequence
Omy#S15306626	S15306626	-10.3	down	canis familiaris sphingolipid c4-hydroxylase delta 4-desaturase mrna
Ssa#STIR13945	TC72157	-10.5	down	Zebrafish DNA sequence from clone CH211-197N10 Contains collagen, type VIII, alpha 2
Ssa#S30277130	DW564098	-10.6	down	novel protein similar to vertebrate cell division cycle associated 7 (CDCA7, zgc:110113)
Ssa#STIR08988	TC65437	-10.7	down	---NA---
Ssa#S35582593	EG868971	-10.9	down	sus scrofa type alpha 1 mrna
Ssa#STIR21636	TC83525	-11.1	down	protein
Omy#S15253152	S15253152	-11.2	down	amine oxidase copper containing 3
Omy#S34314498	S34314498	-11.6	down	similar to C1q and tumor necrosis factor related protein 2, partial
Ssa#STIR22652	TC85057	-12.1	down	Zebrafish DNA sequence from clone CH211-125A15 in linkage group 4
Ssa#S18887638	S18887638	-12.5	down	twist homolog 3
Ssa#STIR19407	TC80143	-12.6	down	---NA---
Omy#S18536169	S18536169	-12.7	down	DNA-directed DNA polymerase epsilon
Ssa#S35604422	S35604422	-13.3	down	dopamine receptor D1A4
Ssa#STIR12448	TC70037	-13.4	down	---NA---
Ssa#STIR05684	gi|209730609	-14.0	down	chemokine (c-c motif) ligand 13
Ssa#S30262878	S30262878	-14.3	down	hairy-related 4
Omy#CX246176	CX246176	-14.6	down	UDP-glucuronosyltransferase 2A2
Omy#S19711255	CR367985	-14.7	down	G1/S-specific cyclin-E2
Omy#S34311297	CU069027	-15.1	down	xenopus laevis cdc6-related protein mrna
Ssa#S35578867	S35578867	-15.3	down	Spondin-2
Ssa#S35546405	S35546405	-15.4	down	AF158374_1prostaglandin endoperoxide synthase-1; cyclooxygenase-1
Ssa#S35558725	S35558725	-15.5	down	Testis derived transcript (3 LIM domains)
Ssa#S35578867_S	S35578867	-16.0	down	Spondin-2
Ssa#STIR18941	TC79441	-16.1	down	coagulation factor iireceptor
Ssa#S35564994	EG851372	-16.2	down	danio rerio 35 mrna
Ssa#STIR13603	TC71664	-16.4	down	---NA---
Ssa#STIR14513	TC72974	-16.5	down	---NA---
Ssa#S35547171	S35547171	-16.6	down	Four and a half LIM domains protein 1
Ssa#STIR15933	TC74974	-17.4	down	Salmo salar clone ssal-rgf-503-341 Serine protease 23 precursor putative mRNA,
Ssa#STIR14896	TC73525	-18.1	down	claudin 6

Ssa#STIR22309	TC84523	-18.3	down	---NA---
Ssa#STIR16637	TC76003	-18.4	down	---NA---
Ssa#STIR04769	gi|209732451	-18.9	down	chemokine (c-c motif) ligand 13
Ssa#STIR14498	TC72949	-19.7	down	carboxypeptidase x 1 (m14 family)
Ssa#S18886546	S18886546	-19.9	down	High choriolytic enzyme 1
Ssa#S35480949	S35480949	-21.1	down	High choriolytic enzyme 1
Ssa#S35687715	EG922433	-22.7	down	Complement factor D precursor
Ssa#STIR19615	TC80473	-24.7	down	Salmo salar clone ssal-eve-565-204 C-C motif chemokine 25 precursor putative mRNA,
Ssa#S35545851	S35545851	-26.1	down	danio rerio im:7144089 (im:7144089) mrna
Ssa#STIR30922	TC97553	-27.9	down	Myf5 protein
Ssa#S35582146	S35582146	-33.4	down	danio rerio im:7144089 (im:7144089) mrna
Ssa#STIR23666	TC86594	-35.4	down	spondinextracellular matrix protein
Ssa#STIR12938	TC70726	-40.3	down	Salmo salar clone ssal-rgg-512-075 Acyl-CoA-binding protein putative mRNA,
Ssa#STIR05432	gi|209731113	-42.0	down	aquaporin 1
Ssa#S35524833	S35524833	-51.3	down	tetraodon nigroviridis full-length cdna
Ssa#TC112629	TC112629	-62.2	down	similar to Tc1-like transporase
Ssa#STIR19295	TC79968	-87.3	down	aquaporin 1
Ssa#STIR11656	TC68950	-125.0	down	aquaporin 1

Supplementary Table 1. Full list of mRNAs found to be up- and down-regulated in rIL-1β stimulated Atlantic salmon primary myocytes compared to control. The genes shown were significant at $p<0.05$ following t- tests and greater than 2-fold change. 1 Indicates the unique code for the feature on the microarray, 2 Accession number of the cDNA sequence, 3 Fold-change for genes altered in expression following feeding PP diet. 4 direction of regulation. 5 Identity of the probe target as determined by BlastX & BLASTN searches (NA indicates no homology found).
