# Supplementary figures and images for: Inflammatory responses in primary muscle cell cultures in Atlantic salmon (Salmo salar)
Source: BMC Genomics. 2013 Nov 1;14:747. doi: 10.1186/1471-2164-14-747 (PMC3819742; doi:10.1186/1471-2164-14-747)

## Slide 1
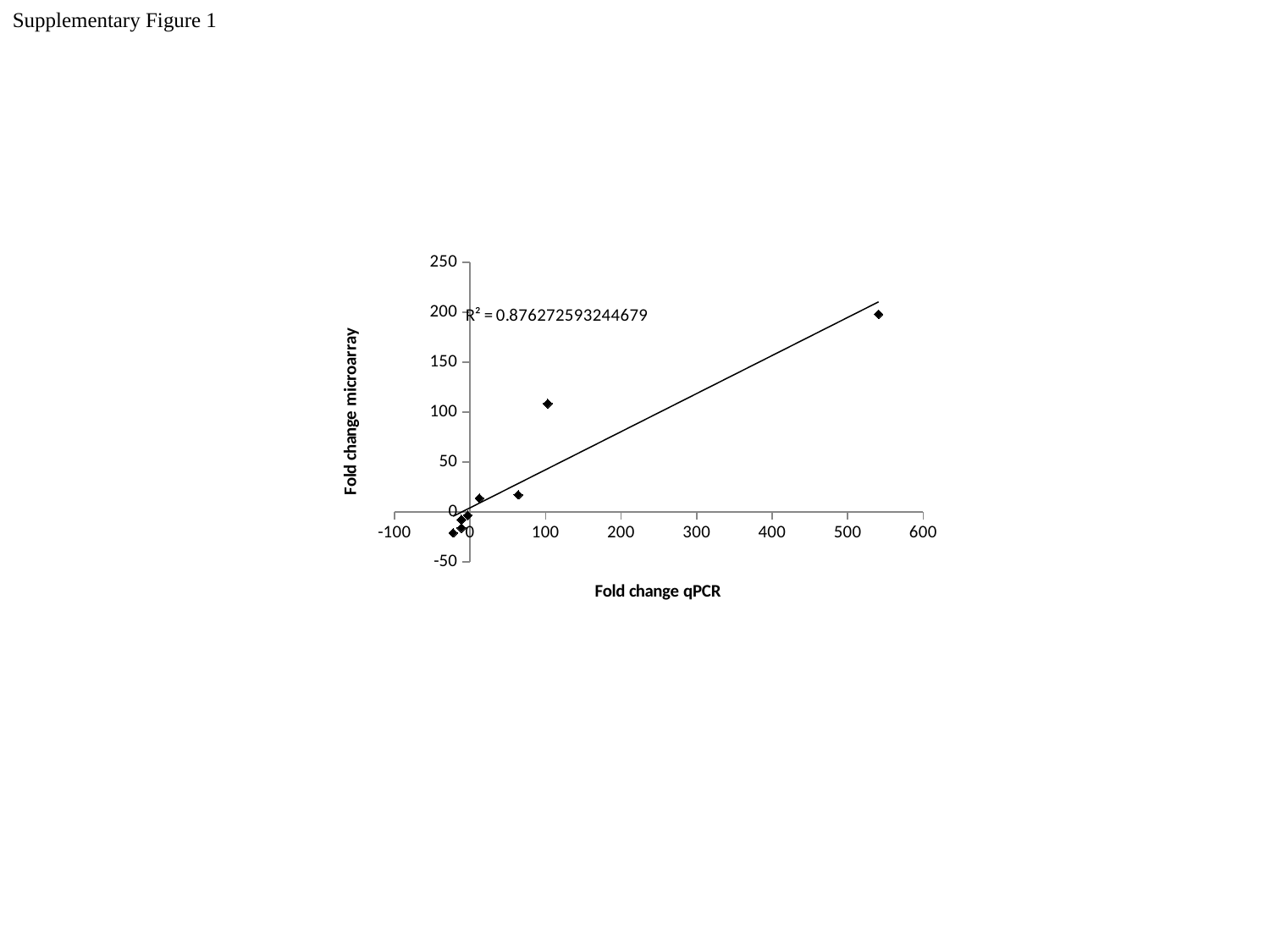

Supplementary Figure 1
### Chart
| Category | |
|---|---|

Supplement: Additional file 2: Figure S1 — Scatter plot showing comparative expression of genes from the microarray (n = 4) and qPCR (n = 4). The mean value was used in situations where a gene appeared multiple times on the microarray. Regression analysis found the expression levels for these 8 genes were significantly correlated between microarray and qPCR (p = 0.001). [file 1471-2164-14-747-S2.pptx]
